# Supplementary material for: KSTAR: An algorithm to predict patient-specific kinase activities from phosphoproteomic data
Source: Nat Commun. 2022 Jul 25;13:4283. doi: 10.1038/s41467-022-32017-5 (PMC9314348; doi:10.1038/s41467-022-32017-5)
Supplement: Supplementary file 1 — Supplementary Information [file 41467_2022_32017_MOESM1_ESM.pdf]

Supplementary Information

KSTAR: An algorithm to predict patient-specific kinase activities from phosphoproteomic data

Sam Crowl[1,†],Ben T. Jordan[1,†], Hamza Ahmed[1], Cynthia X. Ma[2], Kristen M. Naegle[1,*]

1 University of Virginia, Department of Biomedical Engineering and the Center for Public Health Genomics, Charlottesville, VA, 22903
2 Department of Medicine and Siteman Cancer Center, Washington University in St. Louis, St. Louis, Missouri 63108

* kmn4mj@virginia.edu
† authors contributed equally to this work

This file includes:

Supplementary Note 1: Assessing the impact of pruning and parameter selection in KSTAR (Pages 2-5)
Supplementary Note 2: Controlling for kinase- and experiment-specific false positive rates (Pages 6-14)
Supplementary Note 3: Full KSTAR Predictions on Control Datasets (Pages 15-22)
Supplementary Note 4: Comparing KSTAR to other available activity inference algorithms (Pages 23-34)
Supplementary Note 5: Robustness analysis comparing NSCLC and CML cell lines from independent experiments (Pages 35-37)
Supplementary Note 6: Full analysis results of breast cancer phosphoproteomic datasets (Pages 38-49)
Supplementary Tables 1-7: (Pages 51-58)
Supplementary Methods: (Pages 59-68)
Supplementary References: (Pages 69-71)

Supplementary Note 1: Assessing the impact of pruning and parameter selection in KSTAR

Goal

1. Profile the impact of the heuristic prune on the characteristics of the kinase-substrate network

2. Explore the impact of applying different cutoffs to experimental datasets when determining which phosphorylation sites to use as evidence.

Methods

For comparison to commonly used kinase-substrate networks, kinase-substrate annotations were downloaded from PhosphoSitePlus [1]. The full NetworKIN prediction graph was downloaded for the entire phosphoproteome and edges with weights less than 1 were removed [2]. A threshold of 1 was selected in order to balance the number of edges that are removed from the network and the inaccuracy often observed at low thresholds.

To look at sensitivity of KSTAR results to different evidence cutoffs in phosphoproteomic experiments, we generated kinase activity predictions using different evidence sizes (number of phosphorylation sites identified in original experiment used for prediction) for a tyrosine dataset [3] and a serine/threonine dataset [4]. In both cases, the first test used all of the sites identified in the experiment, and subsequent tests with smaller evidence sizes removed the least abundant sites from analysis to mimic the effect of thresholding.

Table of Contents

Supplementary Figure 1 - Comparing the characteristics of pruned networks to PhosphoSitePlus and NetworKIN (Page 3)
Supplementary Figure 2 - Assessing the stability of tyrosine predictions using different amounts of sites as evidence, mimicking the effect of applying a threshold to the phosphoproteomic data to select phosphosites used as evidences (Page 4)
Supplementary Figure 3 - Assessing the stability of serine/threonine predictions using different amounts of sites as evidence, mimicking the effect of applying a threshold to the phosphoproteomic data to select phosphosites used as evidence (Page 5)

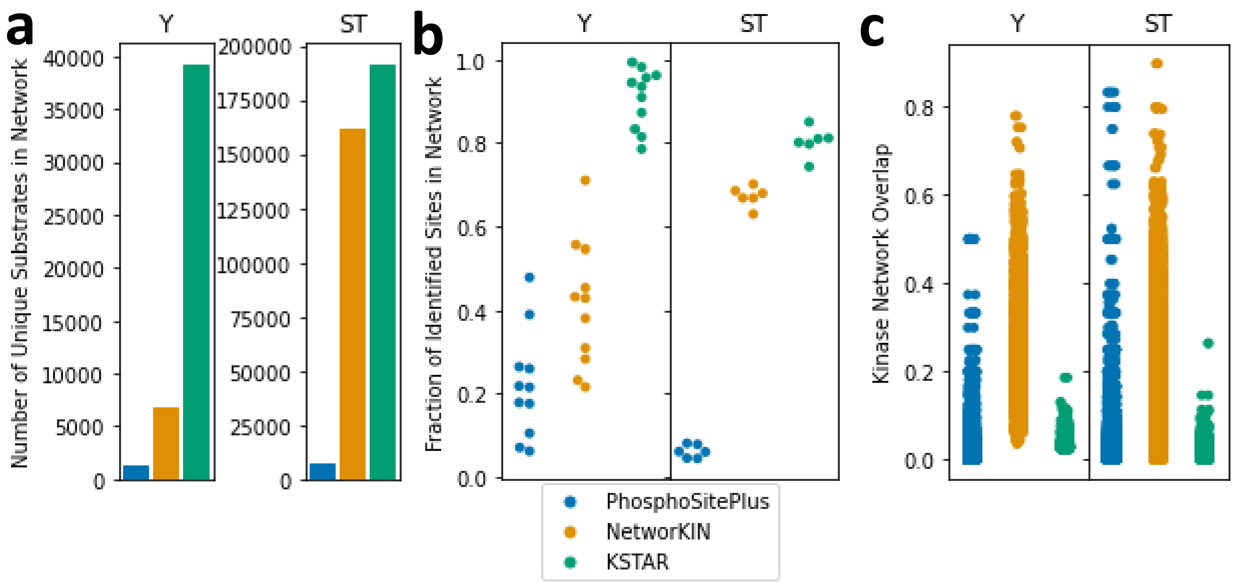

Supplementary Figure 1. Comparison of KSTAR pruned networks to PhosphoSitePlus and NetworKIN Comparison of network characteristics for known kinase-substrate annotations from PhosphoSitePlus (blue) [1], kinase-substrate predictions from NetworKIN thresholded with a value of 1 (remove edges with edge weights less than 1)(orange) [2], and the KSTAR ensemble of pruned networks (green). **A)** Number of unique substrates found within each network. **B)** Fraction of sites identified within an experiment that are also found within the kinase-substrate network. Experiments include phosphoproteomic dataset for predictions found in Figures 2, 5, and 6 of the main text. Each point on the plot represents a unique experiment (Tyrosine: n = 11 independent biological experiments, Serine/Threonine: n = 6 independent biological experiments). While prediction algorithms using either PhosphoSitePlus or NetworKIN will often have to drop $> 50\%$ of sites identified in an experiment, KSTAR typically loses $< 20\%$. This problem is especially apparent in tyrosine networks. **C)** Pairwise network similarity between different kinases in each network, defined by the Jaccard similarity, calculated as $JI = \frac{\text{total number of substrates connected to both kinases}}{\text{total number of substrates of connected to either kinase}}$. Each point represents a different pairwise comparison between kinases in the kinase-substrate network [PhosphoSitePlus (Y): n = 7131, PhosphoSitePlus (ST): n = 54,940, NetworKIN (Y): n = 1225, NetworKIN (ST): n = 9730, KSTAR (Y): n = 1225, KSTAR (ST): n = 9730]. Unlike PhosphoSitePlus and NetworKIN, the majority of kinases share $< 20\%$ of substrates in KSTAR networks. Source data are provided as a separate Source Data file with this paper.

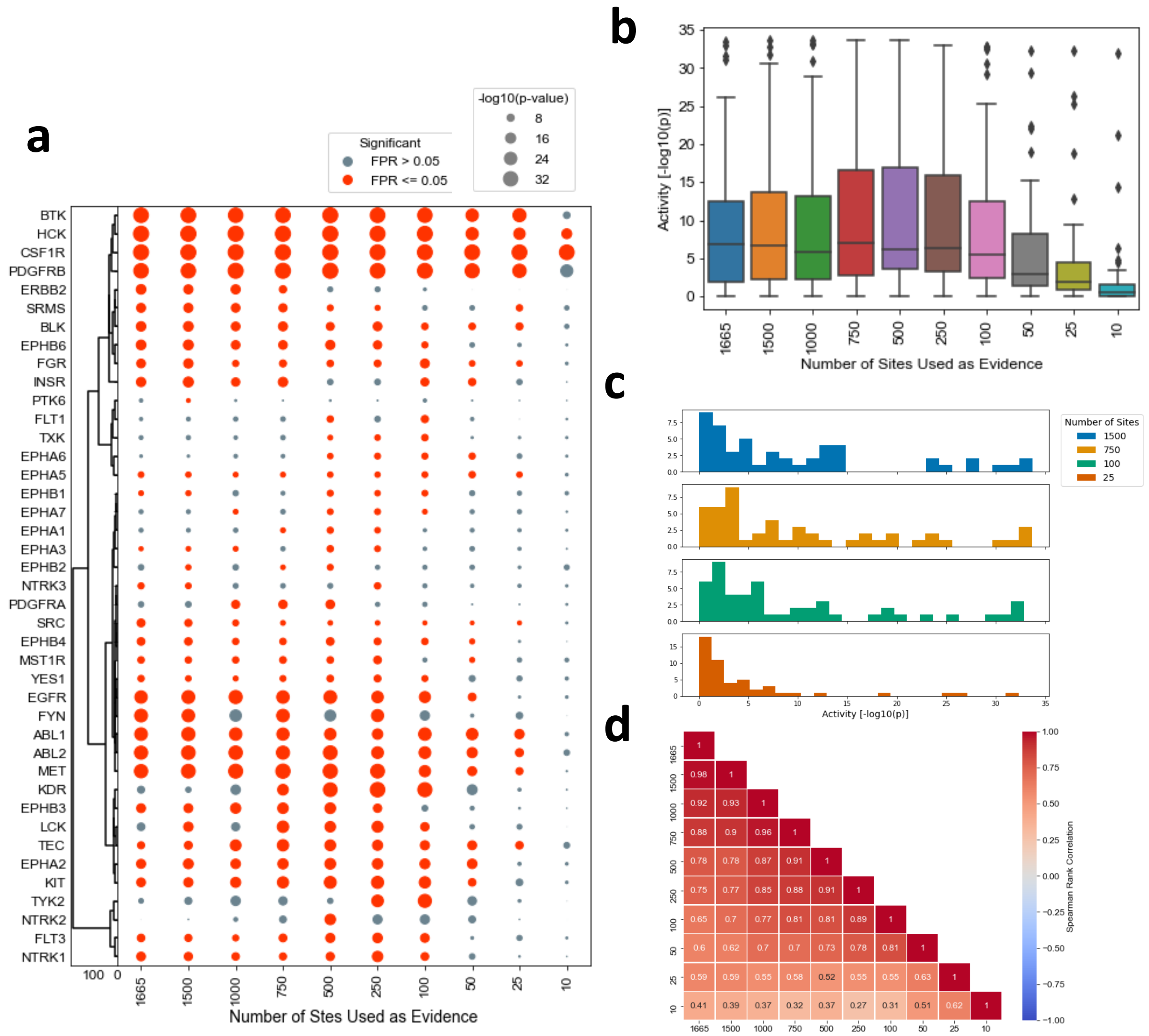

Supplementary Figure 2. Impact of experiment threshold on tyrosine kinase predictions Using control data of K562 chronic myeloid leukemia cell lines [3], we assessed variance in KSTAR predictions for tyrosine kinases using different thresholds (cutoff value to deterimine whether a phosphorylation site should be included in evidence). The threshold determines the total number of sites used as evidence for prediction. For this test, predictions were based evidence sizes ranging from 10 - 1665 (all identified) sites. **A)** KSTAR predictions for kinases that have predicted activity in at least one test ($FPR \leq 0.05$). **B)** Distribution of KSTAR activity scores for all kinases at each evidence size. Box indicates median (center line), 25th and 75th percentiles (box boundaries), 1.5x the IQR of the box edge (whiskers), and any outliers beyond 1.5x IQR (points). If no outliers exist, whiskers indicate maxima or minima. For each condition, statistics were derived from all tyrosine kinases with predictions in KSTAR (n = 50). **C)** Histograms demonstrating the distribution of activity scores when 25, 100, 750, 1500 sites are used for prediction. **D)** Correlation of activity rankings between predictions of different evidence sizes. Source data are provided as a separate Source Data file with this paper.

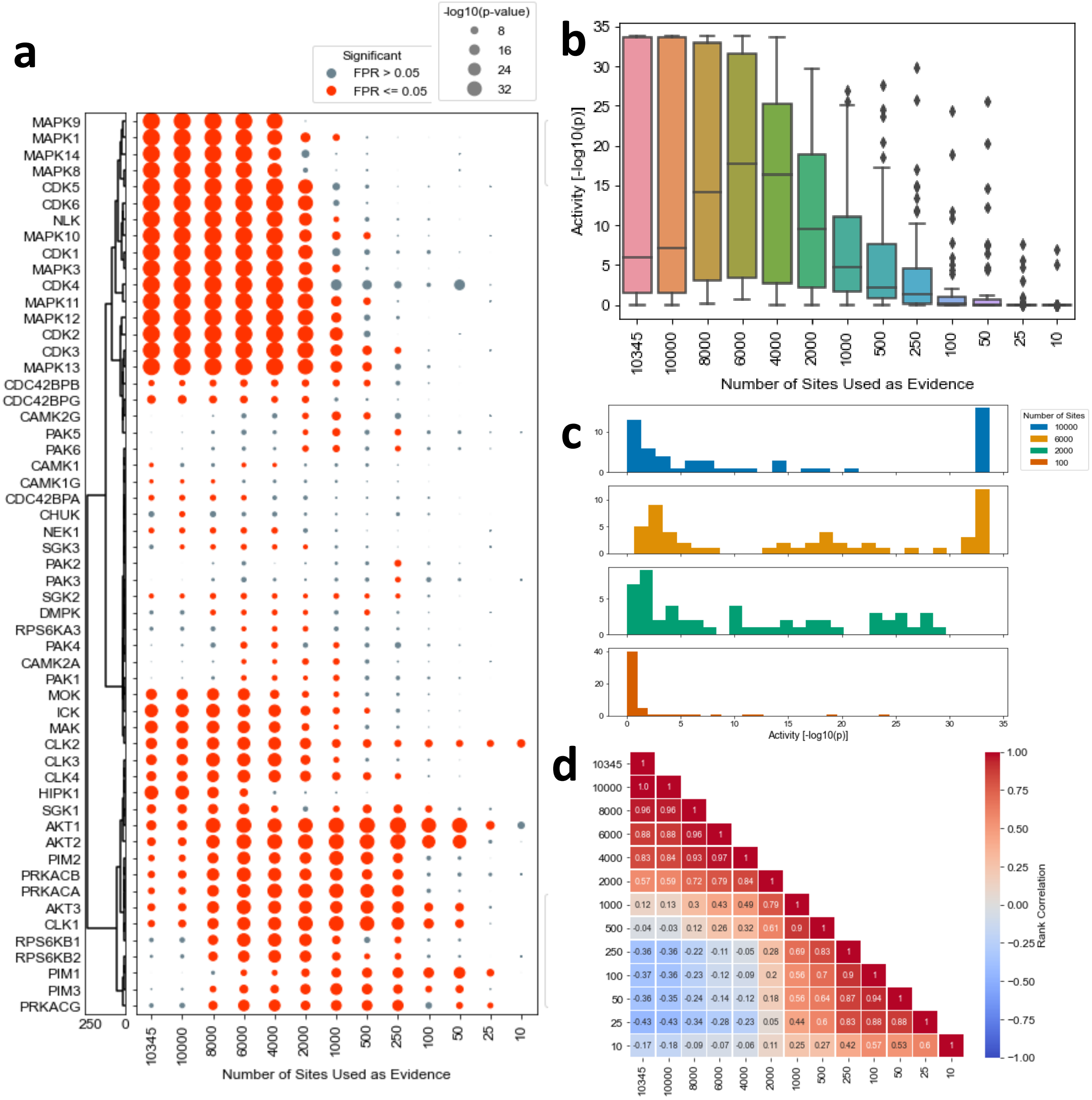

Supplementary Figure 3. Impact of experiment threshold on serine/threonine kinase predictions Using control data of BT-474 Breast cancer cell lines [4], we assessed variance in KSTAR predictions for serine/threonine kinases using different thresholds (cutoff value to deterimine whether a phosphorylation site should be included in evidence). The threshold determines the total number of sites used as evidence for prediction. For this test, predictions were based on evidence sizes ranging from 10 - 10345 (all identified) sites. **A)** KSTAR predictions for kinases that have predicted activity in at least one test ($FPR \leq 0.05$). **B)** Distribution of KSTAR activity scores for all kinases at each evidence size. Box indicates median (center line), 25th and 75th percentiles (box boundaries), 1.5x the IQR of the box edge (whiskers), and any outliers beyond 1.5x IQR (points). If no outliers exist, whiskers indicate maxima or minima. For each condition, statistics were derived from all serine/threonine kinases with activity in at least one condition in KSTAR (n = 55). **C)** Histograms demonstrating the distribution of activity scores when 100, 2000, 6000, or 10000 sites are used for prediction. **D)** Correlation of activity rankings between predictions of different evidence sizes. Source data are provided as a separate Source Data file with this paper.

Supplementary Note 2: Controlling for kinase- and experiment-specific false positive rates

Goal

1. Assess the bias found within different phosphoproteomic databases by predicting activity from random samplings of the database
2. Assess the bias found with phosphoproteomic datasets by looking at the distribution of study bias across the sites identified in an experiment
3. Assess the relationship between study bias of a substrate and quantified log fold changes

Methods

In order to measure the false positive rate, we randomly created 200 random datasets, each composed of 250 randomly selected phosphotyrosine sites using different phosphoproteomic databases as the background. We set the desired false positive rate at 0.05 and therefore expect, on average, about 10 positives per kinase across all datasets. The false positive results are from KSTAR networks before the final addition of controlling for the distribution of compendia was added (Supplementary Figures 4 -8).The size of the dataset was selected based on common dataset sizes for phosphotyrosines and this value is much smaller than the smallest of the compendia background.

Summary of Results

The first four figures here have been ordered from backgrounds that produce the highest false positive rates to those that produce the lowest false positive rates. It became clear that the kinases with high false positive rates were consistent across all compendia (except those from PhosphoSitePlus, which is the largest and predominantly comprised of mass spectrometry identified sites). In short, FYN, LCK, and HCK demonstrated significant levels (100%) false positive rates. On the other end of the range, it became clear that most kinases were producing less than expected false positive rates (0%), which suggested that it is also more difficult to yield true positives for these kinases. Additionally, we noticed a trend that the false positive rates were highest from the compendia that overlapped most with the genesis of the NetworKIN prediction models (Phospho.ELM [5], Supplementary Figure 4) and lowest from the compendia most separated from training data that formed the networks (PhosphoSitePlus [1], Supplementary Figure 7). We therefore hypothesized that there was a direct connection between the study bias of the phosphorylation sites (the more compendia a site is in the more likely it is annotated and was used in training the networks). Supplementary Figure 8 shows that our original KSTAR networks that generated such kinase-specific false positive rates indeed showed skew for more studied substrates connected to the kinases yielding high false positive rates. We next asked if we could measure the false positive rate for random experiments by random draws from the human phosphoproteome. We found that all experiments analyzed in this manuscript were not representative of random samples from the phosphoproteome, with respect to how many different compendia each phosphorylation site is stored (Supplementary Figure 10). We also found that magnitude of fold changes (i.e. quantification) was partially related to the study bias of the individual substrate (Supplementary Figure 11). This suggests that each experiment will have different false positive rates, influenced directly by how well studied the sites are within the dataset. This motivated the final mitigation strategy of selecting random datasets for measuring empirical false positive rates based on sampling sites from the phosphoproteome, such thatt he distribution matches that of the real experiment. The combination of avoiding quantification, normalizing kinase study bias distribution (Supplementary Figure 9), and sampling random sets to control for experiment-specific distributions, helps to control the false positive rate as desired in a kinase- and experiment-specific manner.

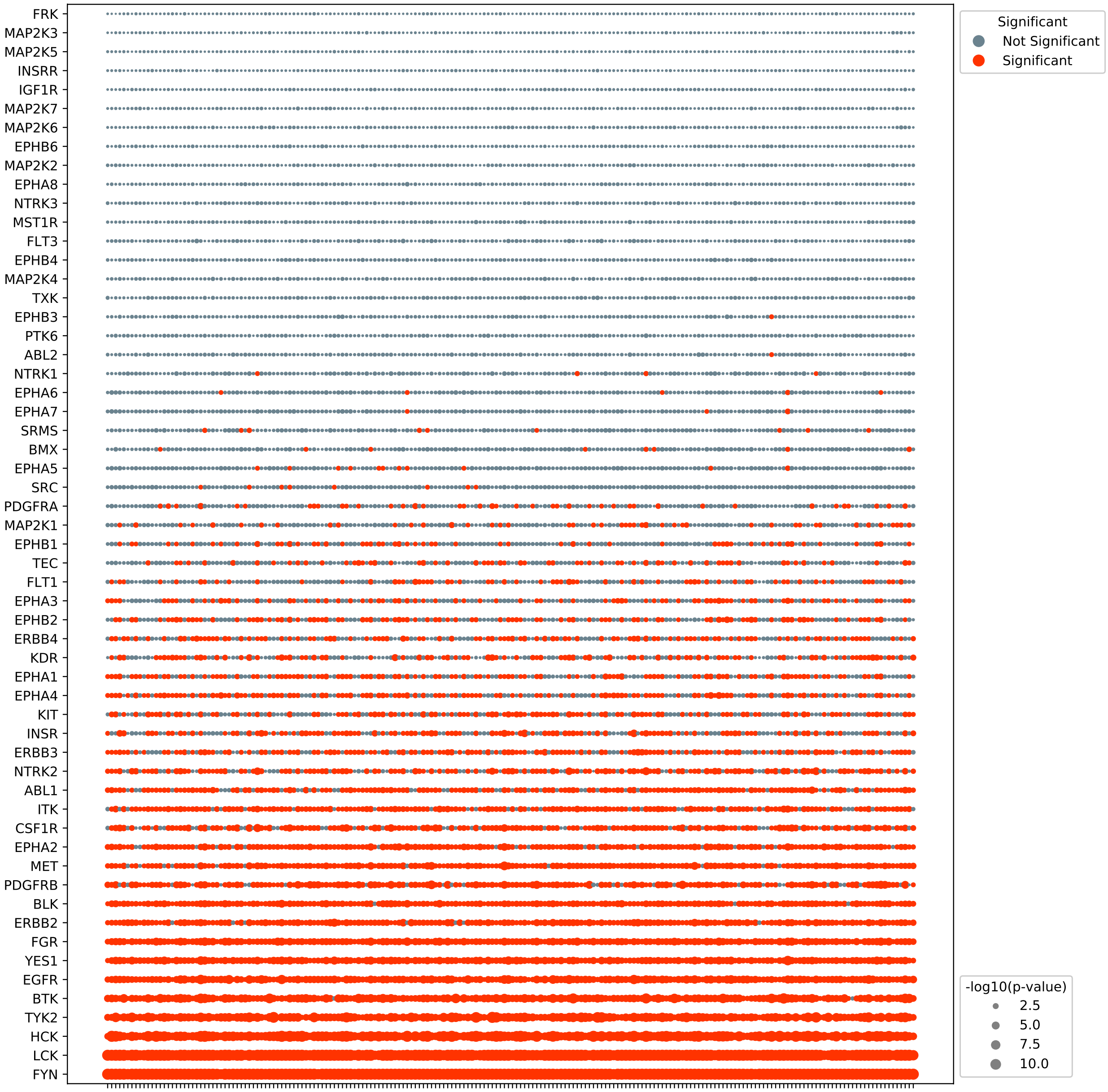

Supplementary Figure 4. Kinase-Specific False Positive Rates in PhosphoELM To assess false positive rates obtained from Phospho.ELM [5], 200 random datasets were created by randomly sampling 250 phosphotyrosine sites from PhosphoELM, and KSTAR was applied (without accounting for study bias, i.e. no study bias constraint in heuristic prune or comparison to random activities from Mann Whitney U test) to generate activity predictions for each random dataset. Each column in the above dotplot corresponds to results from a different random datset. The size of each dot corresponds to the median p-value enrichment obtained across all pruned networks, and is colored based on significance ($p <= 0.05$). Many kinases, including FYN, LCK, and HCK exhibited close to a 100% false positive rate, while others like FRK and INSRR were not predicted active in a single random dataset, lower than the expected 5% false positive rate. Source data are provided as a separate Source Data file with this paper.

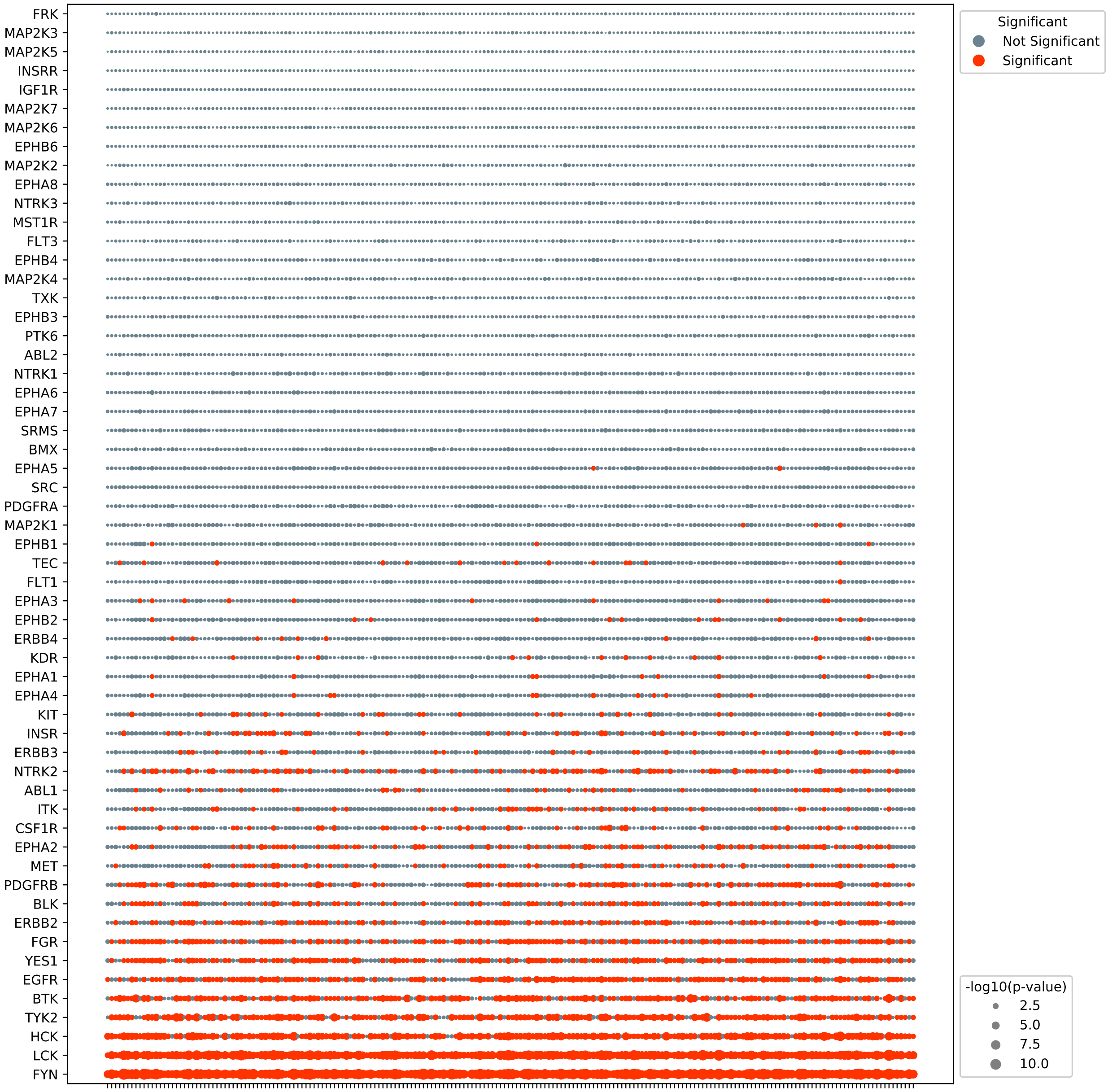

Supplementary Figure 5. Kinase-Specific False Positive Rates in HRPD To assess false positive rates obtained from HPRD [6], 200 random datasets were created by randomly sampling 250 phosphotyrosine sites from HPRD, and KSTAR was applied (without accounting for study bias, i.e. no study bias constraint in heuristic prune or comparison to random activities from Mann Whitney U test) to generate activity predictions for each random dataset. Each column in the above dotplot corresponds to results from a different random datset. The size of each dot corresponds to the median p-value enrichment obtained across all pruned networks, and is colored based on significance ($p <= 0.05$). Similar to Figure 1, many kinases, including FYN, LCK, and HCK exhibited close to a 100% false positive rate, while others like FRK and INSRR were not predicted active in a single random dataset, lower than the expected 5% false positive rate. Source data are provided as a separate Source Data file with this paper

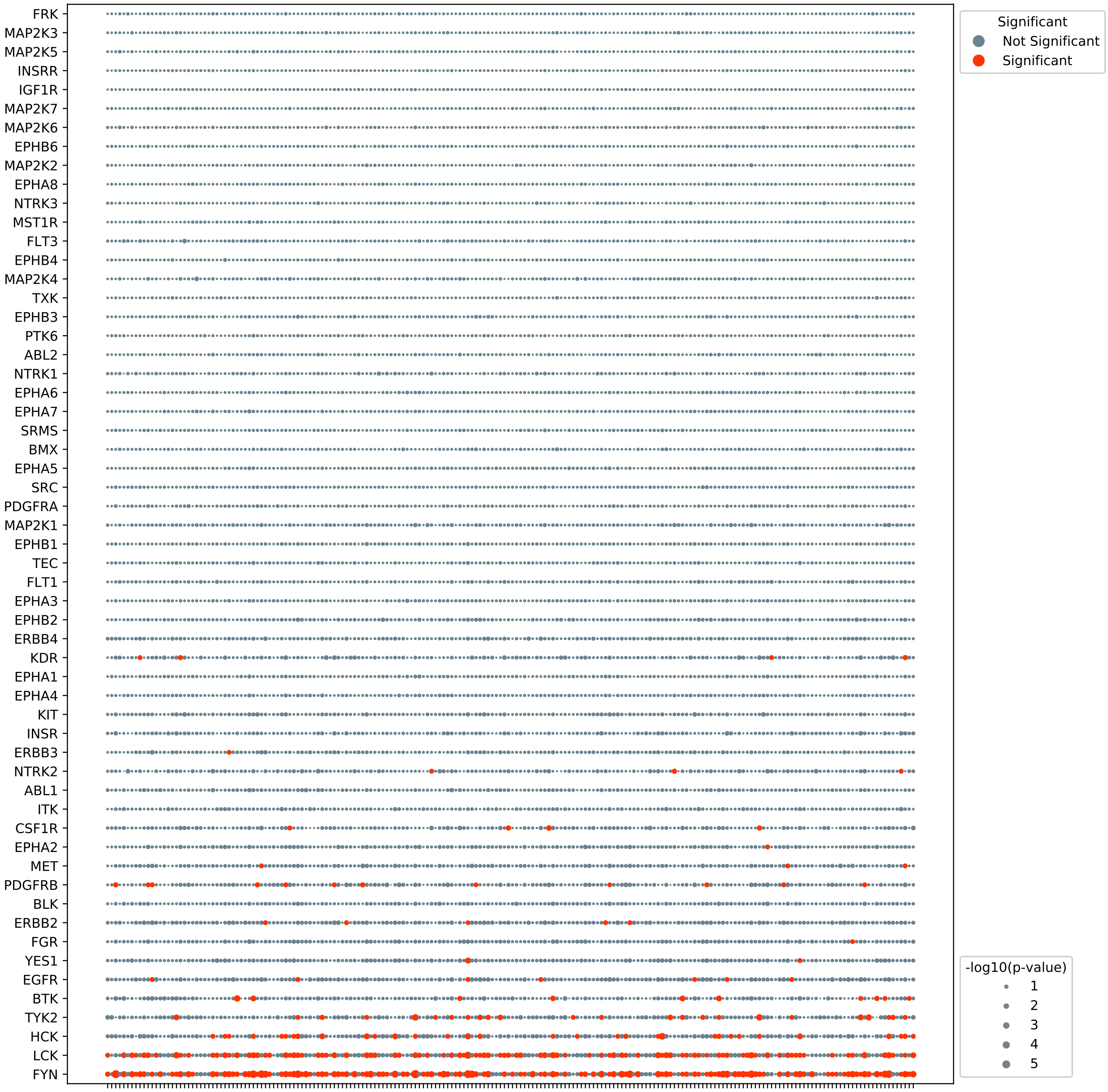

Supplementary Figure 6. Kinase-Specific False Positive Rates in dbPTM To assess false positive rates obtained from dbPTM [7], 200 random datasets were created by randomly sampling 250 phosphotyrosine sites from dbPTM, and KSTAR was applied (without accounting for study bias, i.e. no study bias constraint in heuristic prune or comparison to random activities from Mann Whitney U test) to generate activity predictions for each random dataset. Each column in the above dotplot corresponds to results from a different random datset. The size of each dot corresponds to the median p-value enrichment obtained across all pruned networks, and is colored based on significance ($p <= 0.05$). Predictions exhibit lower false positve rates than when sampling from HPRD [6] or Phospho.ELM [5], but still see high kinase-specific false-positive rates for kinases like FYN, LCK, and HCK, and sees more kinases with 0 positives across all datasets. Source data are provided as a separate Source Data file with this paper

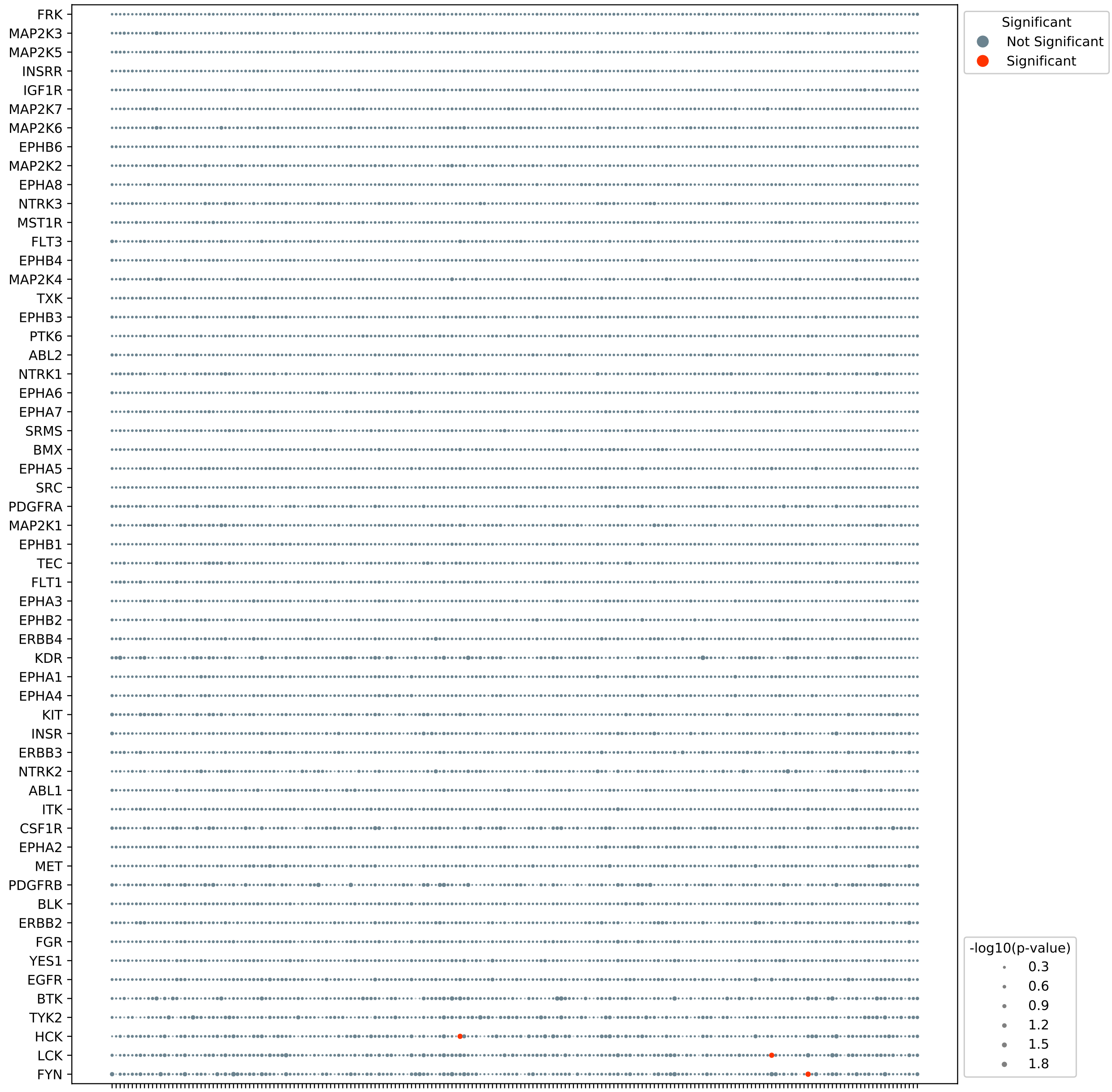

Supplementary Figure 7. Kinase-Specific False Positive Rates in PhosphoSitePlus To assess false positive rates obtained from PhosphoSitePlus [1], 200 random datasets were created by randomly sampling 250 phosphotyrosine sites from PhosphoSitePlus, and KSTAR was applied (without accounting for study bias, i.e. no study bias constraint in heuristic prune or comparison to random activities from Mann Whitney U test) to generate activity predictions for each random dataset. Each column in the above dotplot corresponds to results from a different random datset. The size of each dot corresponds to the median p-value enrichment obtained across all pruned networks, and is colored based on significance ($p <= 0.05$). All kinases show lower than expected false positive rates (0 instead of 10 positives) when sampled from PhosphoSitePlus as the background. PhosphoSitePlus is the largest of the compendia and derived predominantly of mass spectrometry identified sites from within the Cell Signaling Technology pipelines. Source data are provided as a separate Source Data file with this paper

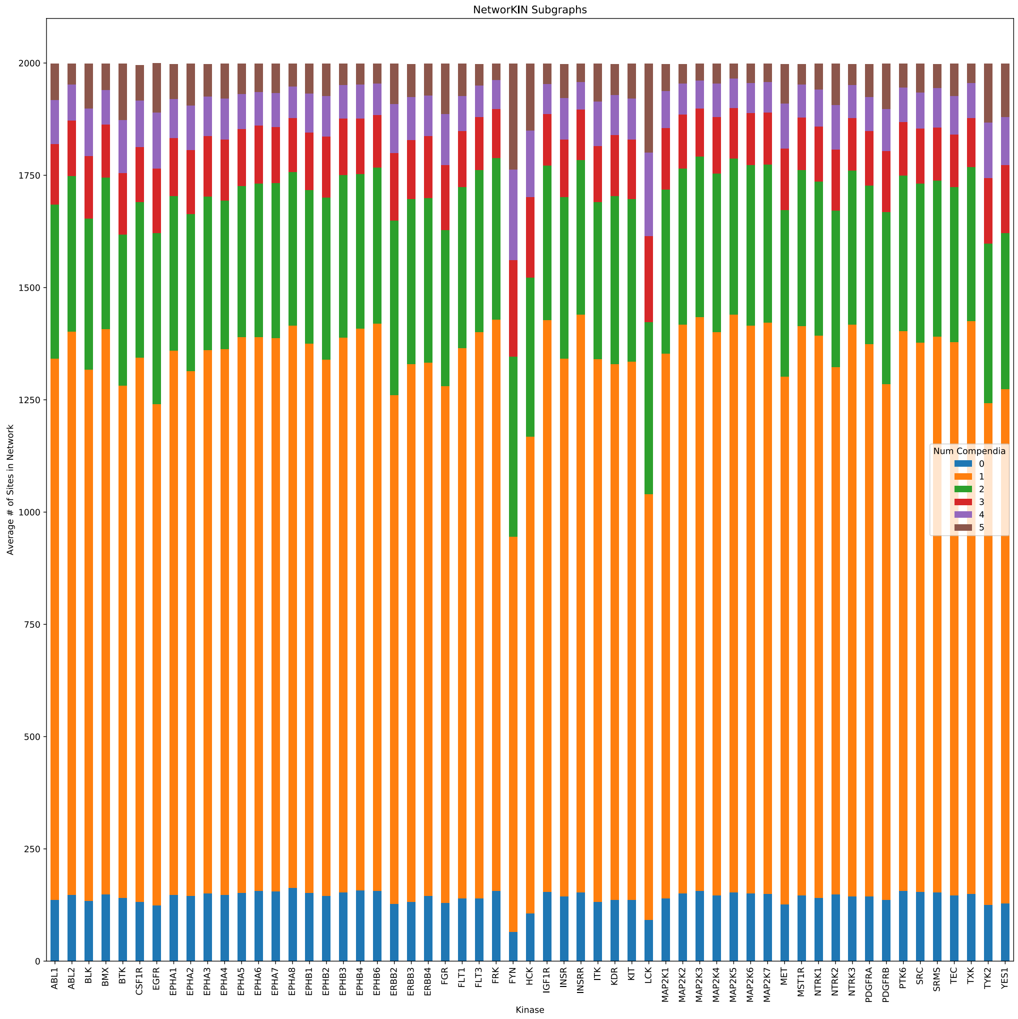

Supplementary Figure 8. Distribution of substrate study bias in original networks We plotted distribution of substrates, based on the number of compendia they are observed in, for our original KSTAR generated networks (no study bias constraint, Algorithm 1 in Supplementary Methods) and found that the high false positive rates of kinases correlated with kinases highly connected to well studied phosphorylation sites. For example, FYN, LCK, and HCK have the highest proportion of phosphorylation sites from three or more compendia and are the kinases with the highest degree of false positives. The above plot represents the average number of substrates found in each study bias grouping (found in 0, 1 ,2, 3, 4, or 5 compendia) across all of the 50 generated networks.

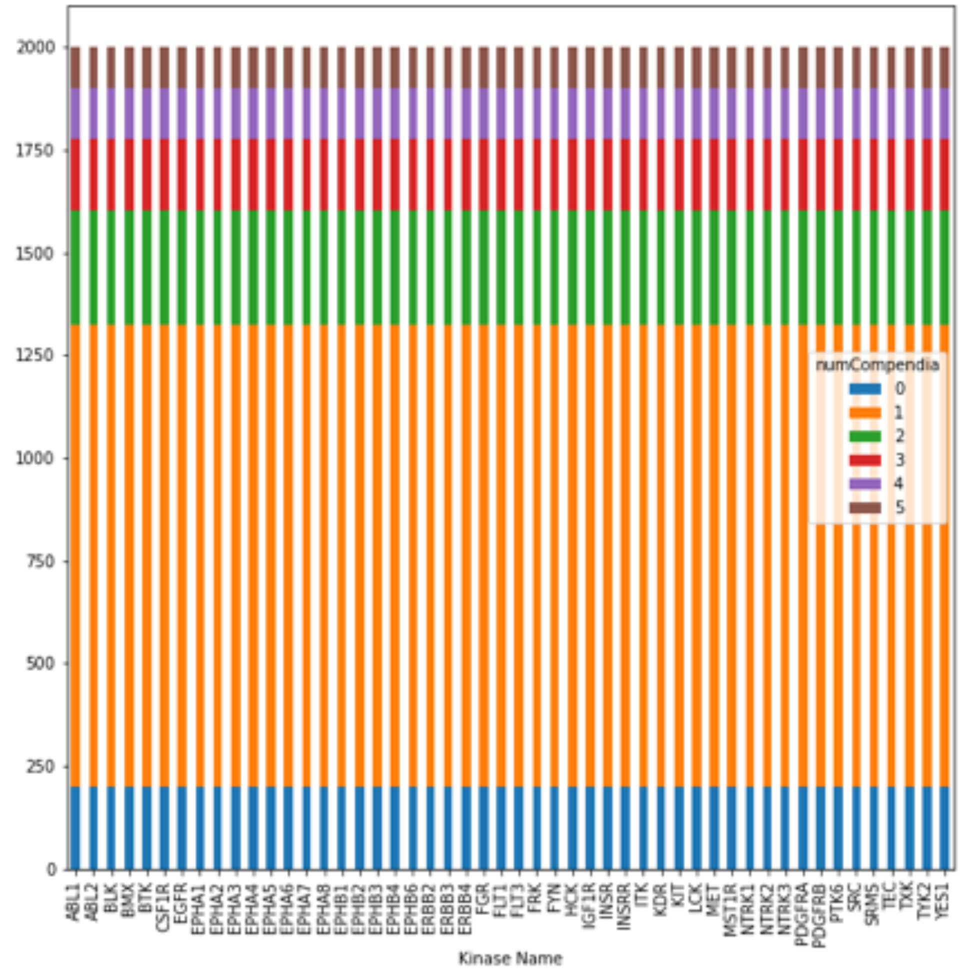

Supplementary Figure 9. Distribution of study bias in final networks We added the constraint that all kinases should be connected to substrates with an equal distribution of study bias, as defined by the number compendia they are observed in. This ensures that no kinase will be connected to more well studied sites than any other kinase, which leads to kinase-specific false positive rates. This leads to a more balanced network, as observed in the above plot indicating the average number of substrates found in each study bias grouping (found in 0, 1, 2, 3, 4, or 5 compendia) across the final KSTAR generated networks (with a study bias constraint, Algorithm 2 in Supplementary Methods).

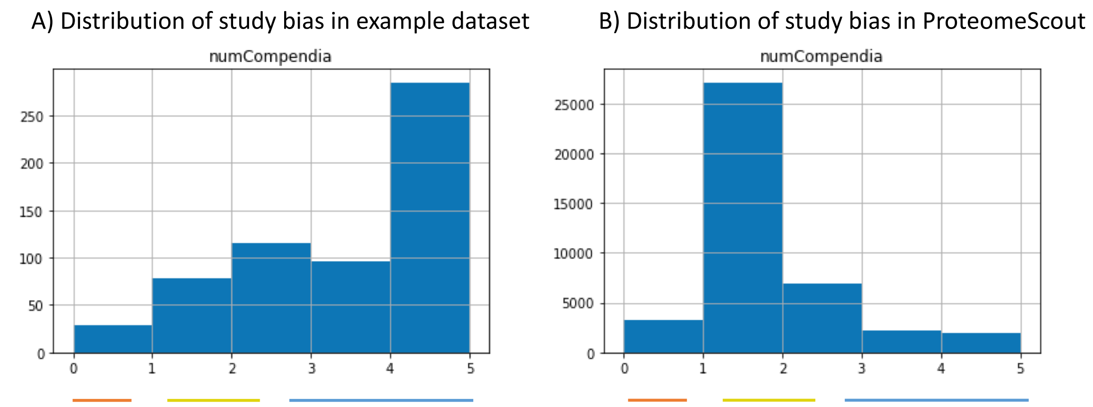

Supplementary Figure 10. Distribution of study bias in a real dataset, compared to whole phosphoproteome We found that the phosphorylation sites from experiments were much more likely to contain sites that are well annotated than expected by random chance (i.e. contributing to experiment-specific study bias). **A)** This is the histogram of the number of compendia phosphorylation sites are observed in for the phosphotyrosines of the PDX dataset from Huang et al. [8]. **B)** This is the distribution for all phosphotyrosines in the human phosphoproteome, which shows the majority of sites would be annotated by only one compendia (most likely, these are annotated only in PhosphoSitePlus). The three lines represent our final classes of study bias for low (0 compendia), medium (1-2 compendia), and high (more than 3 compendia). This classification was defined and published in our prior work [9]. Source data are provided as a separate Source Data file with this paper.

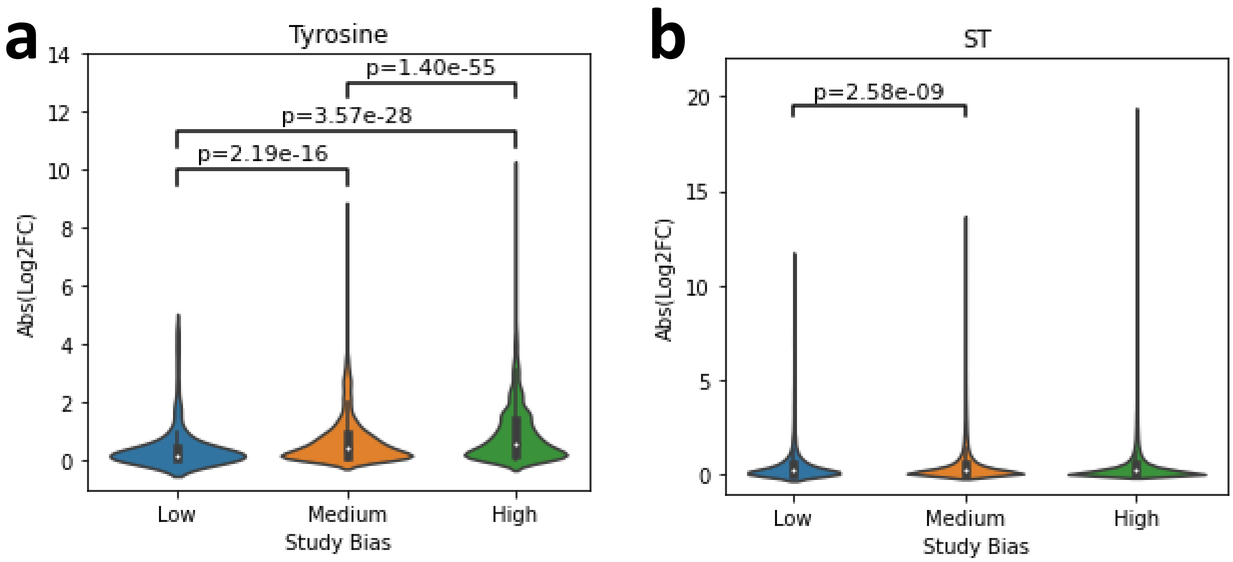

Supplementary Figure 11. Relationship between study bias and quantification in the benchmarking dataset In addition to the likelihood of identification discussed in Supplementary Figure 10, we explored whether quantification and study bias are related by looking at the distribution of fold change magnitudes as a function of study bias. We have defined three classes of study bias based on the number of compendia a site is identified in: low (0 compendia), medium (1-2 compendia), and high (more than 3 compendia). This classification was defined and published in our prior work, KinPred [9]. **A)** Distribution of log2fold changes for phosphorylated tyrosine sites based on degree of study bias. Quantification was obtained from the tyrosine kinase benchmarking dataset described in Supplementary Table 3 (20 independent experiments, Low: n = 253 sites, Medium: n = 7188 sites, High: n = 10,464).Violin plots indicate the median (white dot), 25th and 75th percentile (box edges), and 1.5x the IQR of the box edge (whiskers). Statistical significance was assessed using a one-tailed Mann Whitney U test and associated p-values are indicated on the plot. We found that well studied tyrosine sites identified in an experiment tend to exhibit larger log2 fold changes than less well studied sites. **B)** Distribution of log2fold changes for phosphorylated serine or threonine sites based on degree of study bias. Quantification was obtained from the serine/threonine kinase benchmarking dataset described in Supplementary Table 4 (33 independent experiments, Low: n = 3933 sites, Medium: n = 95,243 sites, High: n = 176,849 sites). Violin plots indicate the median (white dot), 25th and 75th percentile (box edges), and 1.5x the IQR of the box edge (whiskers). Statistical significance was assessed using a one-tailed Mann Whitney U test and p-values indicated on the plot if $p < 0.4$. We did not find as strong a relationship between study bias and quantification in serine/threonine datasets. Source data are provided as a separate Source Data file with this paper.

Supplementary Note 3: Full KSTAR Predictions on Control Datasets

Goal

Provide the full KSTAR predictions on activation and inhibition datasets discussed in Figure 2 of the main text.

Methods

For all inhibition and activation datasets where KSTAR was applied (Figure 2 in the main text and Supplementary Table 2), a dotplot is provided that includes all kinases with predictions (limited by the kinases with substrate predictions in NetworKIN). For the two serine/threonine datasets (Figure 5, 6), only kinases with significant activity in at least one sample ($FPR \leq 0.05$) were included in the plot due to space constraints.

Table of Contents

Supplementary Figure 12 - Full activity predictions during EGF stimulation of 184A1 epithelial cells (Page 16)
Supplementary Figure 13 - Full activity predictions during EGF/HRG stimulation of 184A1 epithelial cells overexpressing HER2, corresponding to Figure 2A (Page 17)
Supplementary Figure 14 - Full activity predictions during TCR activation of Jurkat cells, corresponding to Figure 2B (Page 18)
Supplementary Figure 15 - Full activity predictions during BCR-ABL inhibition by dasatanib in chronic myeloid leukemia cells, corresponding to Figure 2C (Page 19)
Supplementary Figure 16 - Full activity predictions after AKT Inhibition in breast cancer cells with 5 different AKT inhibitors, corresponding to Figure 2D (Page 20)
Supplementary Figure 17 - Full activity predictions after BRAF inhibition in two different colorectal cancer cell lines leading to paradoxical MAPK activation, corresponding to Figure 2E (Page 21)
Supplementary Figure 18 - Quantile-normalized activity scores for MAPK1 and MAPK3 after BRAF inhibition in two different colorectal cancer cell lines, corresponding to Figure 2E (Page 22)

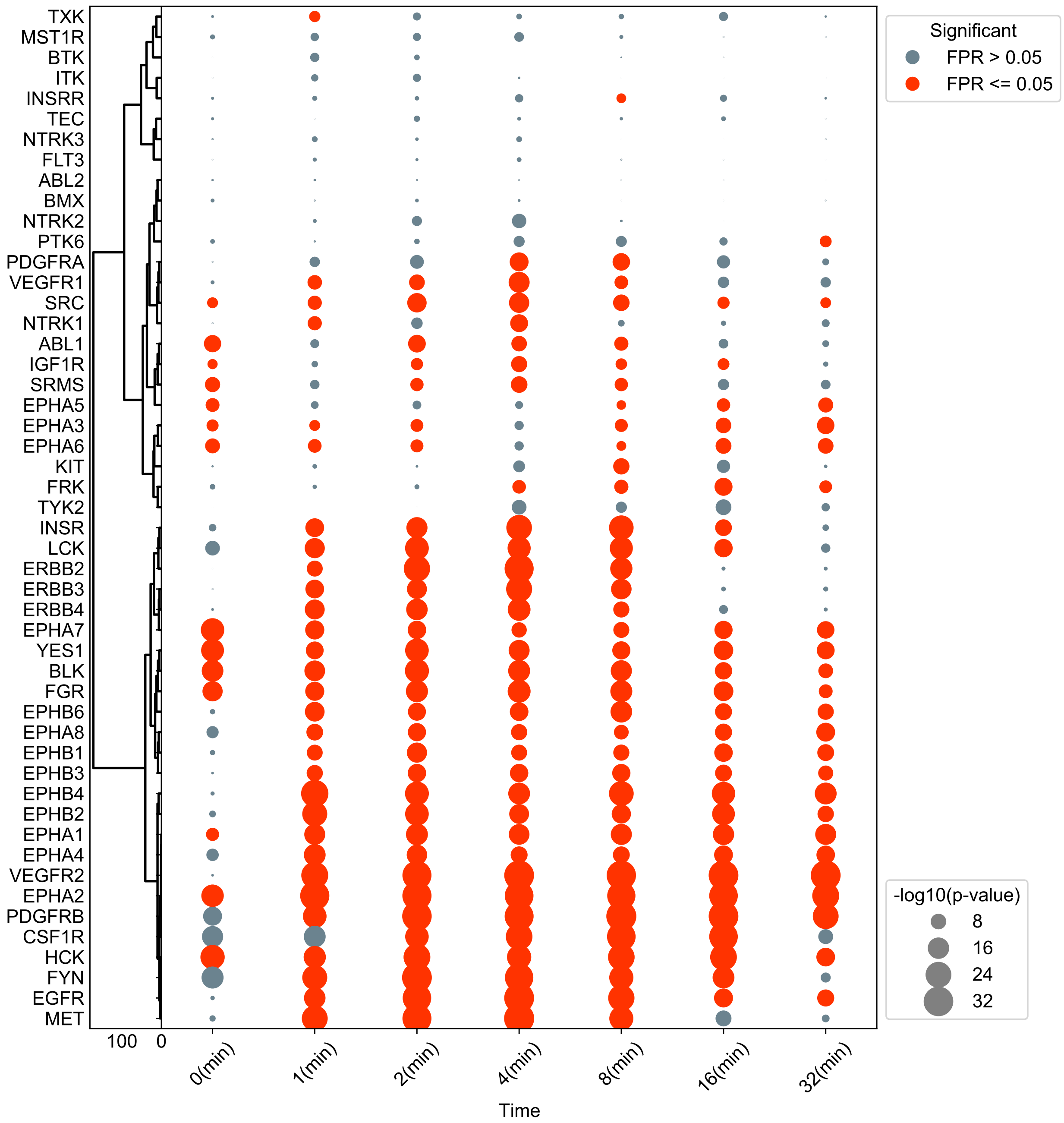

Supplementary Figure 12. EGF stimulation of 184A1 epithelial cells Full KSTAR predictions on EGF stimulation phosphoproteomic data obtained from Wolf-Yadlin et al. [10]. 184A1 epithelial cells were stimulated with EGF and phosphorylation was measured at 0, 1, 2, 4, 8, 16 and 32 minutes. For each condition, sites with abundance ratios greater than 1 were used as evidence, where ratios were relative to the 4 minute timepoint. Kinases were sorted using hierarchical clustering with Ward linkages. Source data are provided as a separate Source Data file with this paper.

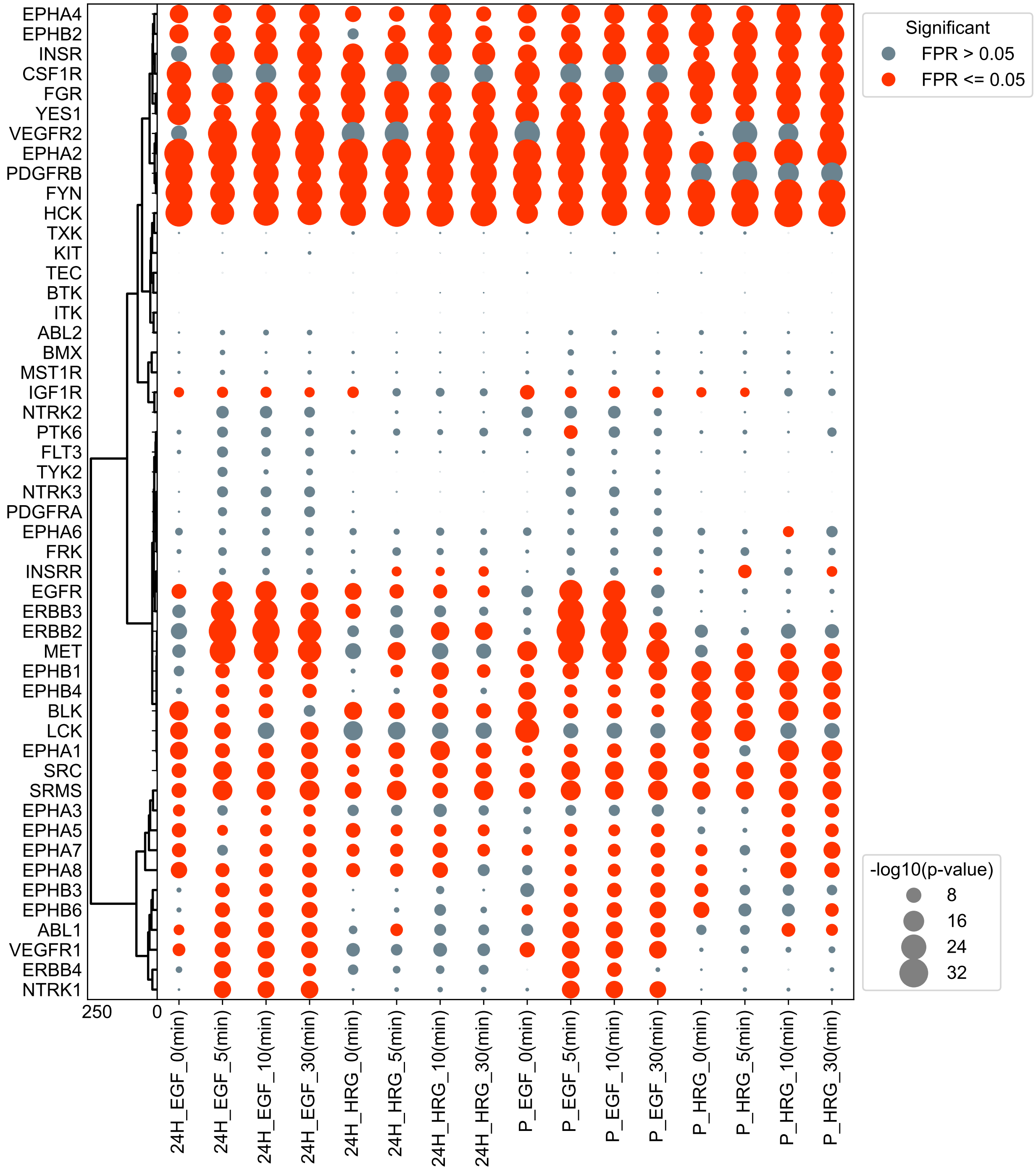

Supplementary Figure 13. EGF/HRG stimulation of 184A1 epithelial cells overexpressing HER2 Full KSTAR predictions corresponding to Figure 2A of the main text, with phosphoproteomic data obtained from Wolf-Yadlin et al. [11]. Epithelial cells expressing normal HER2 levels (Parental, P) or overexpressing HER2 (24H) were stimulated with EGF or HRG and phosphorylation was measured at 0, 5, 10 and 30 minutes. For each condition, sites with abundance ratios greater than 0.8 were used as evidence, where ratios were relative to the Parental, 5 minute EGF condition (P¯EGF¯5(min)). Kinase were sorted using hierarchical clustering with Ward linkages. Parental cell stimulated with EGF corresponds to the same culture conditions measured in Supplementary Figure 12. Source data are provided as a separate Source Data file with this paper.

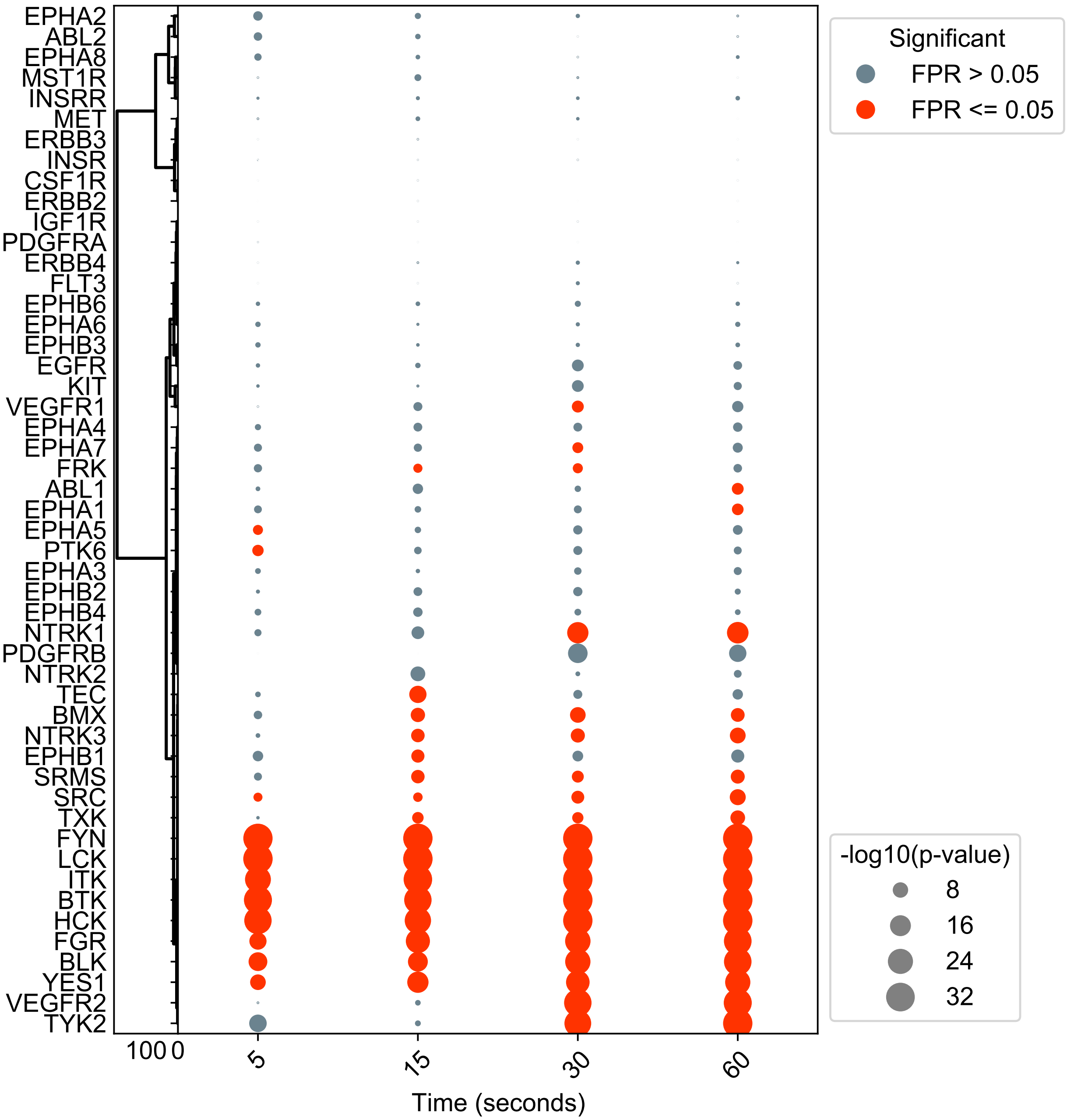

Supplementary Figure 14. TCR activation of Jurkat cells Full KSTAR predictions corresponding to Figure 2B based on data from Chylek et al. [12]. Jurkat cells were stimulated to activate T-cell receptor signaling and phosphorylation was measured at 0, 15, 30, and 60 seconds. For each condition, sites with abundance ratios greater than 0.2 were used as evidence, where ratios were relative to the 0 minute timepoint. Kinases were sorted using hierarchical clustering with Ward linkages. Source data are provided as a separate Source Data file with this paper.

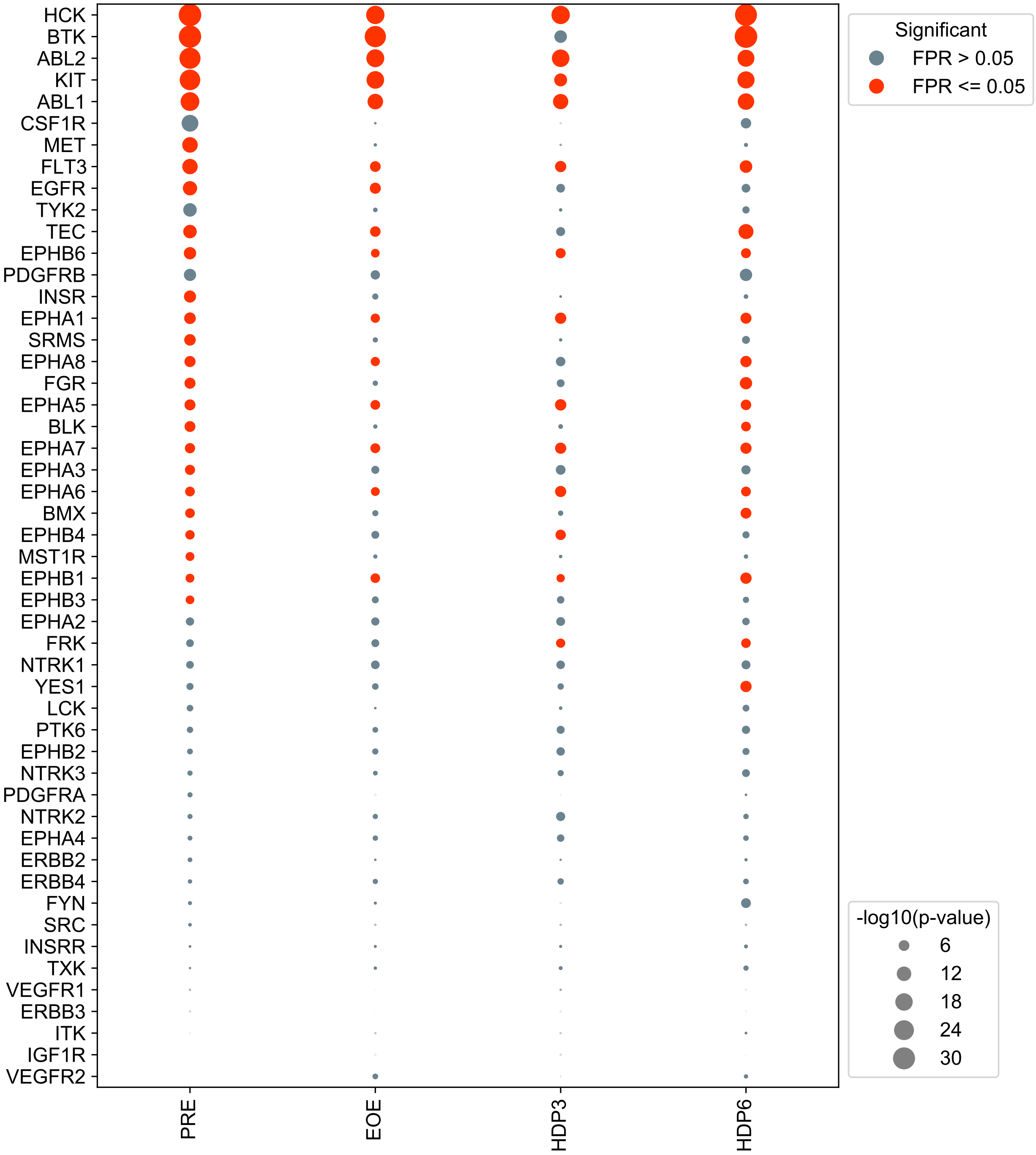

Supplementary Figure 15. BCR-ABL inhibition by dasatanib Full KSTAR predicitions corresponding to Figure 2C of the main text, based on data from Asmussen et al. [13]. K562 chronic myeloid leukemia (CML) cell line, which contains the BCR-ABL fusion protein, was treated with dasatanib, an ABL inhibitor, for 20 minutes prior to drug washout. PRE refers to pre-treatment, EOE refers to the endof treatment, HDP3 refers to 3 hours post drug washout, and HDP6 refers to 6 hours post drug washout. For each condition, sites with abundance ratios greater than 0.5 were used as evidence, where ratios were relative to the 0 minute timepoint. Kinases were sorted using hierarchical clustering with Ward linkages.

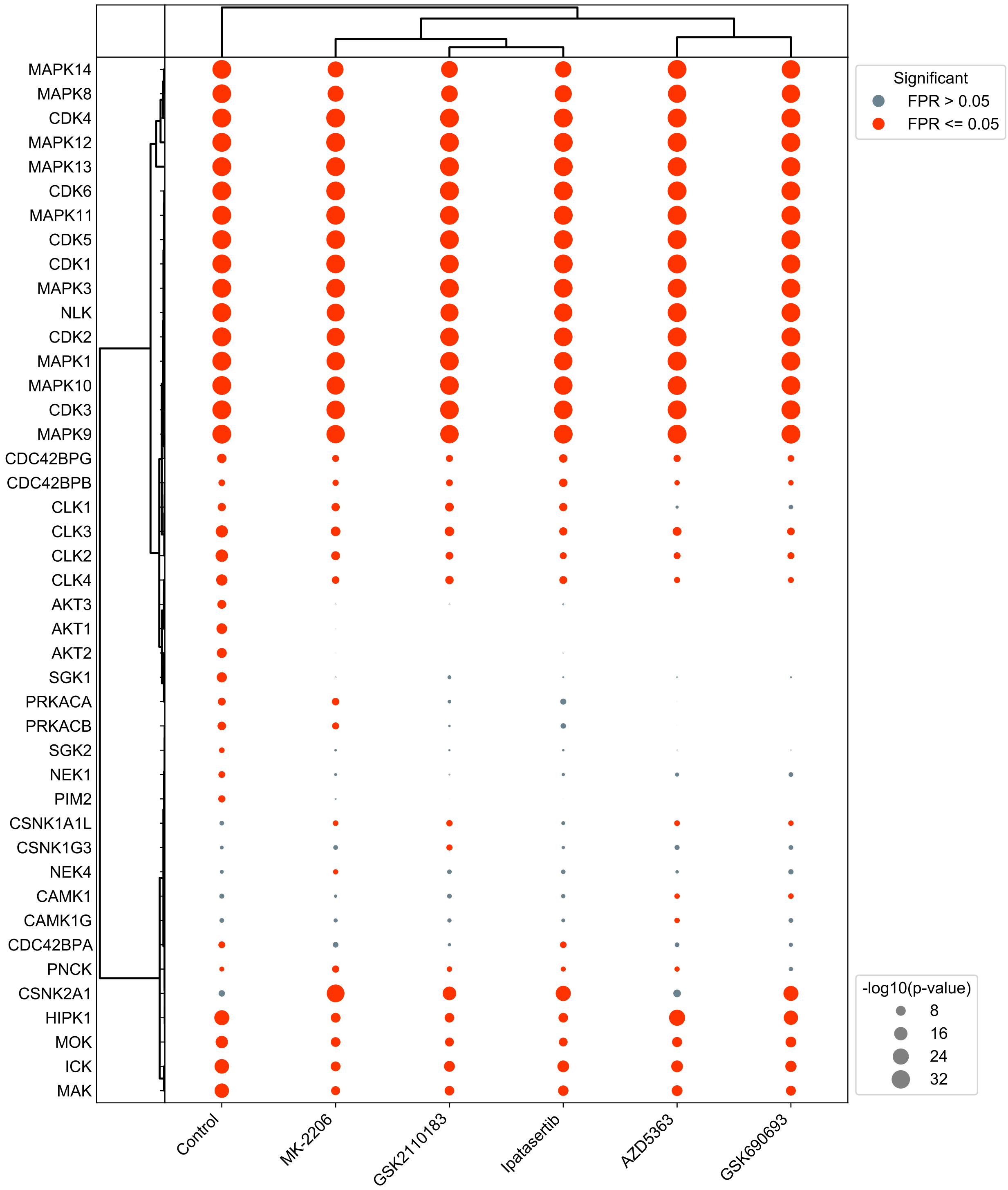

Supplementary Figure 16. AKT Inhibition with breast cancer cells with 5 different AKT inhibitors. Full KSTAR predictions corresponding to Figure 2E of the main text, based on data from Wiechmann et al. [4]. BT-474 breast cancer cells were treated with one of five different inhibitors (GSK2110183, Ipatasertib, AZD5363, GSK690693, MK-2206). All of the inhibitors work by directly binding the ATP binding pocket except for MK-2206, which is an allosteric inhibitor. For each inhibitor condition, sites with abundance ratios greater than or equal to 1 were used as evidence, relative to pre-treatment contol. Kinases and conditions were sorted using hiearchical clustering with Ward linkages. Source data are provided as a separate Source Data file with this paper.

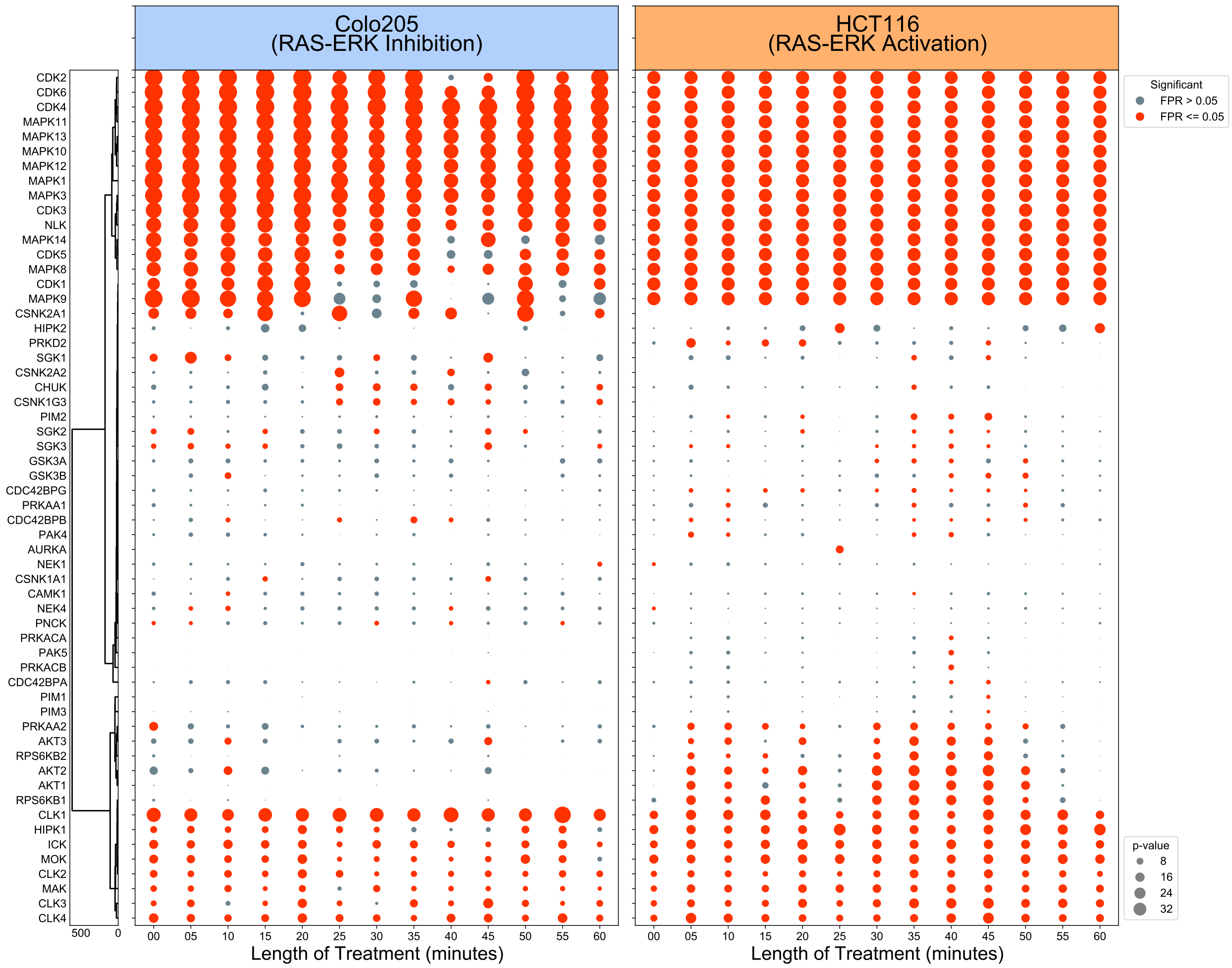

Supplementary Figure 17. Paradoxical MAPK activation after BRAF inhibition Full KSTAR predictions corresponding to Figure 2F of the main text, based on data from Kubiniok et al. [14]. Colorectal cancer cell lines Colo205 ($BRAF^{V600E}$ mutation) or HCT116 (KRAS mutation) were treated with the BRAF inhibitor, vemurafenib, over the course of 60 minutes. In cells containing KRAS mutations, BRAF inhibition leads to the activation of the MAP-ERK pathway rather than inhibiting the pathway, as is the case for cells with a $BRAF^{V600E}$ mutation. For each timepoint/cell line, sites with abundance greater than median observed abundance were used as evidence. Kinases were sorted using hierarchical clustering with Ward linkages. Source data are provided as a separate Source Data file with this paper.

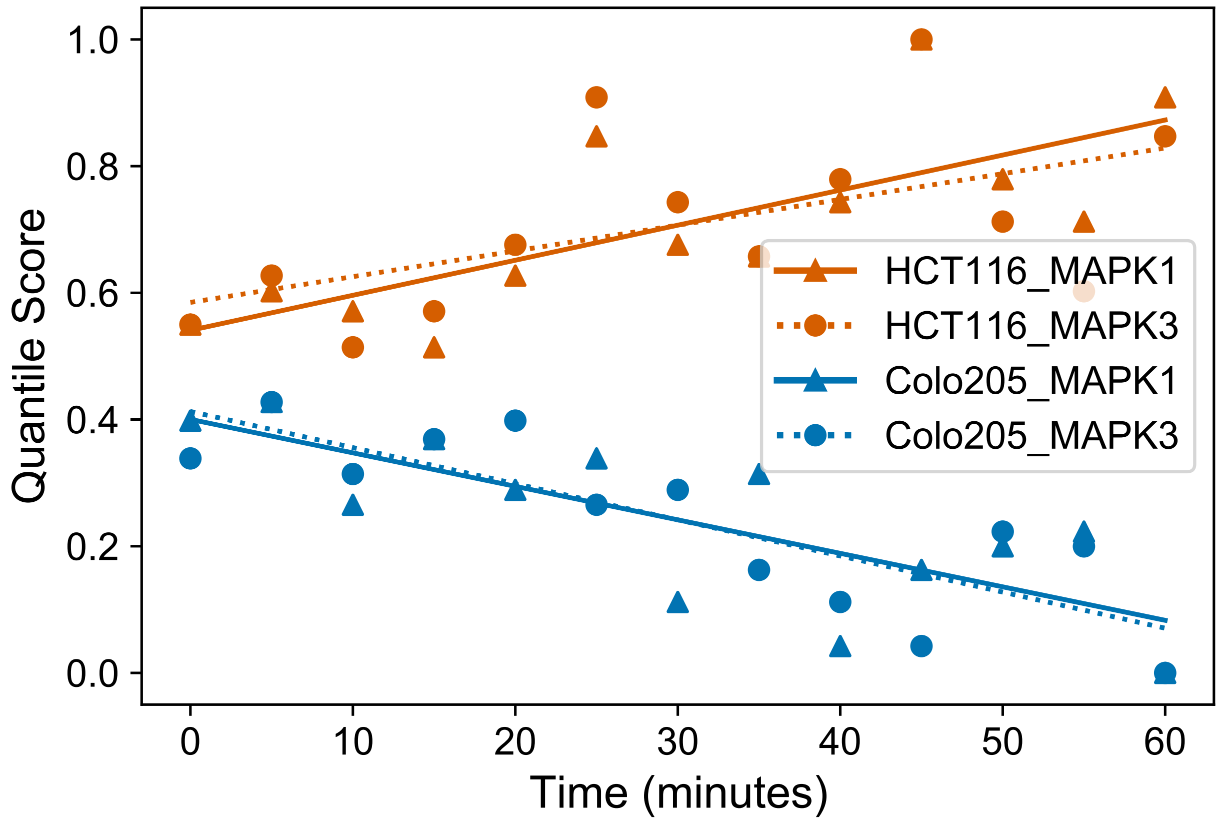

Supplementary Figure 18. MAPK activity response to BRAF inhibition Predicted MAPK activity after performing quantile normalization across conditions on the original KSTAR activities corresponding to Figure 2E of the main text and Supplementary Figure 17. This demonstrates that despite statistical saturation issues in serine/threonine networks where MAPK/CDK activity is often high, the expected trend is observed (increased MAPK activity in HCT116 cells, decreased MAPK activity in Colo205 cells). Source data are provided as a separate Source Data file with this paper.

Supplementary Note 4: Comparing KSTAR to other available activity inference algorithms

Goals

1. Compare the usability and interpretability of various kinase activity inference methods.
2. Compile datasets for use in comprehensive benchmarking of activity inference methods
3. Compare the accuracy of KSTAR to other kinase activity inference methods for both serine/threonine kinases and tyrosine kinases
4. Assess the robustness of algorithms to data loss and the influence of well studied sites on final predictions

Methods

As described in the main text, datasets used in the benchmarking analysis were collected from 16 different publications (10 for ST [4] [15] [16] [17] [18] [19] [20] [21] [22] [23], 7 for Y [13] [10] [11] [12] [24] [25] [21]), described in detail in Supplementary Tables 3 and 4. In total, the benchmarking dataset used contained a total of 51 experimental conditions impacting 38 serine/threonine kinases and 19 tyrosine kinases (Supplementary Figure 20). KSTAR, KSEA [26], PTM-SEA [27], KARP [28], and KEA3 [29] were all used to generate predictions about the most enriched/differentially active kinases across the benchmarking dataset (Supplementary Figure 19). Accuracy was calculated based on P_{hit}, defined as the fraction of times a kinase expected to perturbed was identified as differentially active, either based on kinase rank or significance. In addition, we looked at the total number of kinases with available predictions for each test condition, per algorithm (Supplementary Figures 22 and 23).

In addition to accuracy, we also assessed the impact of losing specific sites from the dataset when generating predictions, either by random removal or targeted removal based on the degree of study bias (Supplementary Figures 25-28). Starting with 5% loss and continuing to 50% loss (at increments of 5%) ,the given percent of sites were removed and predictions were regenerated as normal. We then looked at the change in false discovery rate for each prediction, and how this differed between the random and targeted attack. To quantify the differences between these two curves, we defined two metrics: 1) sensitivity to data loss, which is the area under the random attack curve, and 2) sensitivity to study bias, which is the area between the targeted and random attack curve. Given that we are looking at changes to significance of prediction, only KSTAR, KSEA, and PTM-SEA were assessed (KARP and KEA3 do not provide significance of prediction).

Table of Contents

Supplementary Figure 19 - Chart demonstrating how each algorithm can be interpreted in different scenarios, as well as characteristics of each activity score/rank (Page 24)

Supplementary Figure 20 - Pie charts indicating the distribution of kinases across the benchmarking dataset used here and how the distribution changes when equal weighting of each kinase is applied for P_{hit} calculations. (Page 25)

Supplementary Figure 21 - Full kinase-specific accuracies for all kinases in the benchmarking dataset (Page 26)

Supplementary Figure 22 - Number of kinases with predictions for each condition in the benchmarking datasets (Page 27)

Supplementary Figure 23 - Impact of applying a substrate requirement on the number of kinases with available predictions in KSEA, KARP, and PTM-SEA (Page 28)

Supplementary Figure 24 - Ability of pruned networks to improved performance of other algorithms (Page 29)

Supplementary Figure 25 - Two individual condition examples of the impact of data loss and study bias on activity predictions, as described in Figure 3 in the main text. (Page 30)
Supplementary Figure 26 - Average random and targeted loss curves for individual tyrosine kinases (Page 31)
Supplementary Figure 27 - Average random and targeted loss curves for individual serine/threonine kinases (Page 32)
Supplementary Figure 28 - Average sensitivity to data loss and study bias for individual kinases (Page 33)

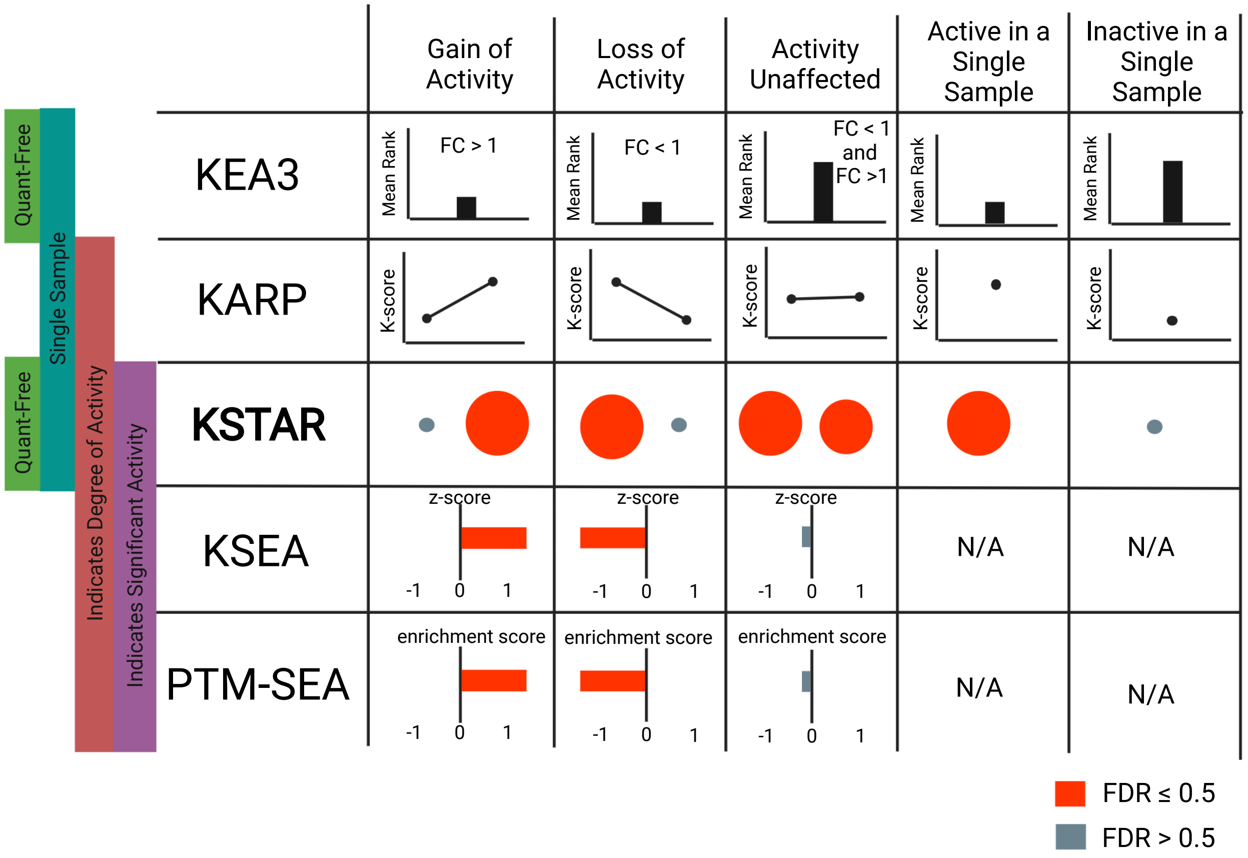

Supplementary Figure 19. Interpreting kinase activity inference methods. Kinase activity algorithms differ both in the type of data that can be used and in the way that their output should be interpreted. KSTAR, KARP, and KEA3 are all algorithms that can be used in single sample settings, where differential abundances are not available and an activity score/rank is generated for each sample. While KARP and KSTAR were explicitly designed for use with single sample experiments, KEA3 was largely designed for differential setttings (all three can be used in differential settings as well). On the other hand, KSEA and PTM-SEA require differential abundances. Of the single sample approaches described here, only KSTAR provides both an indicator of the degree of activity and the associated significance of activity. Finally, only KSTAR and KEA3 do not rely on quantification for prediction. Figure created using Biorender.

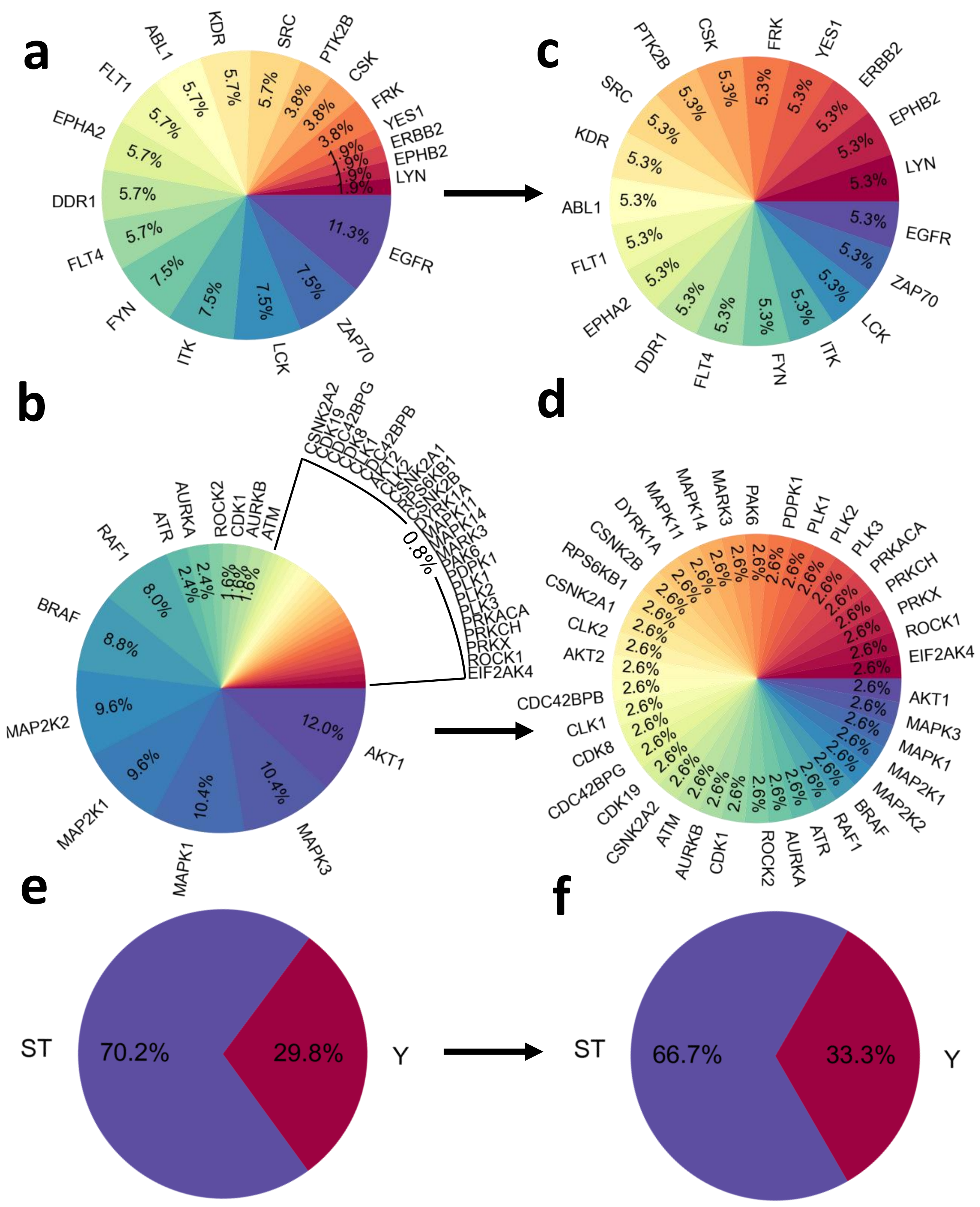

Supplementary Figure 20. Distribution of kinases in benchmarking dataset. A,B) To better understand distribution of kinases across the compiled benchmarking dataset, we looked at the percent influence of each kinase, defined by the fraction of conditions for which a kinase is an expected positive. Tyrosine kinases are shown in A, and serine/threonine kinases are shown in B. Certain kinases are overrepresented, such as AKT1 and MAPK1/3 for serine/threonine and EGFR for tyrosine kinases. (caption continued on next page)

C,D) To avoid certain kinase exhibiting large influence on P_{hit} results, we collapsed each kinase into a single accuracy score ($P_{hit,k}$) and then took the average of each kinase-specific score to obtain the final P_{hit}. The impact of this approach can be seen for tyrosine kinases (C) and serine/threonine kinases (D). **E,F)** Distribution of tyrosine and serine/threonine kinases. We found that there was still an overrepresentation of serine/threonine kinases in the dataset both before (E) and after (F) equal weighting was applied. As a result, we separated serine/threonine and tyrosine kinases for benchmarking purposes.

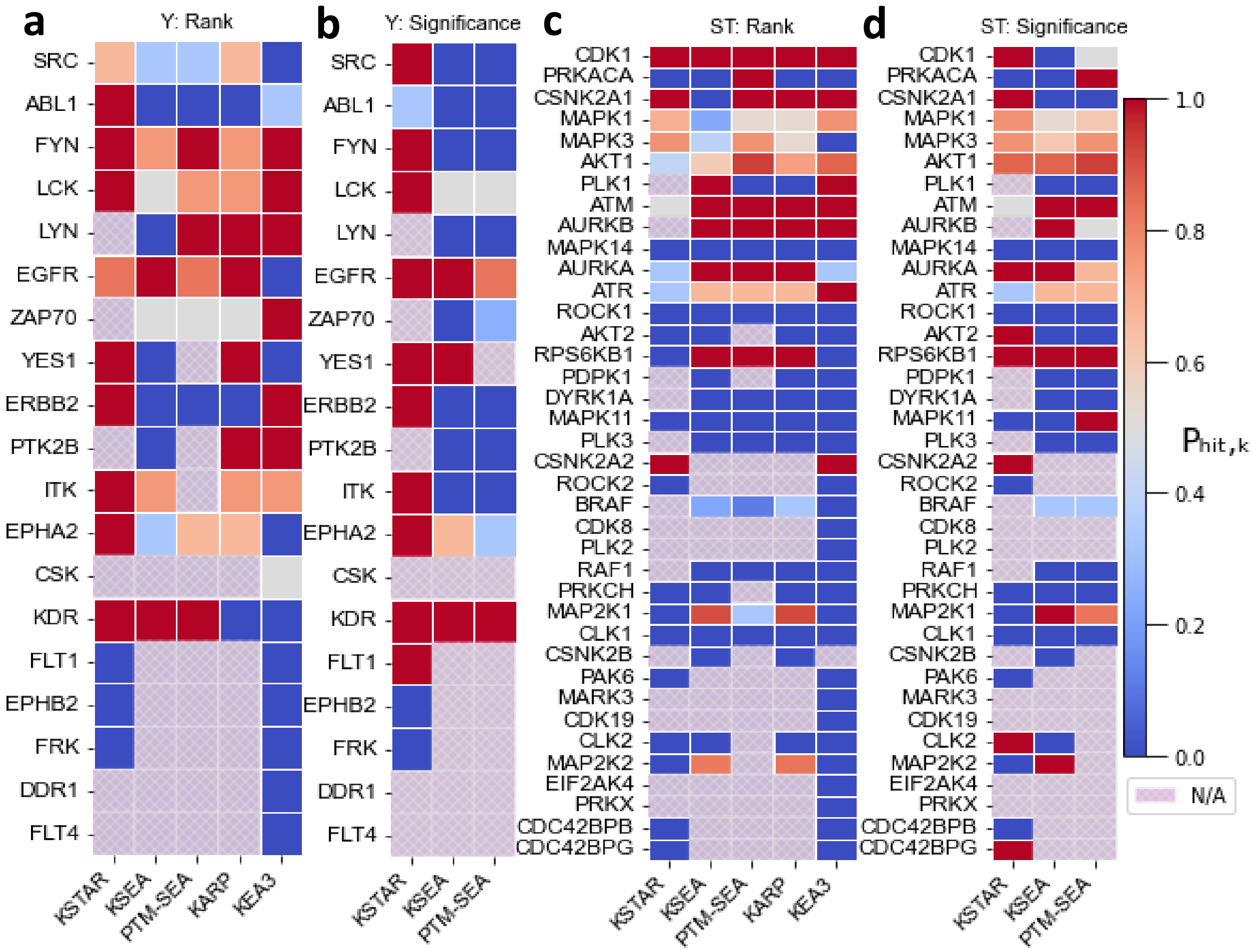

Supplementary Figure 21. Full results obtained from benchmarking analysis. Kinase-specific accuracy scores ($P_{hit,k}$) for all kinases perturbed in the benchmarking dataset. All results seen here were used to calculate the final P_{hit} scores in the barplots from Figure 3A in the main text. Kinase predictions were assessed based on rank (found in the top 10 most differentially active kinases) or significance (differential activity has FDR ≤ 0.05). KARP and KEA3 were only assessed using the rank-based metric. Not all algorithms generated predictions for all kinases, indicated by the light purple squares. **A)** Tyrosine kinase prediction accuracy based on rank. **B)** Tyrosine kinase prediction accuracy based on significance. **C)** Serine/Threonine kinase prediction accuracy based on rank. **D)** Serine/Threonine kinase prediction accuracy based on significance. Source data are provided as a separate Source Data file with this paper.

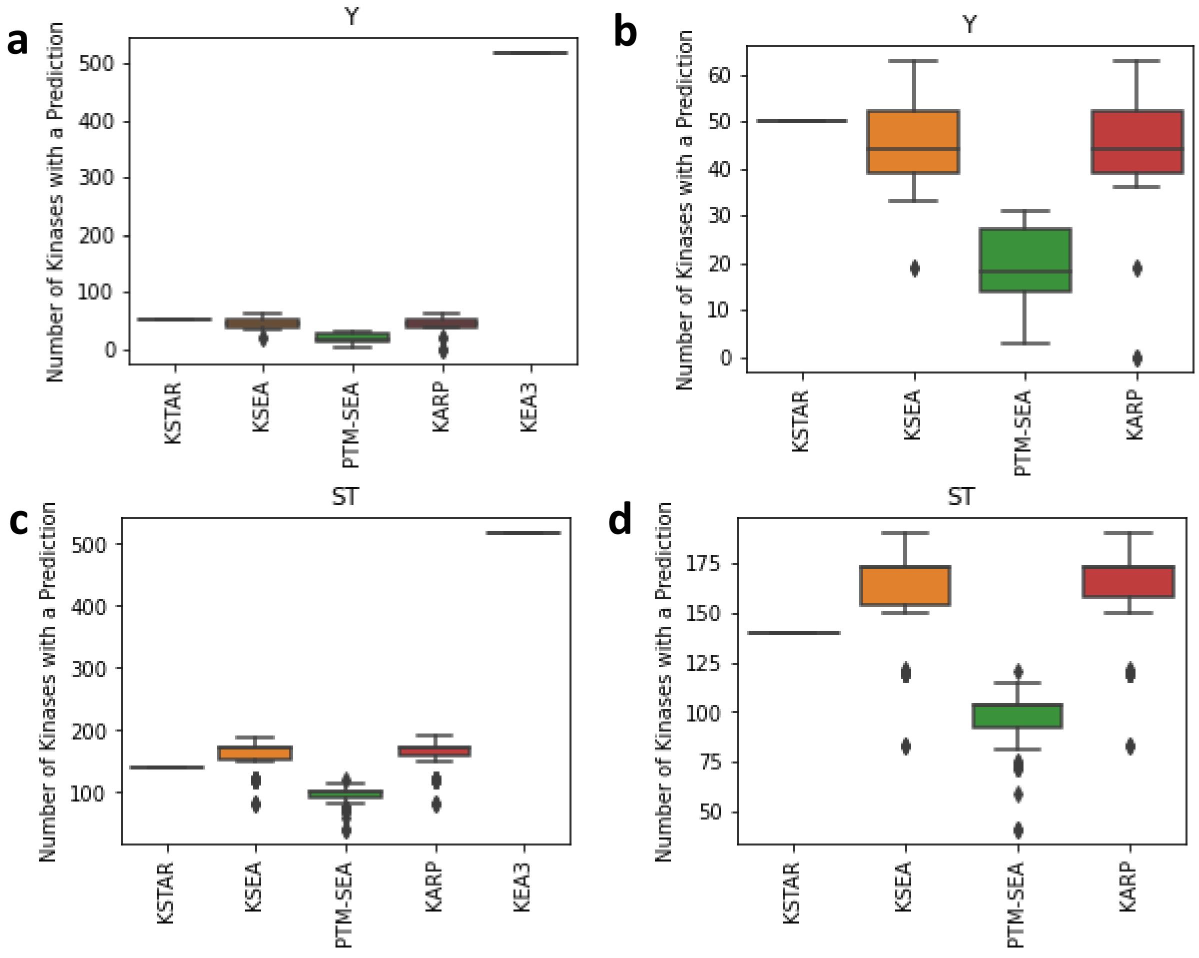

Supplementary Figure 22. Number of Kinases with Predictions Across Different Activity Inference Algorithms. Beyond the accuracy of an algorithm, success of kinase activity inference can also be based on the number of different kinases for which predictions are available. Here, we have displayed boxplots indicating the number of different kinases with a prediction in a single experiment (each point corresponds a single experiment result). Each boxplot indicates the median (center line), 25th and 75th percentiles (box boundaries), 1.5x the IQR of the box edge (whiskers), and any outliers beyond 1.5x IQR (points). If no outliers exist, whiskers indicate maxima or minima. Because KEA3 generates predictions for most existing kinases, regardless of modification type (tyrosine vs. serine/threonine), we have included plots that omit KEA3. KSTAR generates predictions for 50 tyrosine kinases and 140 serine/threonine kinases for all datasets, while KSEA, PTM-SEA, and KARP depend on the specific phosphorylation sites identified in an experiment, where predictions are only generated when at least one known substrate of a kinase is identified. **A)** Number of kinases with predictions for all algorithms based on tyrosine-centric phosphoproteomic datasets described in Supplementary Table 3 (n = 20 biologically independent experiments). **B)** Same plot as A, but omitting KEA3. **C)** Number of kinases with predictions for all algorithms based on serine/threonine centric phosphoproteomic datasets described in Supplementary Table 4 (n = 33 biologically independent experiments). **D)** Same plot as C, but omitting KEA3. Source data are provided as a separate Source Data file with this paper.

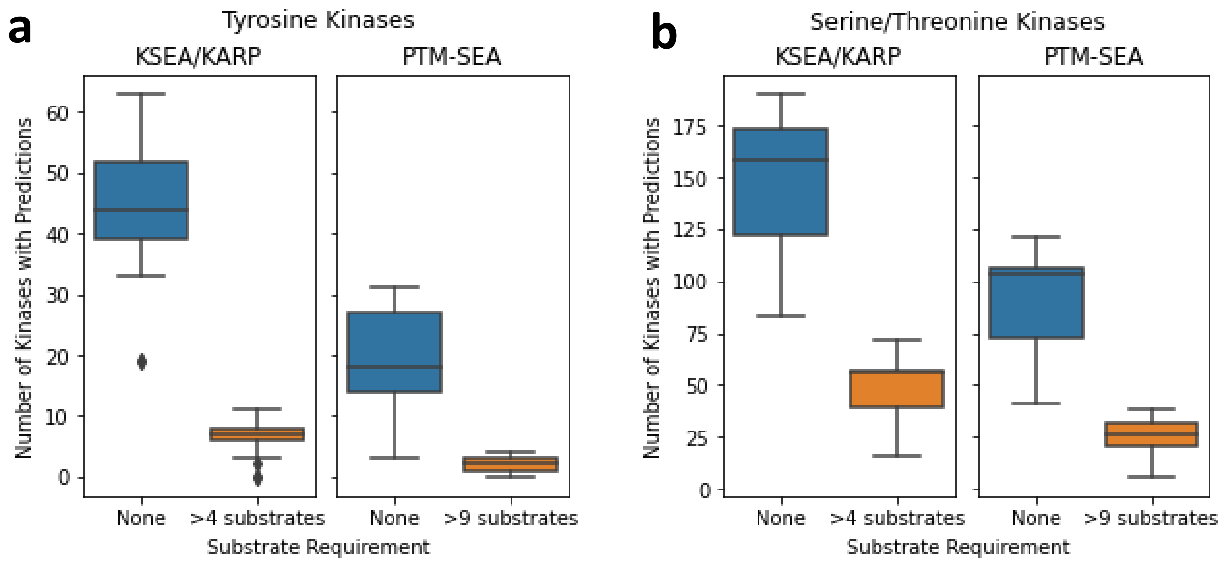

Supplementary Figure 23. Applying substrate requirements significantly reduces overall kinome coverage of many activity inference algorithms. For algorithms relying on kinase-substrate annotation, it is common to restrict analyses to kinases with a set number of substrates. For KSEA, which relies on annotation from PhosphoSitePlus, analysis is restricted to kinases with at least five substrates by default. PTM-SEA, which relies on PTMsigDB, restricts analysis to kinases with at least 10 substrates by default. To determine the impact of these requirements, we asked how many kinases had available predictions for each condition in the benchmarking dataset. We found that, in both cases, the substrate requirement significantly reduced the number of available kinase predictions. For tyrosine kinases, there were several cases where less than 5 kinases had available predictions for a given condition. While these predictions are likely to be more robust as more data is associated with each prediction, the lack of kinome coverage significantly reduces the utility of these algorithms. Further, kinases that tend to be connected to well-studied substrates will be more likely to have available predictions, as well studied sites are more likely to be seen in any given experiment (Supplementary Note 2). For that reason, all accuracy metrics used in benchmarking were calculated using results without this requirement. **A)** Distribution of the number of tyrosine kinases with a prediction from KSEA or PTM-SEA across the benchmarking dataset (described in Supplementary Table 3, n = 20 biologically independent experiments), based on whether a substrate requirement is enforced. Box indicates median (center line), 25th and 75th percentiles (box boundaries), 1.5x the IQR of the box edge (whiskers), and any outliers beyond 1.5x IQR (points). If no outliers exist, whiskers indicate maxima or minima. There is a loss of available kinase predictions for tyrosine kinases when implementing substrate requirements for KSEA or PTM-SEA. While KARP does not explicitly define a substrate requirement, it also relies on PhosphoSitePlus annotations, so would be similarly affected by such a requirement as KSEA. **B)** Distribution of the number of serine/threonine kinases with a prediction from KSEA or PTM-SEA across the benchmarking dataset (described in Supplementary Table 4, n = 33 biologically independent experiments), based on whether a substrate requirement is enforced. Box indicates median (center line), 25th and 75th percentiles (box boundaries), 1.5x the IQR of the box edge (whiskers), and any outliers beyond 1.5x IQR (points). If no outliers exist, whiskers indicate maxima or minima. There is a loss of available kinase predictions for serine/threonine kinases when implementing substrate requirements for KSEA or PTM-SEA. Source data are provided as a separate Source Data file with this paper.

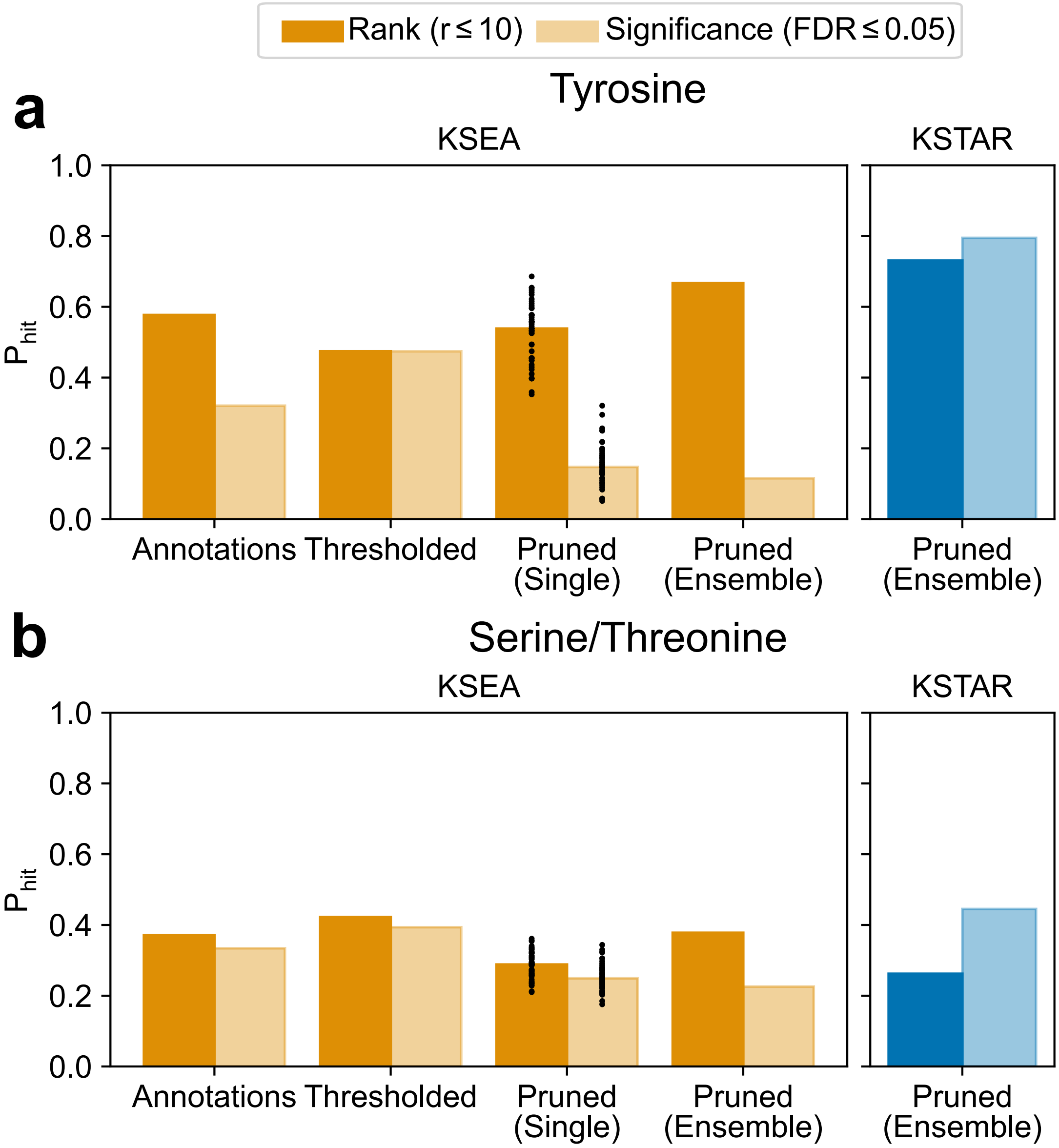

Supplementary Figure 24. Accuracy of KSEA when using different background kinase -substrate networks. To determine whether the general pruning procedure described in this work could also improve the prediction ability of other algorithms, we applied KSEA [26] to the benchmarking dataset using different kinase-substrate networks for **A)** Tyrosine kinases or **B)** serine/threonine kinases. Kinase-substrate information was obtained from three different sources: 1) Annotations: known kinase-substrate interactions stored in PhosphoSitePlus (most commonly used, approach used in the main text), 2) Thresholded: predictions from a NetworKIN graph thresholded with a value of 2 (default in the KSEA web app [30]), and 3) Pruned: KSTAR networks generated through a heuristic prune of the weighted NetworKIN graph (described in Figure 1 of the main text and Supplementary Methods). For the pruned networks, accuracy was either calculated for a single pruned network or based on the median scores/p-values for each kinase across the 50 networks. The former is reported as the median accuracy across the 50 networks, with the accuracy obtained from each single network indicated by the black points. For reference, we also included the accuracy of KSTAR in each plot, which matches Figure 3A of the main text. Source data are provided as a separate Source Data file with this paper.

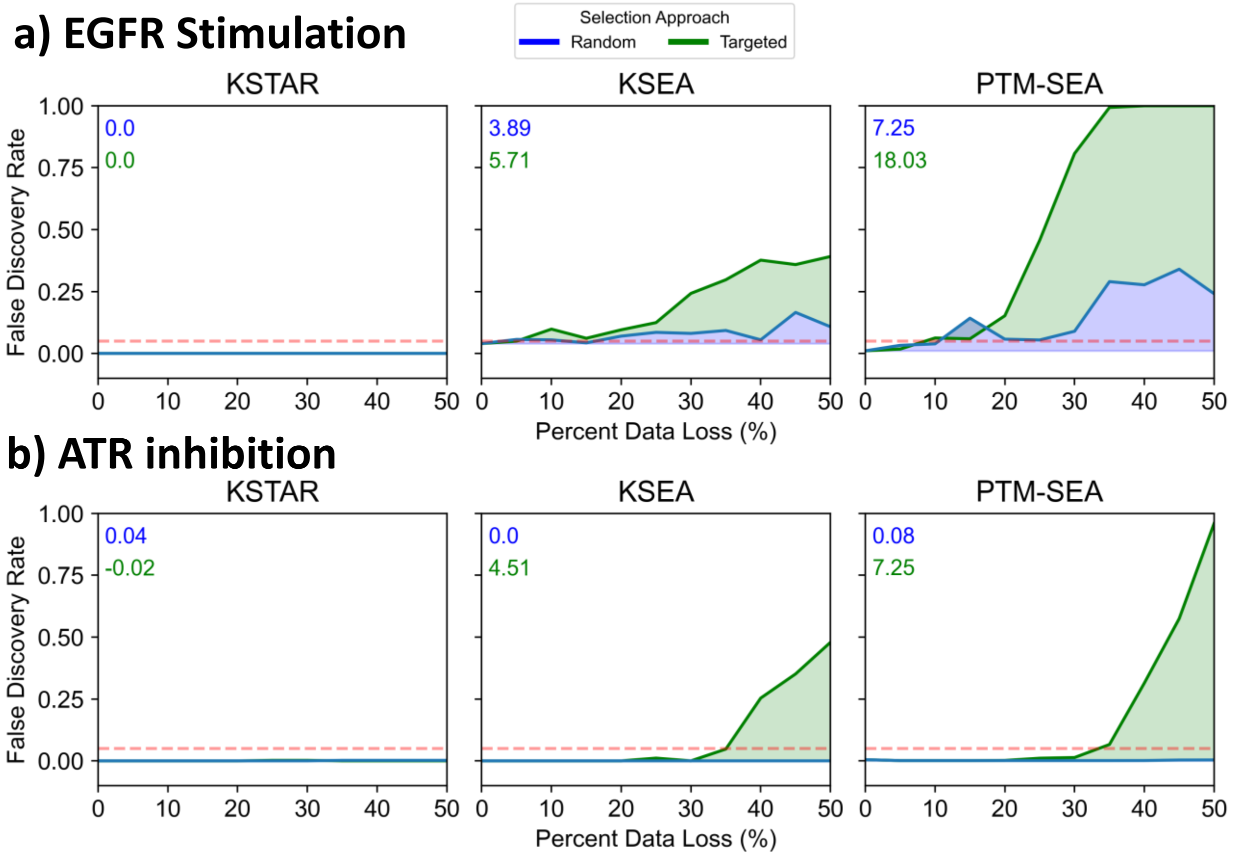

Supplementary Figure 25. Experiment-specific examples of the influence of data Loss and study bias in individual experiments Here, we have provided two specific examples of the loss curves obtained for a single condition via either random or targeted attack. As described in the main text, random loss curves are obtained by randomly removing sites (up to 50% of them, done in increments of 5%) and obtaining the average false discovery rate obtained by each algorithm at each data loss amount. Targeted loss curves are obtained in a similar fashion, except that more well studied sites (defined by the number of compendia they are found in) are removed first. **A)** EGF stimulation condition from Wolf-Yadlin et al. [10], which is experiment #4 from Supplementary Table 3, focused on predicted EGFR activity increase after EGF stimulation. **B)** ATR inhibition condition from Beli et al. [23], which is experiment #51 from Supplementary Table 4, focused on predicted ATR activity decrease. Source data are provided as a separate Source Data file with this paper.

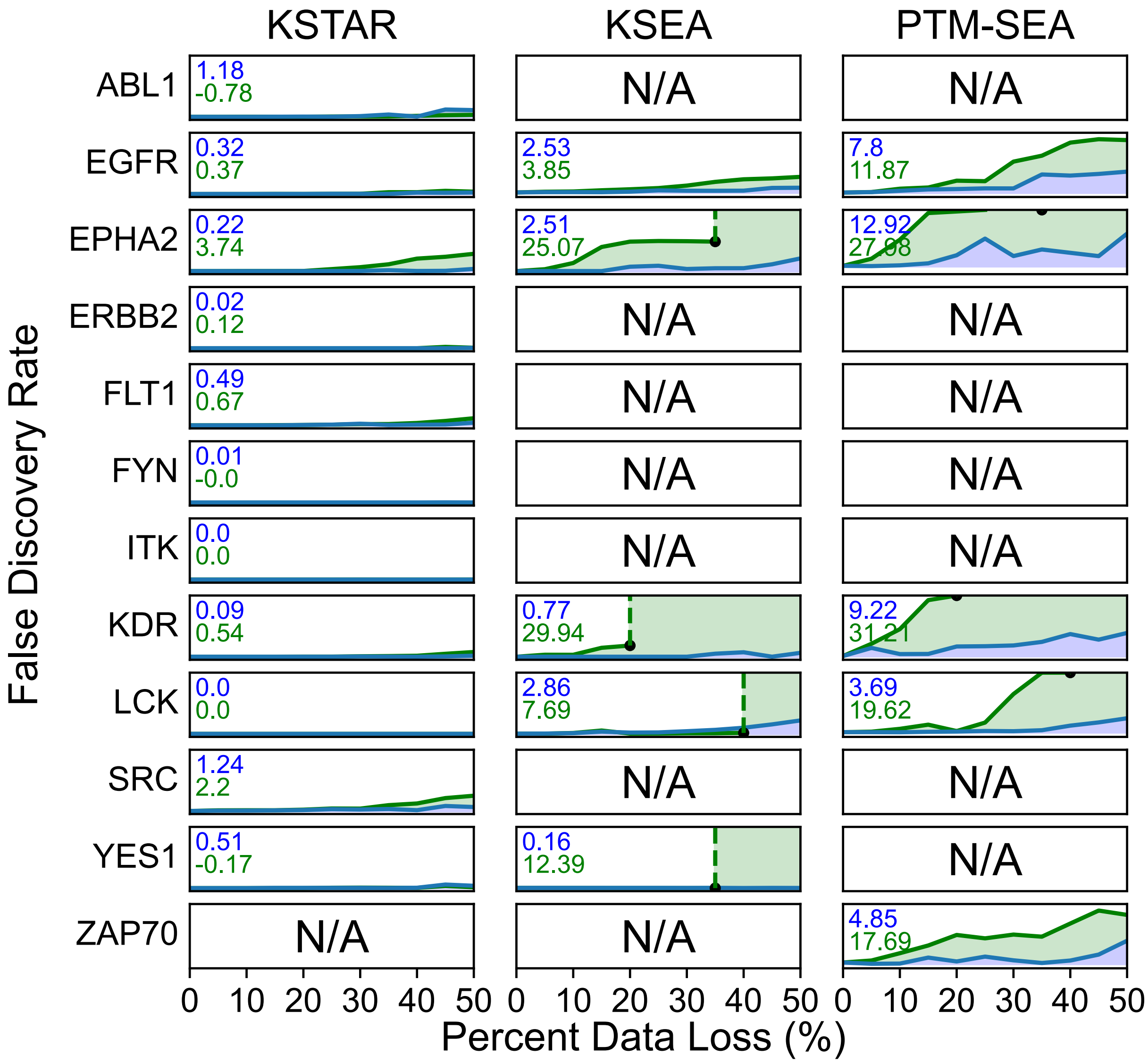

Supplementary Figure 26. Tyrosine kinase predictions lose significance with increasing data loss. The average random and targeted loss curves for each tyrosine kinase tested in the data loss experiment (corresponds to Figure 4 of the main text). Rows correspond to a specific kinase, columns correspond to a specific algorithm. The x-axis indicates increasing data loss from 0 to 50%, and the y-axis indicates the false discovery rate of the predicted activity for the kinase of interest, ranging from 0 to 1. The average sensitivity to data loss (blue) and study bias (green) are displayed in the upper left of each plot (these values correspond to heatmap in Supplementary Figure 21). In cases where the curve stops before reaching 50% data loss (indicated by a black point at the stop point), this occurs because there is no longer any available substrates in the data that can be used for prediction in either PhosphoSitePlus [1] (for KSEA) or PTMsigDB [27](for PTM-SEA). Source data are provided as a separate Source Data file with this paper.

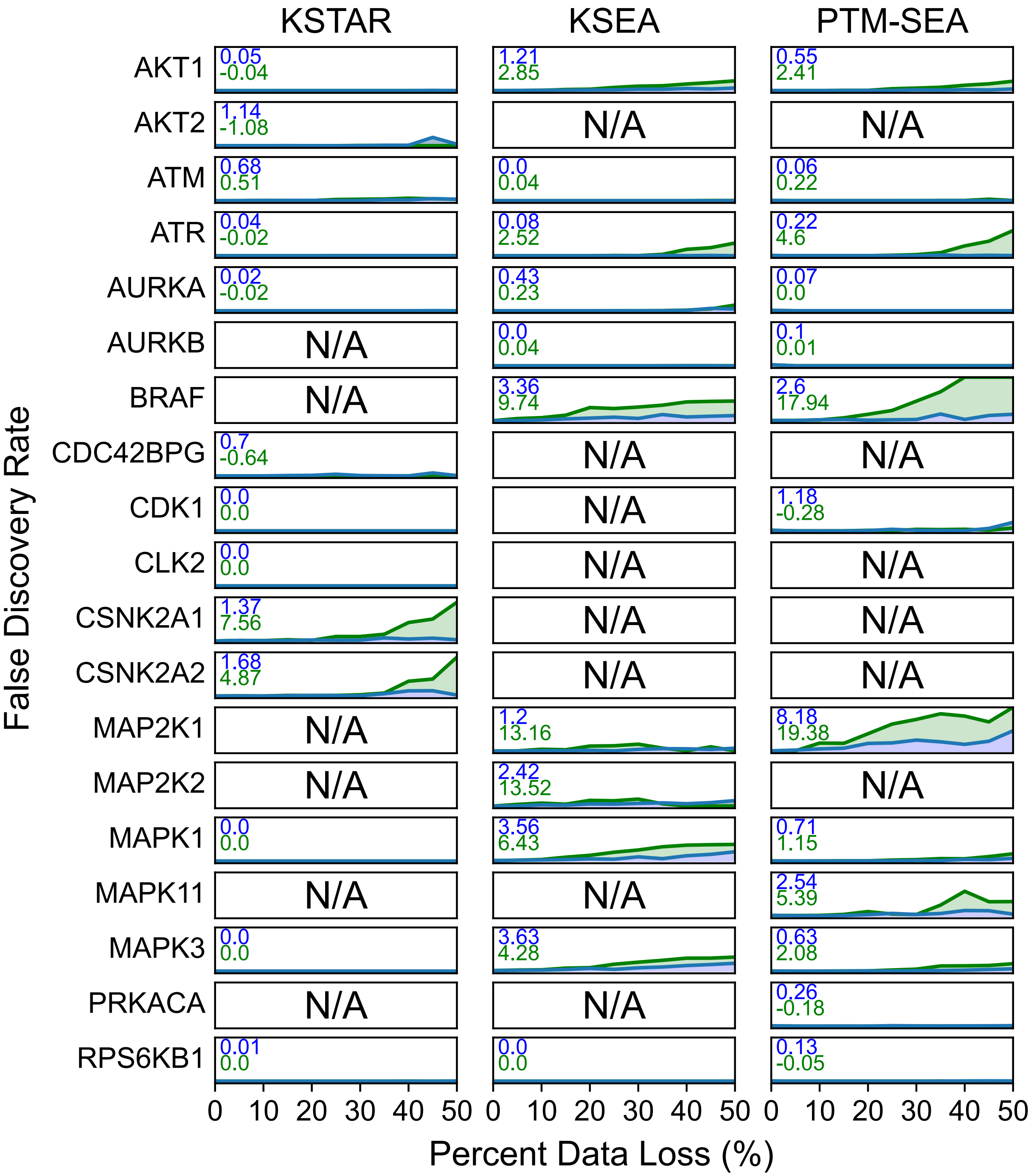

Supplementary Figure 27. Serine/threonine kinase predictions lose significance with increasing data loss. The average random or targeted loss curves for each serine/threonine kinase tested in the data loss experiment (corresponds to Figure 4 of the main text). Rows correspond to a specific kinase, columns correspond to a specific algorithm. The x-axis of each plot indicates increasing data loss from 0-50%, and the y-axis indicates the false discovery rate of the predicted activity for the kinase of interest, ranging from 0 to 1. The average sensitivity to data loss (blue) and study bias (green) are displayed in the upper left of each plot (these values correspond to heatmap in Supplementary Figure 21). In cases where the curve stops before reaching 50% data loss, this occurs because there is no longer any available substrates in the data that can be used for prediction in either PhosphoSitePlus [1] (for KSEA) or PTMsigDB [27](for PTM-SEA). Source data are provided as a separate Source Data file with this paper.

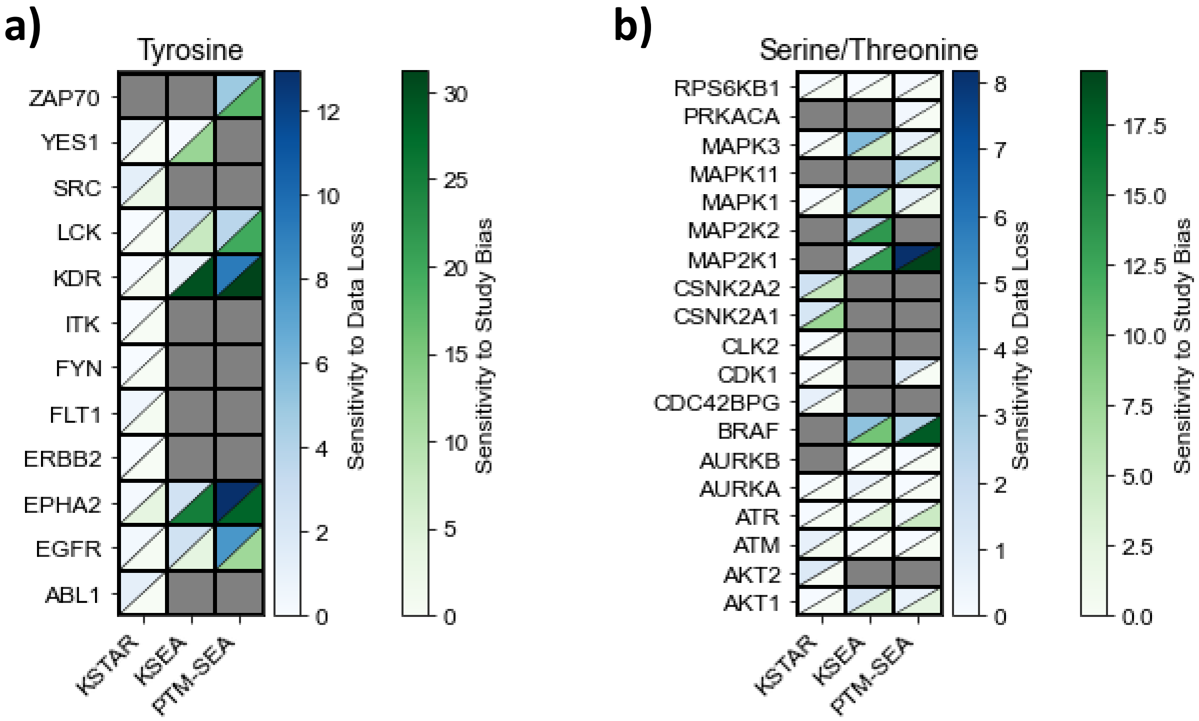

Supplementary Figure 28. Kinase-specific sensitivity to data loss and study bias for different activity inference methods. Heatmaps indicating the average sensitivity to data loss and study bias for individual kinases, rather than the whole benchmarking dataset. As described in the main text, ”sensitivity to data loss” is defined as the area under the random attack curve, while ”sensitivity to study bias” is defined as the difference in the area under the curve between the targeted and random attack. Cells in the heatmap that are gray indicate that predictions were either not available or were not found to be significant in the full dataset. We found that in addition to KSTAR being generally less sensitive to both random and targeted attacks, certain kinase predictions tended to suffer more from targeted attack (removal of well-studied sites), such as KDR (also known as VEGFR2), EPHA2, and MAP2K1 .**A)** Tyrosine kinases **B)** Serine/Threonine Kinases. Source data are provided as a separate Source Data file with this paper.

Supplementary Note 5: Robustness analysis comparing NSCLC and CML cell lines from independent experiments

Goal

Apply KSTAR and KEA3 [29] predictions to multiple different datasets from different labs profiling non small cell lung carcinoma (NSCLC) and/or chronic myeloid leukemia (CML) cell lines to determine if predictions can identify tissue similarities between datasets even when the identified sites differ. These results correspond to Figure 5 in the body of the paper.

Methods

A total of 11 datasets from 7 studies and 5 labs were obtained from the original publications (described in Supplementary Table 5) [31] [32] [33] [34] [35] [3] [13]. For each dataset, only data corresponding to an untreated cell line was utilized. To generate predictions for each dataset, any site identified in the experiment, regardless of quantification, was utilized as evidence. In KSTAR, the phophorylated sites were inputted into the algorithm to produce activity scores and false positive rates. In KEA3, the gene names associated with each phosphorylated site were extracted and inputted into the KEA3 API in python to generate integrated mean ranks, where low mean ranks indicate that the kinase was found to be one of the more enriched kinases in many different protein-protein interaction databases. Spearman rank correlation was used (either with KSTAR activity scores or KEA3 mean ranks) to assess the similarity of predictions across datasets.

Table of Contents

Supplementary Figure 29 Full KSTAR activity predictions for each of the 11 datasets and the top ranking kinases in each dataset (Page 3)
Supplementary Figure 30 Correlation of KEA3 mean ranks across each of the 11 datasets (Page 4)

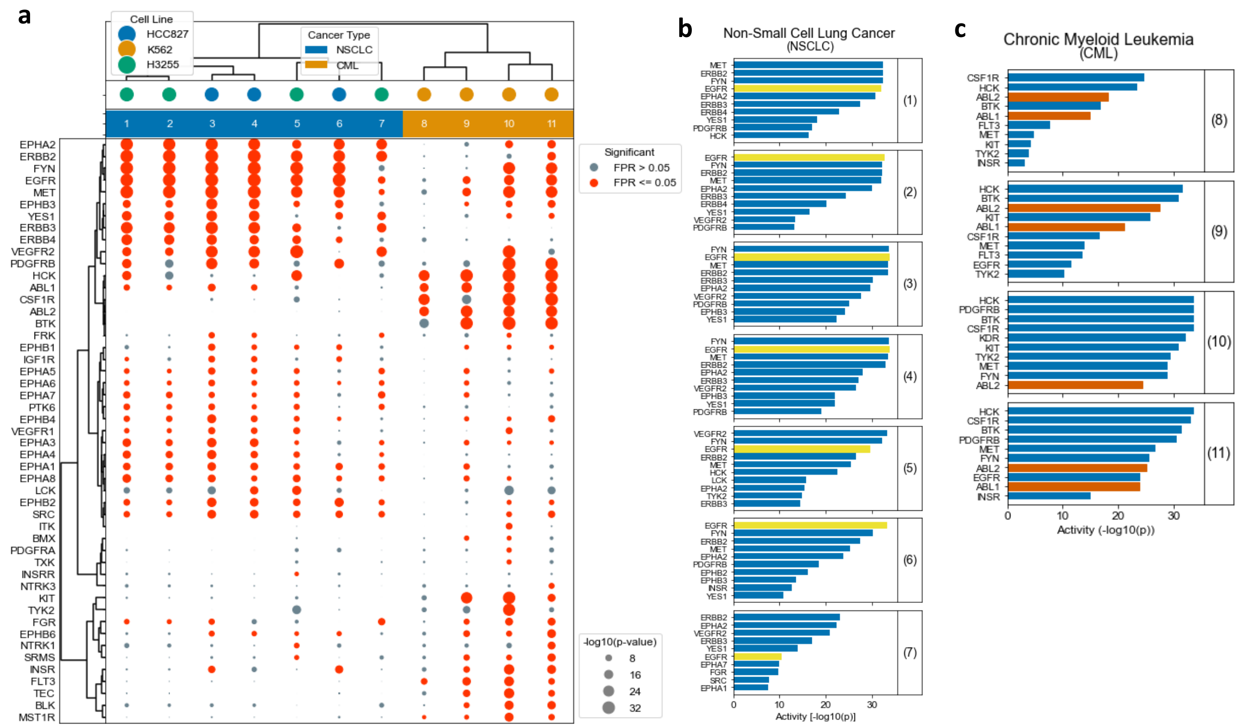

Supplementary Figure 29. Full KSTAR predictions on NSCLC and CML cell lines The full KSTAR predictions across all datasets used in robustness analysis. **A)** Full KSTAR dotplot including activity predictions for all kinases with at least one sample that has significant activity ($FPR \leq 0.05$). The size of each dot indicates the degree of predicted activity, with red dots indicating significant activity. Context bars above the plot indicate the cancer type and dataset number. Context dots above the plot indicate the specific cell line measured. Kinases and samples are sorted using hierarchical clustering with ward linkages. **B)** Top 10 most active kinases in each non small cell lung cancer (NSCLC) dataset, as predicted by KSTAR. Size of the bar indicates the degree of activity. As both H3255 and HCC827 contain activating EGFR mutations, we have the EGFR activity bar in yellow. **C)** Top 10 most active kinases in each chronic myeloid leukemia (CML) dataset, as predicted by KSTAR. ABL1 and ABL2 are highlighted in red, as the K562 cell lines contains the BCR-ABL fusion protein. Source data are provided as a separate Source Data file with this paper.

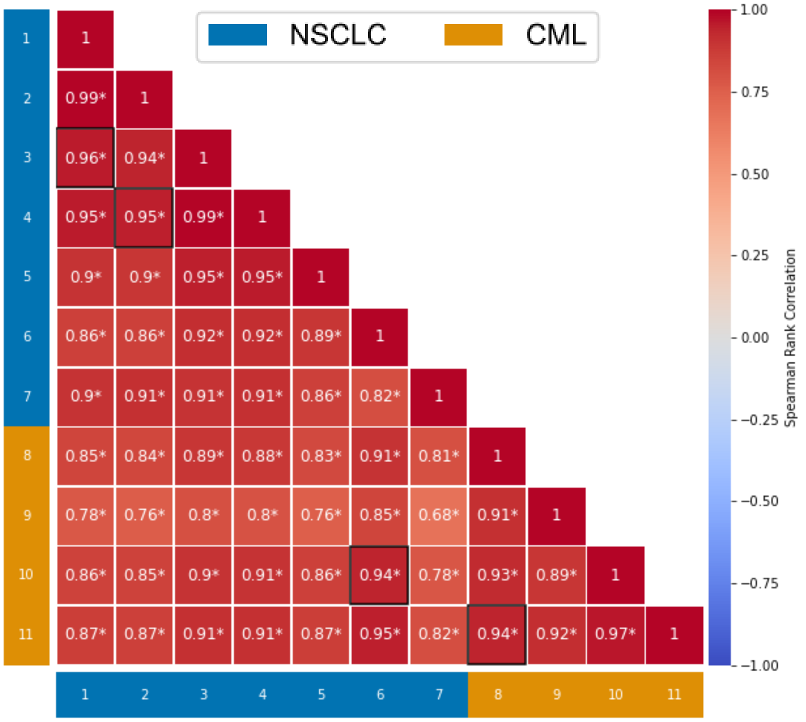

Supplementary Figure 30. KEA3 predictions on NSCLC and CML cell lines Heatmap indicating the pairwise correlation between KEA3 rankings obtained from each dataset used in the robustness analysis, based on Spearman correlation coefficient. KEA3 ranks are calculated by reducing the predictions to only include the 50 tyrosine kinases predicted in KSTAR, and then ranked according to KEA3 mean rank. Datasets are sorted according to the same sorting used for the KSTAR heatmap in Figure 5 and in Supplementary Figure 29. Heat cells surrounded by a black box indicate that datasets were obtained from the same study. Overall, this demonstrates that KEA3 was unable to clearly differentiate between the tissues across datasets, with most predictions exhibiting high correlation between each other, regardless of tissue type. Source data are provided as a separate Source Data file with this paper.

Supplementary Note 6: Full analysis results of breast cancer phosphoproteomic datasets

Goal

1. Assess the ability of kinase activity inference approaches to predict treatment response in the clinic, particularly in the context of HER2 activity in breast cancer
2. Compare the efficacy of KSTAR, KEA3, and KSEA in the clinical setting

Methods

In this supplement, we have provided the KSTAR predictions for all kinase and samples discussed in Figure 6 of the main text, focused on predicting kinase activity in breast tumor biopsies. For the microscaled biopsies of HER2+ patients [36], we have included multiple different dotplots to seperate between the pre- and post-treatment data and provide the predictions for serine/threonine kinases in pre-treatment samples (to illustrate that serine/threonine kinase activity profiles do not appear to differentiate between responders and nonresponders).

In addition, where relevant, we have compared our KSTAR results to the results obtained by either KSEA (Supplementary Figure 32) or KEA3 (Supplementary Figures 32, 38, and 40). We found that KSEA results from the tumor biopsies tended to be nonrobust and difficult to apply in this setting (due to low numbers of known substrates and reliance on quantification), so we did not continue use of KSEA after application to the CPTAC dataset [37]. To make KEA3 more directly comparable to KSTAR results, the list of kinases with predictions from KEA3 was reduced to include only kinases that also have predictions in KSTAR. As all predictions are based on the abundance of phosphorylated tyrosines (except in Supplementary Figure 36), this also had the effect of removing serine/threonine kinases from KEA3 predictions, which are not relevant to the actual input data. The KEA3 rankings displayed throughout this supplement are then obtained based on the KEA3 mean ranks, where the 10th ranked kinase had the 10th smallest mean rank across the 50 tyrosine kinases with predictions in KSTAR.

Table of Contents

Supplementary Figure 31 - Full KSTAR predictions on CPTAC phosphoproteomic analysis of 77 Breast cancer patients, corresponding to Figure 4A (Page 2)

Supplementary Figure 32 - KSTAR, KSEA, and KEA3 predictions in the CPTAC breast cancer dataset for ERBB2/HER2 and their correlation with HER2 status. Corresponds to Figure 4A (Page 3)Supplementary Figure 33 - Full KSTAR predictions on phosphoproteomic analysis of PDX models ofbreast cancer, corresponding to Figure 4B (Page 4)

Supplementary Figure 34 Full KSTAR tyrosine kinase activity predictions for microscaled biopsies ofHER2+ patients, corresponding to Figure 4C (Page 5)

Supplementary Figure 35 Pre-treatment KSTAR tyrosine kinase activity predicitons for microscaledbiopsies of HER2+ patients, corresponding to Figure 4C (Page 6)

Supplementary Figure 36 Pre-treatement KSTAR serine/threonine kinase activity predictions for microscaled biopsies of HER2+ patients, corresponding to Figure 4C (Page 7)

Supplementary Figure 37 - Patient-specific KSTAR activity predictions for ERBB2/EGFR in non-pathologically complete responders from microscaled biopsies, corresponding to Figure 4C (Page 8)Supplementary Figure 38 - Patient-specific KEA3 rankings for ERBB2/EGFR in non-pathologicallycomplete responders from microscaled biopsies, corresponding to Figure 4C (Page 9)

Supplementary Figure 39 - Patient-specific KSTAR activity predictions for ERBB2/EGFR in pathologically complete responders from microscaled biopsies, corresponding to Figure 4C (Page 10)

Supplementary Figure 40 - Patient-specific KEA3 rankings for ERBB2/EGFR in pathologically complete responders from microscaled biopsies, corresponding to Figure 4C (Page 11)

Supplementary Figure 41 - Ranking of ERBB2 in microscaled biopsies and its relation to HER2 status, based on either KSTAR activity scores or KEA3. Corresponds to Figure 4C (Page 12)

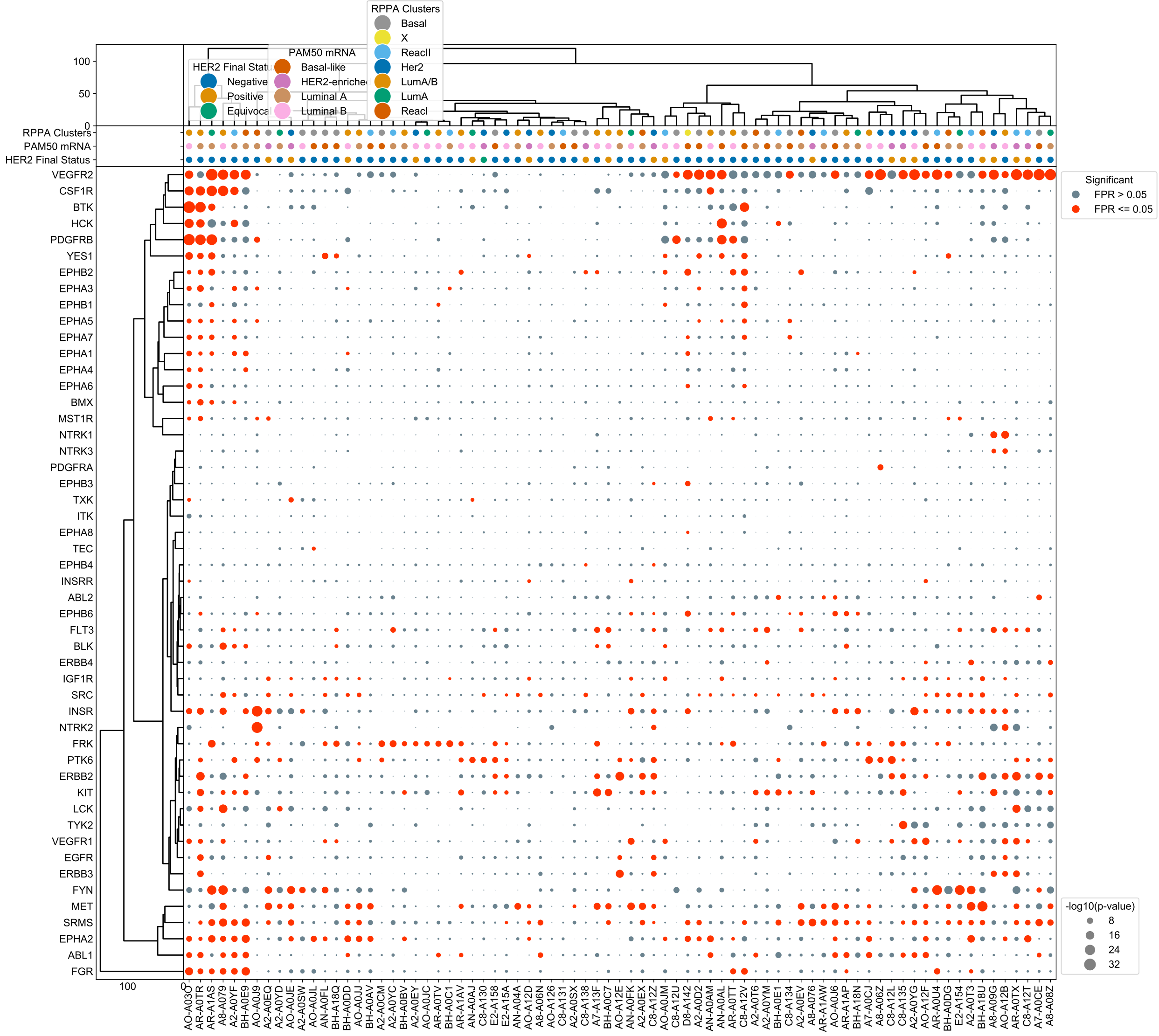

Supplementary Figure 31. Full KSTAR predictions on CPTAC phosphoproteomic analysis of 77 Breast cancer patients Dotplot containing all kinase activity predictions for the CPTAC phosphoproteomic dataset [37], corresponding to Figure 6A. Dot size corresponds the activity score (-log10(p)), and dots are colored based on if the observed activity score has a false positive rate below 0.05. Kinases and samples were sorted using hierarchical clustering with ward linkage. Important clinical attributes of each patient can be found above the main dotplot, including the HER2 status and classification based on either mRNA or protein expression data. Source data are provided as a separate Source Data file with this paper.

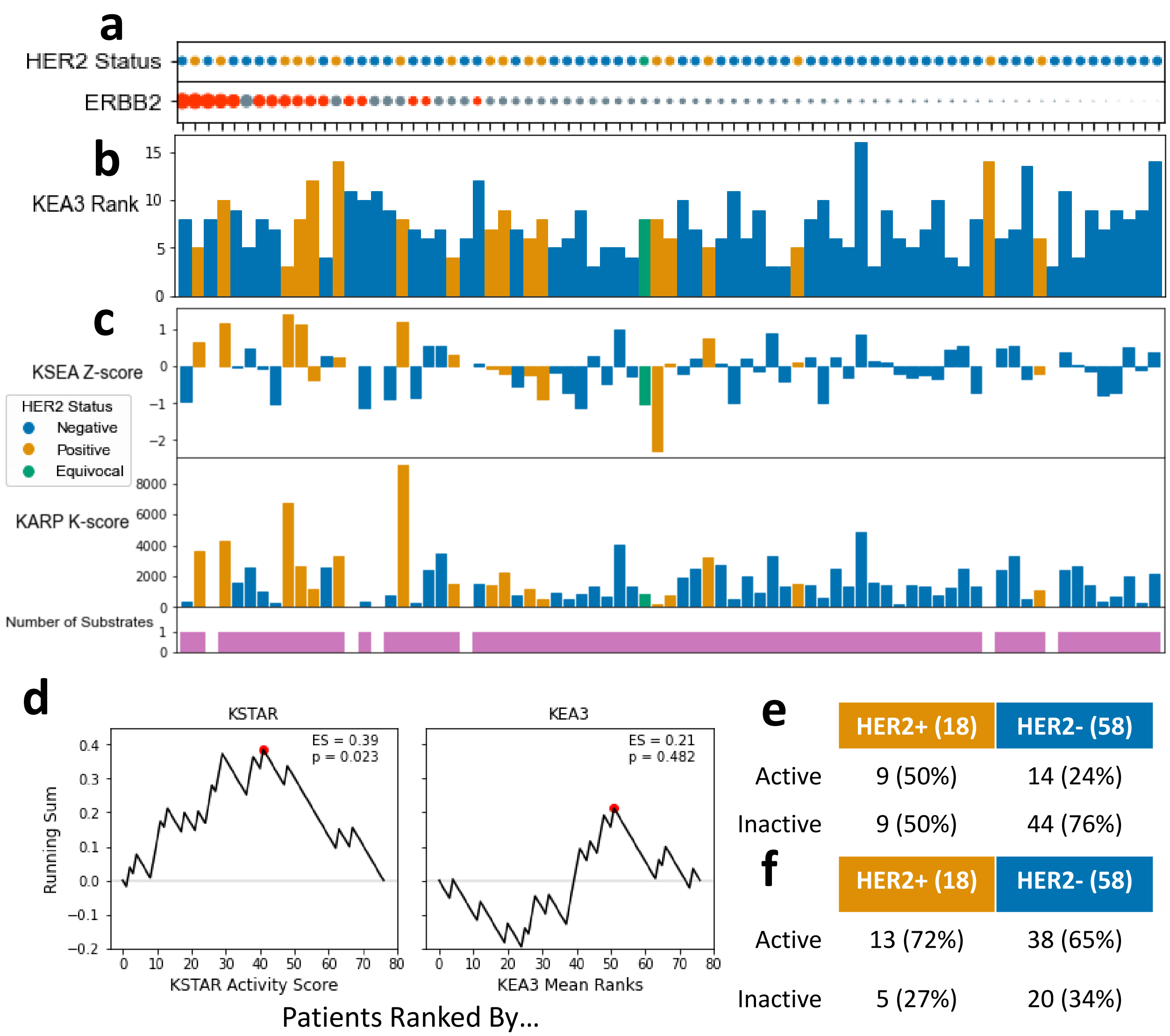

Supplementary Figure 32. Comparing Kinase Activity Inference Approaches Relationship with HER2 Status Predicted HER2 activity using different kinase activity inference algorithms (KSTAR, KSEA [26], KEA3 [29]), with patient samples sorted based on KSTAR predicted activity. HER2 status is indicated using the color of the bars/dots depending on plot type. **A)** KSTAR predictions of ERBB2/HER2 activity, where each where dotsize indicates degree of activity and red samples are significant ($FPR <= 0.05$). This plot is identical to Figure 6A. **B)** KEA3 ERBB2/HER2 ranking, relative to the 50 kinases that also have predictions in KSTAR. So, for a sample where the rank is 10, ERBB2/HER2 is the 10th most enriched kinase out of 50 other tyrosine kinases. **C)** KSEA z-scores and number of identified ERBB2 substrates for each CPTAC patient sample. In patients with at least one ERBB2/HER2 substrate, z-score enrichment was calculated with measured log2 transformed abundances. Positive z-scores indicate high ERBB2 activity and negative z-scores indicate low ERBB2 activity, relative to a pooled sample. Empty bars correspond to patients with no identified substrates, as indicated by PhosphoSitePlus [1]. All patient samples with KSEA predictions for HER2/ERBB2 relied on a single site as evidence, LDHA Y10. Significant z-scores are indicated by '*' (FDR <= 0.05). **D)** GSEA-style running sum to test if KSTAR HER2/ERBB2 activity or KEA3 HER2/ERBB2 mean ranks significantly correlates with HER2 status. To calculate the running sum, samples were sorted according to predicted ERBB2 activity obtained from KSTAR or KEA3, with the most ERBB2 active samples at the top of the list. We then moved through the list, adding to the running sum if the sample was HER2+ and subtracting from the running sum if the sample was HER2-. (Caption continued on next page)

The final enrichment score is equal to the maximum deviation from0 obtained in the running sum. Statistical significance was then calculated using a null distributionconsisting of ES scores obtained when the sample list was sorted randomly. Only KSTAR ERBB2 activitypredictions were found to be significantly correlated with HER2 status. **E)** HER2 status predictions for KSTAR, with active ERBB2/HER2 defined by $score \leq 1e-3$. Same table as in Figure 4A. **F)** HER2 status predictions for KEA3, defining active ERBB2/HER2 by an ERBB2 rank of 8 or higher. This cutoff was determined by identifying the rank cutoff with the best overall F1 score for KEA3. Source data are provided as a separate Source Data file with this paper.

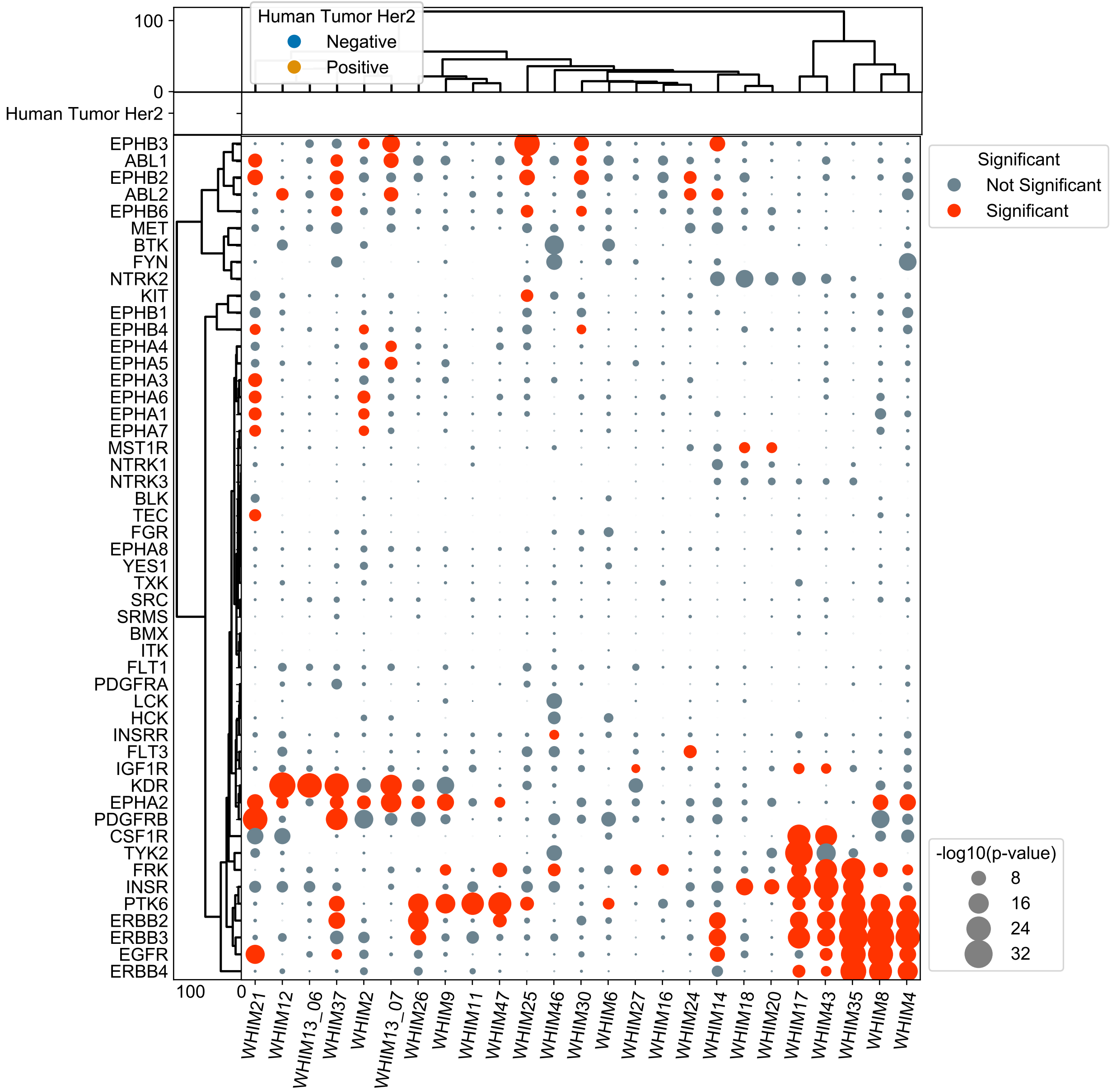

Supplementary Figure 33. Full KSTAR predictions on PDX Dataset Dotplot containing all tyrosine kinase activity predictions for the PDX dataset [8], corresponding to Figure 6B. Dot size corresponds the activity score (-log10(p)), and dots are colored based on if the observed activity score has a false positive rate below 0.05. Kinases and samples were sorted using hierarchical clustering with ward linkage. HER2 status of each patient is indicated above the dotplot. 3 of the 25 PDX samples were HER2+, the rest of the samples were HER2-. Source data are provided as a separate Source Data file with this paper.

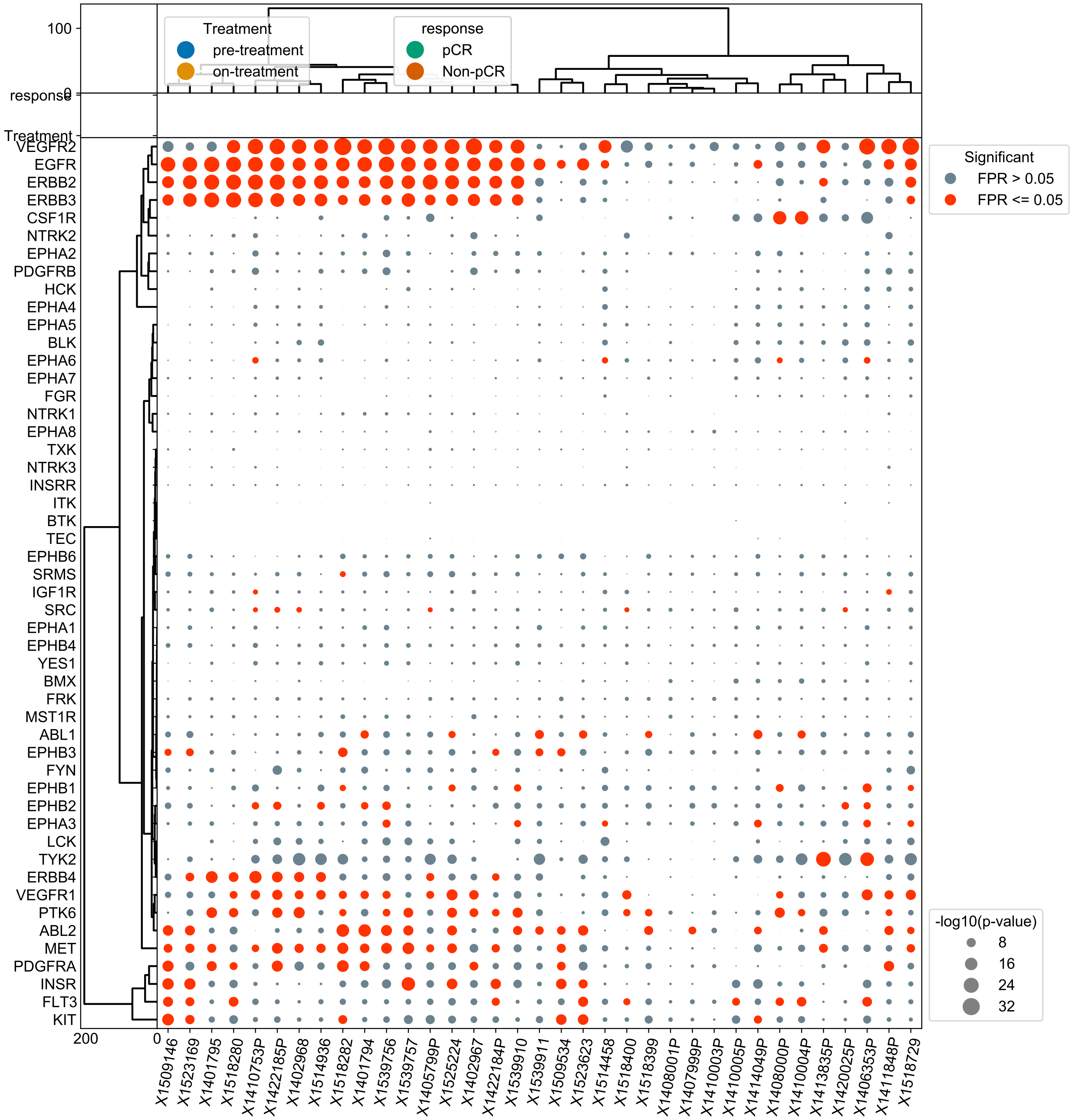

Supplementary Figure 34. Full KSTAR tyrosine kinase activity predictions on microscaled biopsies Full dotplot containing all tyrosine kinase activity predictions on Microscaled Biopsies of HER2+ patients for both pre- and post-treatment [36]. Corresponds to Figure 6C. Patients were treated with a combination of chemotherapy and HER2 targeted therapy, and post-treatment biopsies were taken 48-72 hours following the beginning of treatment. Dot size corresponds to the activity score (-log10(p)), and dots are colored based on if the observed activity score has a false positive rate below 0.05. Kinases and samples were sorted using hierarchical clustering with ward linkage. Treatment status (pre- vs. post-treatment) and treatment response (responder vs. non-responder) of each sample are indicated with the context dots above the dotplot. Source data are provided as a separate Source Data file with this paper.

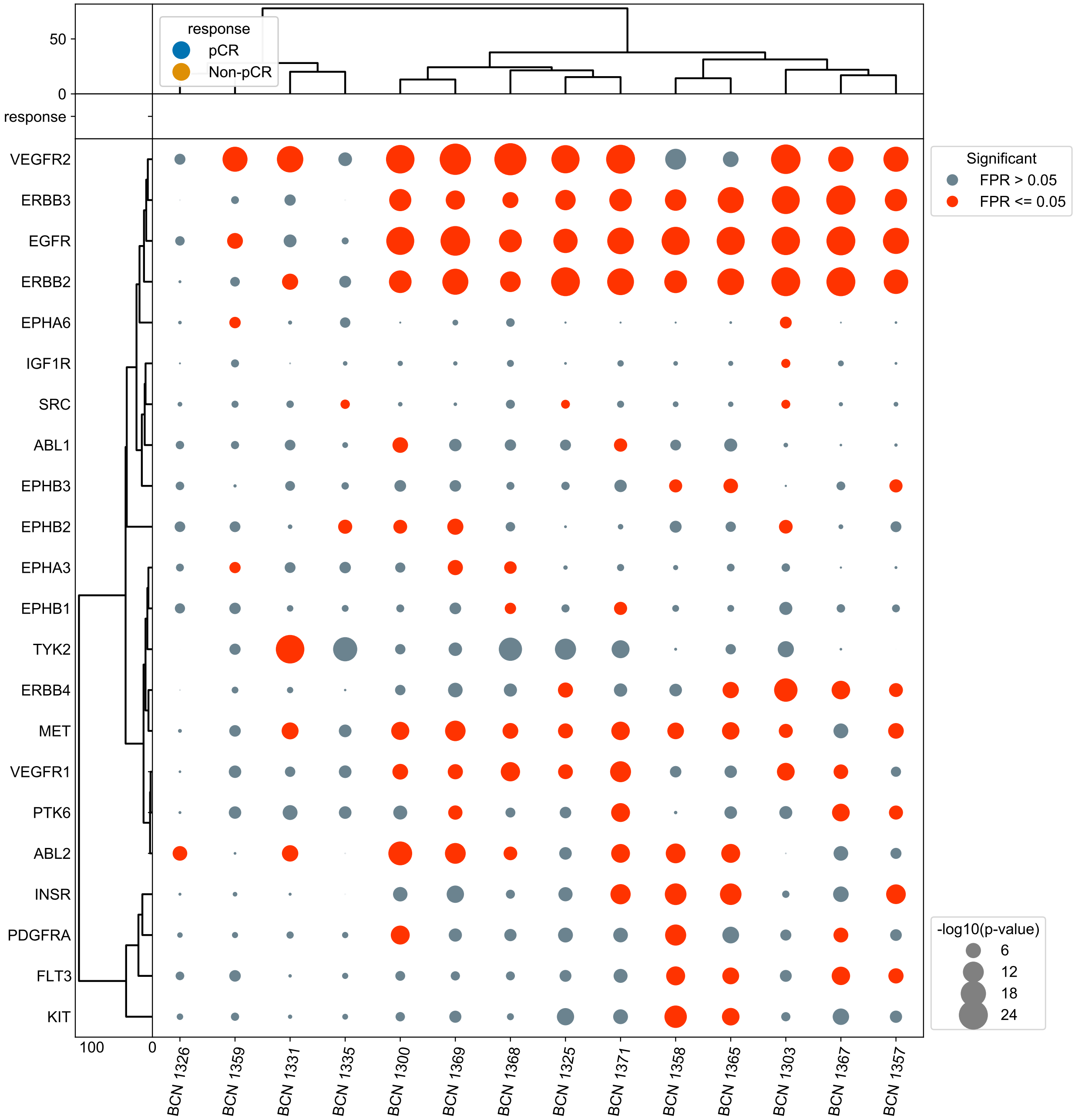

Supplementary Figure 35. KSTAR tyrosine kinase activity predictions on microscaled biopsies prior to treatment Full dotplot containing all tyrosine kinase activity predictions on microscaled biopsies of HER2+ patients for only pre-treatment from Satpathy et al. [36]. Corresponds to Figure 6C. Dot size corresponds to the activity score (-log10(p)), and dots are colored based on if the observed activity score has a false positive rate below 0.05. Kinases and samples were sorted using hierarchical clustering with ward linkage. Treatment response to combination therapy of chemotherapy and anti-HER2 therapy (responder/pCR vs. non-responder/non-pCR) of each sample are indicated with the context dots above the dotplot. Source data are provided as a separate Source Data file with this paper.

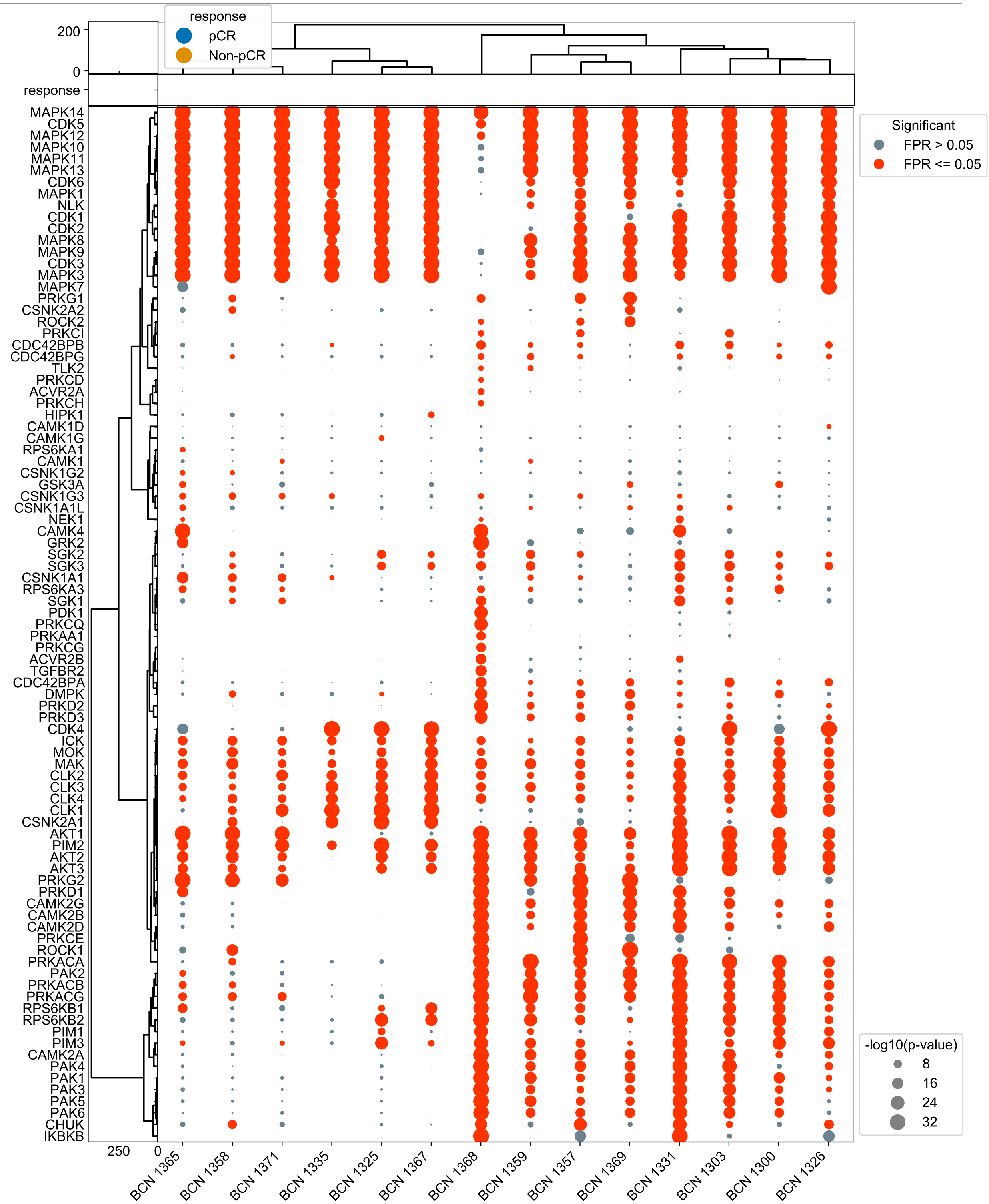

Supplementary Figure 36. KSTAR serine/threonine kinase activity predictions on microscaled biopsies prior to treatment Full dotplot containing all serine/threonine kinase activity predictions (for which at least one sample patient had on microscaled biopsies of HER2+ patients for only pre-treatment from Satpathy et al. [36]. Dot size corresponds to the activity score (-log10(p)), and dots are colored based on if the observed activity score has a false positive rate below 0.05. Kinases and samples were sorted using hierarchical clustering with ward linkage. Treatment response to combination therapy of chemotherapy and anti-HER2 therapy (responder/pCR vs. non-responder/non-pCR) of each sample are indicated with the context dots above the dotplot. Source data are provided as a separate Source Data file with this paper.

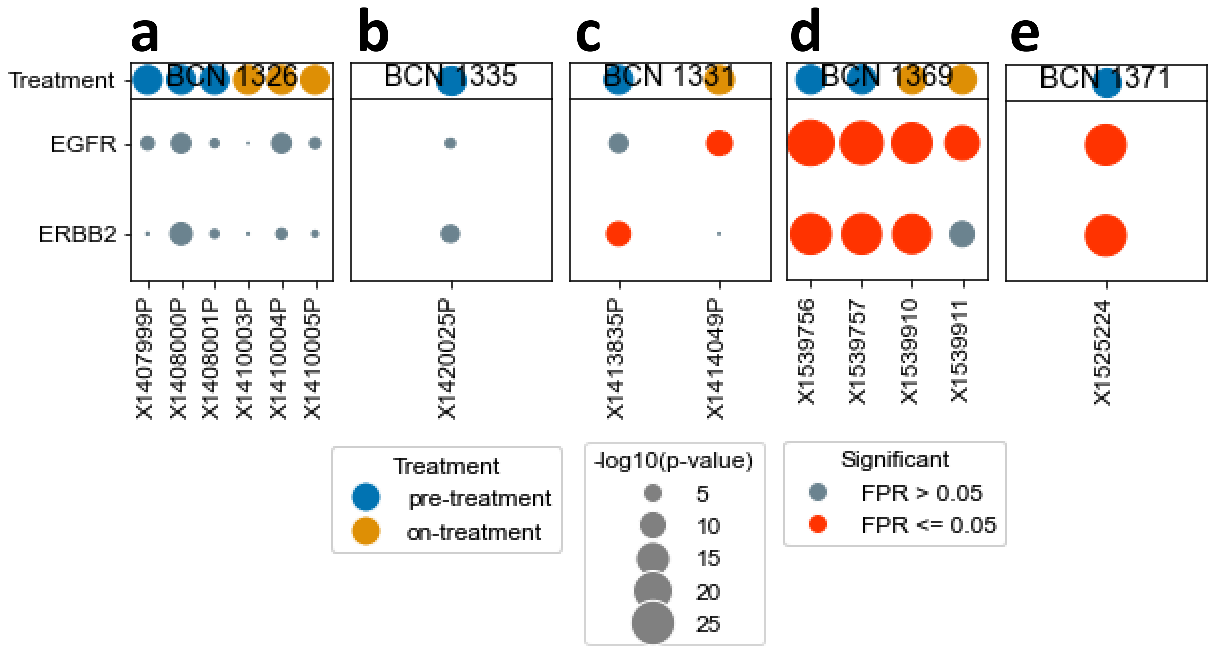

Supplementary Figure 37. Patient-specific KSTAR predictions for non-pathologically complete responders (non-pCR) Patient specific dotplots of EGFR and ERBB2 KSTAR activity predictions for non-pathologically complete responders (non-pCR) to treatment with chemotherapy and anti-HER2 therapy from Satpathy et al. [36]. Each plot includes all available replicates and both pre/post-treatment samples. **A)** Patient BCN1326, a false positive without ERBB2 copy number amplification. **B)** Patient BCN1331, a pseudo-false positive with ERBB2 amplification but without increased protein expression. **C)** Patient BCN1335, a pseudo-false positive with ERBB2 amplification but without increased protein expression. Post-treatment data was not available. **D)** Patient BCN1369, who failed to respond to treatment despite having ERBB2 amplification and increased protein expression. **E)** Patient BCN1371, who failed to respond to treatment despite having ERBB2 amplification and increased protein expression. Post treatment data was not available. Source data are provided as a separate Source Data file with this paper.

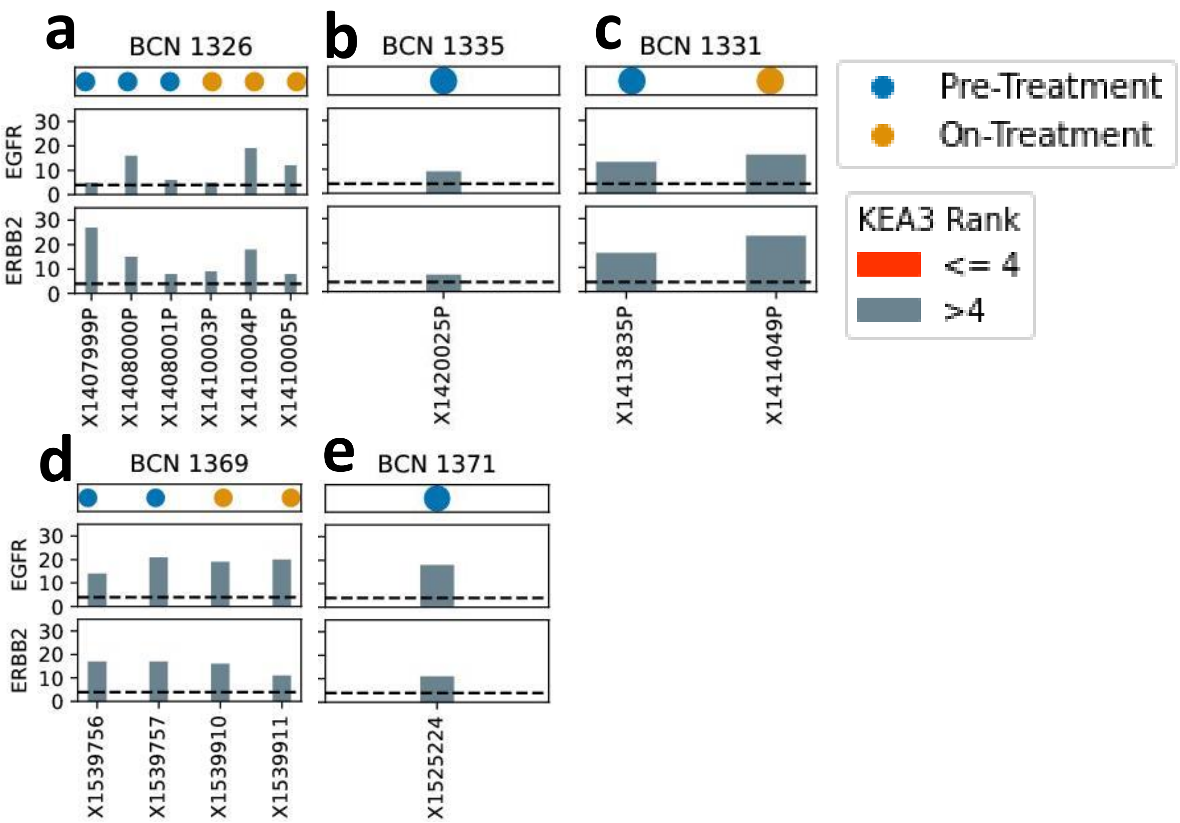

Supplementary Figure 38. Patient-specific KEA3 ranks for non-pathologically complete responders (non-pCR) Patient specific dotplots of EGFR and ERBB2 KEA3 ranks for non-pathologically complete responders to treatment with chemotherapy and anti-HER2 therapy from Satpathy et al. [36]. Ranks are relative to the 50 tyrosine kinases found in NetworKIN in order to make ranks comparable to KSTAR. Each plot includes all available replicates and both pre/post-treatment samples. As all but one true HER2+ sample (not identified as a false positive by Satpathy et al. [36] had ERBB2 as one of the top 4 most active kinases based on KSTAR predictions, we have applied a significance cutoff of rank 4 (rank is 4 or better, kinase is deemed significantly active). See Supplementary Figure 41 for more details on this cutoff choice. **A)** Patient BCN1326, a false positive without ERBB2 copy number amplification. **B)** Patient BCN1331, a pseudo-false positive with ERBB2 amplification but without increased protein expression. **C)** Patient BCN1335, a pseudo-false positive with ERBB2 amplification but without increased protein expression. Post-treatment data was not available. **D)** Patient BCN1369, who failed to respond to treatment despite having ERBB2 amplification and increased protein expression. **E)** Patient BCN1371, who failed to respond to treatment despite having ERBB2 amplification and increased protein expression. Post treatment data was not available. Source data are provided as a separate Source Data file with this paper.

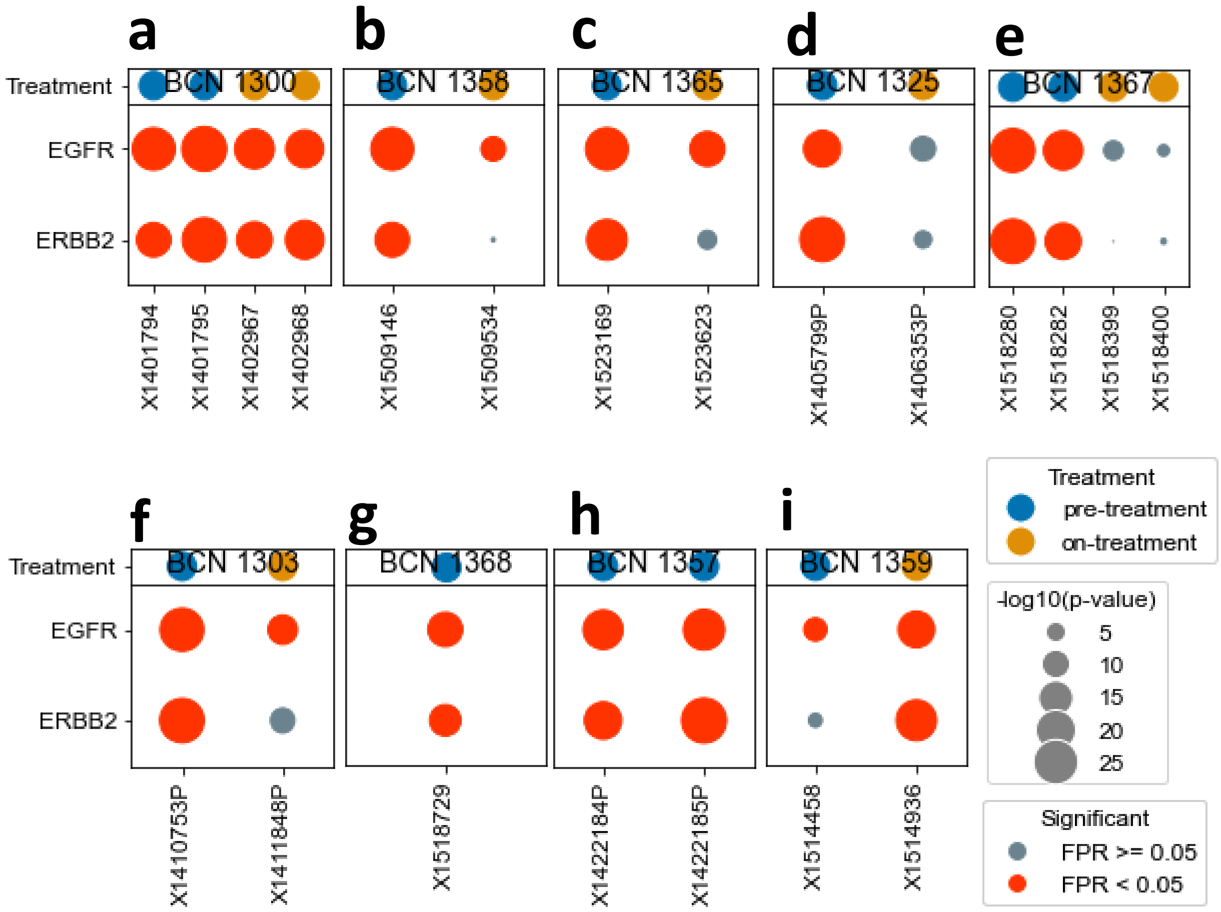

Supplementary Figure 39. Patient-specific KSTAR predictions for pathologically complete responders (pCR) Patient specific dotplots of EGFR and ERBB2 KSTAR activity predictions for pathologically complete responders (pCR) to treatment with chemotherapy and anti-HER2 therapy from Satpathy et al. [36]. Each plot includes all available replicates and both pre/post-treatment samples. **A)** Patient BCN1300. This is the only pCR patient who had basal ERBB2 activity but did not see activity decrease post-treatment, indicating that ERBB2 therapy arm was not responsible for successful response. **B)** Patient BCN1358. **C)** Patient BCN1365. **D)** Patient BCN1325. **E)** Patient BCN1367. **F)** Patient BCN1303. **G)** Patient BCN 1368. Post treatment data not available. **H)** Patient BCN1357. Post treatment data not available. I) Patient BCN1359. This patient exhibited an interesting response to treatment, where ERBB2 mRNA levels actually increased upon treatment, perhaps explaining the increase in predicted activity. Source data are provided as a separate Source Data file with this paper.

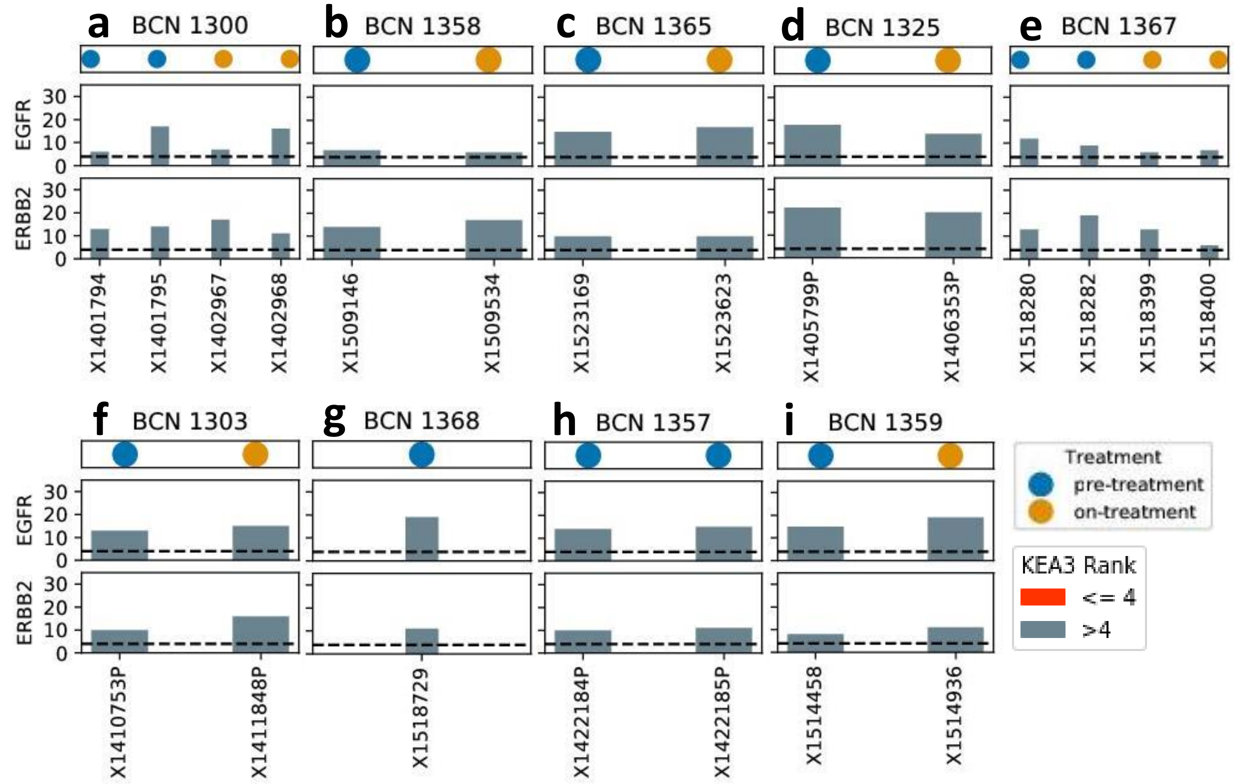

Supplementary Figure 40. Patient-specific KEA3 ranks for pathologically complete responders (pCR) Patient specific dotplots of EGFR and ERBB2 KEA3 ranks for non-pathologically complete responders to treatment with chemotherapy and anti-HER2 therapy from Satpathy et al. [36]. Ranks are relative to the 50 tyrosine kinases found in NetworKIN in order to make ranks comparable to KSTAR. Each plot includes all available replicates and both pre/post-treatment samples. As all but one true HER2+ sample (not identified as a false positive by Satpathy et al [36] had ERBB2 as one of the top 4 most active kinases based on KSTAR predictions, we have applied a significance cutoff of rank 4 (rank is 4 or better, kinase is deemed significantly active). See Supplementary Figure 41 for more details on this cutoff choice. **A)** Patient BCN1300. This is the only pCR patient who had basal ERBB2 activity but did not see activity decrease post-treatment, indicating that ERBB2 therapy arm was not responsible for successful response. **B)** Patient BCN1358. **C)** Patient BCN1365. **D)** Patient BCN1325. **E)** Patient BCN1367. **F)** Patient BCN1303. **G)** Patient BCN 1368. Post treatment data not available .**H)** Patient BCN1357. Post treatment data not available. **I)** Patient BCN1359. This patient exhibited an interesting response to treatment, where ERBB2 mRNA levels actually increased upon treatment, perhaps explaining the increase in predicted activity. Source data are provided as a separate Source Data file with this paper.

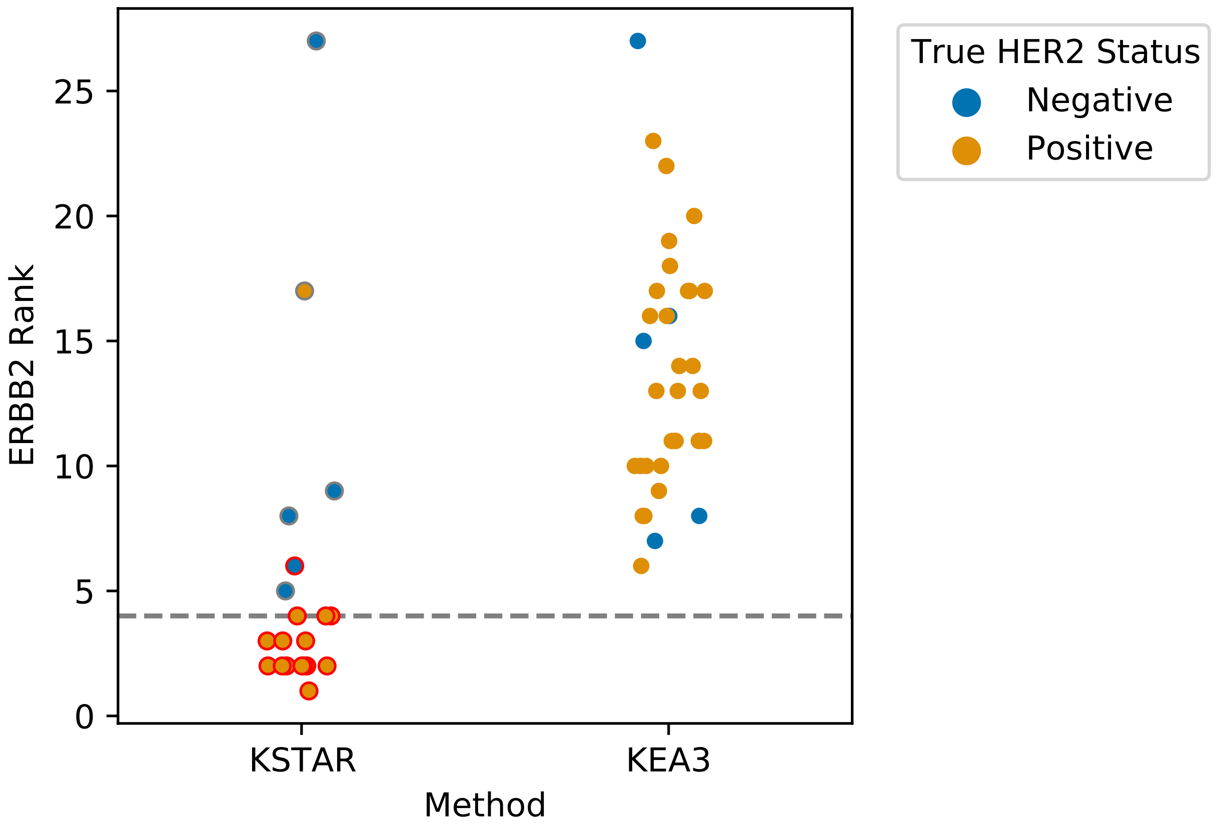

Supplementary Figure 41. Comparing HER2/ERBB2 activity ranks in KSTAR and KEA3 for microscaled biopsies The activity rank of HER2/ERBB2 in the pre-treatment microscaled biopsies of HER2+ patients from Satpathy et al. [36], obtained using either KSTAR activity scores or KEA3 mean ranks. Patients are colored according to their true HER2 positivity status identified in Satpathy et al. (BCN1326, BCN1331, BCN1335 were all identified as false positives, and therefore are colored as negative in this plot). All patient replicates are included. As KSTAR uses false discovery rate as the indicator of activity, KSTAR predictions where FDR ≤ 0.05 are indicated with a red edge. Lastly, all but one true HER2 positive patient sample, BCN1359, identified ERBB2 as one of the top 4 most active kinases. Notably, ERBB2 did not appear in the top 4 most enriched kinases in any KEA3 predictions, and there does not appear to be a clear distinction between the ranks of the true postives and the negative samples. Source data are provided as a separate Source Data file with this paper.

Supplementary Tables

Supplementary Table 1. Commonly used and available kinase activity inference methods We have compiled information regarding several popular/commonly referenced kinase activity/enrichment inference methods currently available. Included is a brief description of their implementation, as well as the input data type, background networks used, whether they use quantification, and if they account for study bias.

PMID	Year	Algorithm Name	Data Input	Kinase-Substrate Info Used	Quantification Required	Single Sample	Account for Study Bias	Platform	Number of times cited in PubMed as of Dec. 2021
23532336, 28655153	2013, 2017	KSEA	Phosphoproteomics	PhosphoSitePlus and NetworKIN	Yes	No	No	R or web-based application:	116, 33
34019655	2021	KEA3	Transcriptomics, Proteomics, or Phosphoproteomics	11 PPI Databases	No	Yes	No	Web-based application	1 (original KEA paper cited 105 times)
30979792	2019	INKA	Phosphoproteomics	PhosphositePlus and NetworKIN	Yes	Yes	No	R or web-based application:	13
28674151	2017	KARP	Phosphoproteomics	PhosphositePlus	Yes	Yes	Yes	Excel VBA, but not published	7
26628587	2016	IKAP	Phosphoproteomics and Proteomics	PhosphositePlus	Yes	No	No	Matlab	23
30563849	2019	PTM-SEA	Phosphoproteomics	PTMsigDB (utilizes information from PhosphoSitePlus)	Yes	No	No	Hosted on tinyurl (GenePlatform), source code available on github	30

Supplementary Table 2. Control experiments used for Figure 2 For each of the datasets used in Figure 2 of the main text, we have provided a description of the original experiment and how it was used in KSTAR analyses, including the threshold used to define binary evidence within them and the resulting evidence sizes.

Source	Short-Hand Name	Cell Line or Tissue	ST/Y or both?	Description of experiment	Conditions	Number of pTyr (total)	Number of pSer/pThr (total)	Threshold used	Quantification meaning	Range of pTyr in post-thresholded experiments	Range of pSer/pThr in post-thresholded experiments
[10]	EGF_ HMEC	Cell line (HMEC, 184A1)	Y	EGF stimulation of HMEC cells	0, 1, 2, 4, 8, 16, 32 minutes of EGF stimulation	228	0	1	Relative to four minutes post-EGF stimulation	9-194	NA
[11]	EGF_ HER2_ HMEC	Cell line (184A1 and 24H 184A1)	Y	EGF and HRG stimulation of HMEC cell lines, one of which overexpresses HER2	HRG or EGF stimulation at 0, 5, 10 and 30 minutes of normal or HER2 overexpressin g cells	66	0	0.8	Relative to P_EGF at 5'	13-66	NA
[12]	TCR	Cell line (Jurkat)	Y	Early T-cell Signaling, activated by anti-CD28 antibody.	0, 5, 15, 30, 60 seconds of activation	665	0	0.2	Relative to unstimulated (0-sec) condition (log2)	128-200	NA
[13]	BCR-ABL	Cell Line (K562)	Y	BCR-ABL driven CML cell line (K562) treated with dasatinib.	Pre-treatment, 20 minutes post-treatment, 3hrs post washout, 6hrs post washout	161	0	0.5	Relative to pre-treatment	81-161	NA
[14]	BRAF	Cell Line (Colo205, HCT116)	ST	CRC cell lines treated with vemurafenib for an hour	Measured every 5 minutes over the course of an hour, for both HCT116 and Colo205 cell lines	15	6851	0	Relative to a DMSO control at each timepoint (and then mean centered across timepoints)	NA	1590-2785
[4]	AKT_Inhib	Cell Line (BT-474)	ST	BT-474 cells treated with five different 1 uM AKT inhibitors for 1 hour	Untreated, MK-2206, Ipatasertib, GSK2110183, GSK690693, AZD5363	0	10345	1	Relative to control/pre-treatment	NA	5069-8980

Supplementary Table 3. Phosphoproteomic datasets used in the tyrosine kinase benchmarking dataset Information about each study/condition used to benchmarking tyrosine kinase predictions, including the kinases considered to be perturbed. We have indicated the file name/location of the source data in the original publication from which the data was obtained. In cases where chemoproteomic information was available, binding information was used to determine the top 5 kinases most likely to be perturbed by drug.

Exp #	Source	Study Name	Location of Data in Source Publication	Chemo proteomics?	Tyrosine Enriched?	Condition	Kinases	Direction
1	[13]	Dasatinib_inhibition_ Asmussen	Table S1	No	Yes	K562 cells treated with dasatinib for 20 minutes	ABL1	Down
2	[10]	EGF_WolfYadlin2007	Table S3	No	Yes	Human mammary epithelial cells stimulated with EGF for 1 minute	EGFR	Up
3	[10]	EGF_WolfYadlin2007	Table S3	No	Yes	Human mammary epithelial cells stimulated with EGF for 4 minutes	EGFR	Up
4	[10]	EGF_WolfYadlin2007	Table S3	No	Yes	Human mammary epithelial cells stimulated with EGF for 8 minutes	EGFR	Up
5	[10]	EGF_WolfYadlin2007	Table S3	No	Yes	Human mammary epithelial cells stimulated with EGF for 16 minutes	EGFR	Up
6	[11]	HER2overexpression_ WolfYadlin2006	Supplementary Table	No	Yes	HER2 overexpressing epithelial cells stimulated with EGF for 10	EGFR	Up
7	[11]	HER2overexpression_ WolfYadlin2006	Supplementary Table	No	Yes	HER2 overexpressing epithelial cells stimulated with HRG for 10	EGFR,ERBB2	Up
8	[12]	TCRstimulation_Chylek	Table S1	No	Yes	TCR stimulation for 5 seconds	LCK, FYN, ZAP70, ITK	Up
9	[12]	TCRstimulation_Chylek	Table S1	No	Yes	TCR stimulation for 15 seconds	LCK ,FYN, ZAP70, ITK	Up
10	[12]	TCRstimulation_Chylek	Table S1	No	Yes	TCR stimulation for 30 seconds	LCK, FYN, ZAP70, ITK	Up
11	[12]	TCRstimulation_Chylek	Table S1	No	Yes	TCR stimulation for 60 seconds	LCK,FYN,ZAP70, ITK	Up
12	[24]	TKIs_Giansanti	SI Table 2, SI Table 3	Yes	Yes	A431 cells treated with imatinib for 2 hours, a TKI inhibitor	CSK, SRC, DDR1	Down
13	[24]	TKIs_Giansanti	SI Table 2, SI Table 3	Yes	Yes	A431 cells treated with dasatinib for 2 hours, a TKI inhibitor	EPHA2, SRC, DDR1, YES1, FRK	Down
14	[24]	TKIs_Giansanti	SI Table 2, SI Table 3	Yes	Yes	A431 cells treated with bosutinib for 2 hours, a TKI inhibitor	EPHA2, EPHB2, FRK, PTK2B, LYN	Down
15	[24]	TKIs_Giansanti	SI Table 2, SI Table 3	Yes	Yes	A431 cells treated with nilotinib for 2 hours, a TKI inhibitor	DDR1, EPHA2, CSK, SRC, PTK2B	Down
16	[25]	VEGF_Zhuang	Table S1	No	Yes	HUVEC cells treated with VEGF for 10 minutes	FLT1, KDR, FLT4	Up
17	[25]	VEGF_Zhuang	Table S1	No	Yes	HUVEC cells treated with VEGF for 20 minutes	FLT1, KDR, FLT4	Up
18	[25]	VEGF_Zhuang	Table S1	No	Yes	HUVEC cells treated with VEGF for 30 minutes	FLT1, KDR, FLT4	Up
19	[21]	Dasatinib_inhibition_Pan	Table S5	No	No	K562 cells treated with 5nm dasatinib for 1 hour	ABL1	Down
20	[21]	Dasatinib_inhibition_Pan	Table S5	No	No	K562 cells treated with 50nm dasatinib for 1 hour	ABL1	Down

Supplementary Table 4. Phosphoproteomic datasets used in the serine/threonine kinase benchmarking dataset Information about each study/condition used to benchmark serine/threonine kinase predictions, including the kinases considered to be perturbed. We have indicated the file name/location of the source data in the original publication from which the data was obtained. In cases where chemoproteomic information was available, binding information was used to determine the top 5 kinases most likely to be perturbed by drug.

Exp #	Source	Study Shorthand	Location of Data in Source Publication	Chemo proteomics?	Condition	Kinases	Direction
21	[4]	AKTinhibition_ Wiechmann	Table S3A	Yes	BT-474 cells treated with 1uM AZD5363 for 1 hour	RPS6KB1,AKT1, ROCK2,EIF2AK4, PRKX	Down
22	[4]	AKTinhibition_ Wiechmann	Table S3A	Yes	BT-474 cells treated with 1uM GSK2110183 for 1 hour	AKT1,PAK6,CLK1, MARK3, PRKACA	Down
23	[4]	AKTinhibition_ Wiechmann	Table S3A	Yes	BT-474 cells treated with 1uM GSK690693 for 1 hour	PDPK1,ROCK1, CDC42BPB,AKT1, CDC42BPG	Down
24	[4]	AKTinhibition_ Wiechmann	Table S3A	Yes	BT-474 cells treated with 1uM Ipatasertib for 1 hour	AKT1,DYRK1A,PRKCH, CLK2,ROCK2	Down
25	[4]	AKTinhibition_ Wiechmann	Table S3A	Yes	BT-474 cells treated with 1uM MK-2206, an AKT inhibitor, for 1 hour	AKT1,AKT2	Down
26	[15]	ATRinhibition_ Salovska	Supplementary File 1	No	HL60 cells treated with VE-821, an ATR inhibitor, after irradiation	ATR	Down
27	[16]	AURK_CellCycleStudy _ Kellenbach	Table S1	No	HeLa cells arrested in mitosis and treated with 0.25uM MLN8054	AURKA	Down
28	[16]	AURK_CellCycleStudy _ Kellenbach	Table S1	No	HeLa cells arrested in mitosis and treated with 1uM MLN8054	AURKA	Down
29	[16]	AURK_CellCycleStudy _ Kellenbach	Table S1	No	HeLa cells arrested in mitosis and treated with 5uM MLN8054	AURKA,AURKB	Down
30	[16]	AURK_CellCycleStudy _ Kellenbach	Table S1	No	Hela cells arrested in mitosis and treated with AZD1152	AURKB	Down
31	[16]	AURK_CellCycleStudy _ Kellenbach	Table S1	No	HeLa cells arrested in mitosis and treated with BI2536	PLK1,PLK2,PLK3	Down
32	[17]	CDK8/19_inhibition_ Poss	Table S1	No	HCT116 cells treated with cortistatinA	CDK8,CDK19	Down
33	[18]	CK2_inhibition_ Franchin	Tables S1-S6	No	HEK293T cells treated with quinalizarin for 3 hours	CSNK2A1, CSNK2A2,CSNK2B	Down
34	[19]	EGF_IGF_ Wilkes	Dataset S2	No	MCF7 cells stimulated with EGF for 5 minutes	MAPK1,MAPK3, BRAF,RAF1,AKT1,MAP2K1,MAP2	Up
35	[19]	EGF_IGF_ Wilkes	Dataset S2	No	MCF7 cells stimulated with EGF for 10 minutes	MAPK1,MAPK3, BRAF,RAF1,AKT1,MAP2K1,MAP2	Up
36	[19]	EGF_IGF_ Wilkes	Dataset S2	No	MCF7 cells stimulated with EGF for 30 minutes	MAPK1,MAPK3, BRAF,RAF1,AKT1,MAP2K1,MAP2	Up
37	[19]	EGF_IGF_ Wilkes	Dataset S2	No	MCF7 cells stimulated with EGF for 60 minutes	MAPK1,MAPK3, BRAF,RAF1,AKT1,MAP2K1,	Up
38	[19]	EGF_IGF_ Wilkes	Dataset S2	No	MCF7 cells stimulated with IGF1 for 5 minutes	MAPK1,MAPK3, BRAF,RAF1,AKT1,MAP2K1, MAP2K2	Up

39	[19]	EGF_IGF_ Wilkes	Dataset S2	No	MCF7 cells stimulated with IGF1 for 10 minutes	MAPK1,MAPK3, BRAF,RAF1,AKT1,MAP2K1, MAP2K2	Up
40	[19]	EGF_IGF_ Wilkes	Dataset S2	No	MCF7 cells stimulated with IGF1 for 30 minutes	MAPK1,MAPK3, BRAF,RAF1,AKT1,MAP2K1, MAP2K2	Up
41	[19]	EGF_IGF_ Wilkes	Dataset S2	No	MCF7 cells stimulated with IGF1 for 60 minutes	MAPK1,MAPK3,BRAF,RAF1, AKT1,MAP2K1,MAP2K2	Up
42	[20]	CDK1_inhib_ Petrone	Table S1	No	HeLa cells treated with Flavopiridol, a CDK1 inhibitor	CDK1	Down
43	[20]	CDK1_inhib_ Petrone	Table S1	No	HeLa cells treated with RO-3306, a CDK1 inhibitor	CDK1	Down
44	[21]	MEK/p38_inhibition_ Pan	Tables S1 and S2	No	HeLa cells treated with EGF	MAPK1,MAPK3,MAP2K1, MAP2K2,BRAF,RAF1,AKT1	Up
45	[21]	MEK/p38_inhibition_ Pan	Tables S1 and S2	No	HeLa cells treated with U0126, a MEK inhibitor, after EGF stimulation	MAP2K1,MAP2K2,MAPK1, MAPK3	Down
46	[21]	MEK/p38_inhibition_ Pan	Tables S1 and S2	No	HeLa cells treated with EGF	MAPK1,MAPK3,MAP2K1, MAP2K2,BRAF,RAF1,AKT1	Up
47	[21]	MEK/p38_inhibition_ Pan	Tables S1 and S2	No	HeLa cells treated with SB202190, a p38 inhibitor, after EGF stimulation	MAPK11,MAPK14	Down
48	[22]	MEK_BRAF_Inhibition _ Stuart	Tables S1 and S2	No	WM239A cells treated with PLX4032, a BRAF inhibitor	BRAF,MAPK1,MAPK3	Down
49	[22]	MEK_BRAF_Inhibition _ Stuart	Tables S1 and S2	No	WM239A cells treated with AZD6244, a MEK1/2 inhibitor	MAP2K1,MAP2K2,MAPK1, MAPK3	Down
50	[23]	DNA_damage_Beli	Table S1	No	U2OS cells treated with epotoside for 24 hours	ATR,ATM	Up
51	[23]	DNA_damage_Beli	Table S1	No	U2OS cells treated with ionizing radiation for 1 hour	ATR,ATM	Up

Supplementary Table 5. Phosphoproteomic datasets used for robustness analysis in Figure 5 For each of the datasets used in Figure 5 of the main text, we have provided a description of the original experiment, the conditions/cell lines we used from this experiment, and the phosphorylation sites used for prediction

Source	Dataset Number	Cell Line	Primary MS Lab	Description of experiment	Number of Sites Identified
[31]	1	H3255	Comb	Comparison of NSCLC cell lines driven by EGFR activating mutations and/or genomic amplification	466
[32]	2	H3255	Comb	EGF and HRG stimulation of HMEC cell lines, one of which overexpresses HER2	444
[31]	3	HCC827	Comb	Comparison of NSCLC cell lines driven by EGFR activating mutations and/or genomic amplification	469
[32]	4	HCC827	Comb	BCR-ABL driven CML cell line (K562) treated with dasatinib.	452
[33]	5	H3255	Comb	Profile of multiple different well-studied cell lines, used to test INKA algorithm	414
[34]	6	HCC827	Jimenez	Profile of multiple different wellstudied cell lines, used to test INKA algorithm	2586
[35]	7	H3255	Pandey	NSCLC cell lines with varying sensitivity to erlotnib treatment treated with either erlotnib or afatanib	189
[3]	8	K562	Heck	Comparison of phosphotyrosine (pY) sites identified via two different enrichment approaches (pY immunaffinity enrichment vs. 2-dimensional approach using metal-affinity based enrichment)	167
[13]	9	K562	Shah	K562 cells treated with dasatinib	161
[34]	10	K562	Jiminez	Profile of multiple different wellstudied cell lines, used to test INKA algorithm	1185
[3]	11	K562	Heck	Comparison of phosphotyrosine (pY) sites identified via two different enrichment approaches (pY immunaffinity enrichment vs. 2-dimensional approach using metal-affinity based enrichment)	1414

Supplementary Table 6. Breast cancer datasets used in Figure 6 For each of the datasets used in Figure 6 of the main text, we have provided a description of the original experiment and how it was used for KSTAR analysis

Source	Short-hand Name	Cell Line or Tissue	ST/Y or Both	Description of Experiment	Conditions Used	Number of pTyr (total	Number of pSer/pThr (total)	Threshold used	Quantification meaning	Range of pTyr in post-thresholded experiments	Range of pSer/pThr in post-thresholded experiments
[36]	Microbiospy	Tissue (Breast cancer)	Both	Performed extensive proteogenomic measurements for 14 HER2+ breast cancer patient samples before and after treatment with a combination of HER2 inhibitors and chemotherapy, where some patients were unresponsive to treatment.	14 patients with pre-treatment, 10 patients with post-treatment, some with multiple replicates	139	23,298	-0.2 (Y), 1 (ST)	Relative to a common reference sample	32-60	1945-2874
[37]	BRCA	Tissue (Breast cancer)	Both	CPTAC: Large scale profiling of 107 different breast cancer patients with associated HER2 clinical status	77 patients with high quality samples	177	26,111	-0.5	Relative to a global pooled sample	35-109	NA
[8]	PDX	Tissue (PDX Breast Cancer)	Both	Measurements for 25 PDX models of breast cancer, 3 of which were identified as HER2+	Pre-treatment measurements for all PDX models	563	48,330	0	Expression was median centered across samples	72-164	NA

Supplementary Table 7. Ancestry, age, and sex of the cell lines used in studies analyzed by KSTAR For all cell-line based data (Figures 2-5), we have included information regarding the ancestry and demographic informaiton of the cell lines used. All information was obtained from Cellosaurus [38]. For patient focused demographic information, please reference the original publications (Figure 6A: Supplementary Table 1 in Mertins et al. [37], Figure 6B: Supplementary Data 1 in Huang et al. [8], Figure 6C: Supplementary Table1B from Satpathy et al. [36]).

Cell Line	RRID Accession	Tissue/Disease	Sex of cell	Age at sampling	Genetic Ancestry	Associated Figure(s)
184A1	CVCL_3040 (Secondary: CVCL_8222)	Human mammary epithelial cells	Female	21	Not Available	Figures 2-4
Jurkat	CVCL_0065	T-cell acute lymphoblastic leukemia	Male	14	European (north): 70.73%, European (south): 26.45%, East Asian (south): 1.21%, South Asian: 1.08%, African: 0.4%, East Asian (north): 0.13%	Figures 2-4
K562	CVCL_0004	Chronic myeloid leukemia	Female	53	European (north): 43.44%, European (south): 42.97%, East Asian (north): 5.19%, African: 5.19%	Figures 2-5
BT-474	CVCL_0179	Invasive breast carcinoma	Female	60	European (north): 70.19%, European (south): 26.74%. East Asian (north): 2.47%, African: 0.6%	Figures 2-4
Colo205	CVCL_0218	Colon adenocarcinoma	Male	70	European (north): 61.39%, European (south): 36.53%, East Asian (north): 2.08%	Figure 2
HCT116	CVCL_0291	Colon carcinoma	Male	48	European (north): 64.85%, European (south): 32.93%, East Asian (north): 1.16%, South Asian: 0.97%, Native American: 0.08%	Figure 2
A431	CVCL_0037	Skin squamous cell carcinoma	Female	85	European (south): 65.16%, European (north): 17.15%, East Asian (north): 7.84%, South Asian: 6.03%, African: 3.82%	Figures 3-4
HL60	CVCL_0002	Acute myeloid leukemia	Female	36	European (north): 71.71%, European (south): 26.32%, African: 1.15%, Native American: 0.42%, East Asian (north): 0.4%	Figures 3-4
HeLa	CVCL_0030	HPV-related endocervical adenocarcinoma	Female	30	African: 64.74%, European (north): 19.45%, European (south): 12.78%, East Asian (north): 2.26%, Native American: 0.77%	Figures 3-4
HEK293T	CVCL_0063	Fetal kidney	Female	Fetus	Not available	Figures 3-4
MCF7	CVCL_0031	Invasive breast carcinoma	Female	69	European (north): 56.91%, European (south): 38.15%, East Asian (north): 4.2%, African: 0.74%	Figures 3-4
WM239A	CVCL_6795	Melanoma	Female	55	Not available	Figures 3-4
U2OS	CVCL_0042	Osteosarcoma	Female	15	European (north): 60.98%, European (south): 31.08%, East Asian (north): 5.19%, African: 2.75%	Figures 3-4
K562	CVCL_0004	Chronic myeloid leukemia	Female	53	European (north): 43.44%, European (south): 42.97%, East Asian (north): 5.19%, African: 5.19%	Figures 2-5
HCC827	CVCL_2063	Lung adenocarcinoma	Female	39	European(south): 67.96%, European (north): 19.48%, South Asian: 7.09%, East Asian (north): 3.47%, African: 1.62%, Native American: 0.39%	Figure 5
HCC827-ER3	CVCL_EJ09	Lung adenocarcinoma	Female	39	Derived from parent HCC827	Figure 5
H3255	CVCL_6831	Lung adenocarcinoma	Female	Unknown	European(north): 59.85%, European (south): 34.46%, African: 3.63%, East Asian (north): 2.06%	Figure 5

Supplementary Methods

Content Found In Section

1. **Problems Addressed by the KSTAR Algorithm**
   (a) Issues with Kinase-Substrate Networks
      i. Problem 1: Kinase-substrate annotations are sparse (page 60)
      ii. Problem 2: Using thresholded kinase-substrate prediction networks degrades performance (page 60)
      iii. Problem 3: Thresholding kinase-substrate prediction networks results in "Hub" substrates (page 61)
      iv. Problem 4: Thresholding kinase-substrate prediction networks results in "Hub" kinases and kinases with little associated evidence (page 61-62)
      v. Problem 5: Certain kinases tend to be connected to well studied sites, leading to kinase-specific false positive rates. (page 61-62)
      vi. Problem 6: Kinases from the same family often exhibit high network overlap (connected to the same substrates) in thresholded networks (page 63)
   (b) Challenges with applying kinase activity inference
      i. Problem 7: Most kinase-activity inference algorithms rely on relative quantification (page 63-64)
      ii. Problem 8: Phosphoproteomic experiments tend to identify well-studied sites, leading to experiment specific false positive rates (page 64)
2. **Implementing the KSTAR algorithm**
   (a) Heuristic Prune and Generation of Network Ensemble
      i. Prune algorithm without accounting for kinase-specific study bias (page 64-65)
      ii. Prune algorithm when accounting for kinase-specific study bias (page 65-66)
   (b) Inferring Kinase Activity from Phosphoproteomic Data
      i. Calculating kinase-substrate enrichment in each pruned network (page 66-67)
      ii. Generating random phosphoproteomic experiments (page 67)
      iii. Calculating the final KSTAR activity score (page 68)
      iv. Calculating the false positive rate of the activity score (page 68-69)

Problems Addressed by the KSTAR Algorithm

Issues with Kinase-Substrate Networks

Problem 1: Known kinase-substrate annotations from phosphoproteome databases like PhosphoSitePlus [1] are sparse: only a small fraction of the total phosphoproteome have at least one known connection to a kinase.

Solution: Using information from kinase-substrate prediction networks like NetworKIN [2] would allow for better coverage of the phosphoproteome.

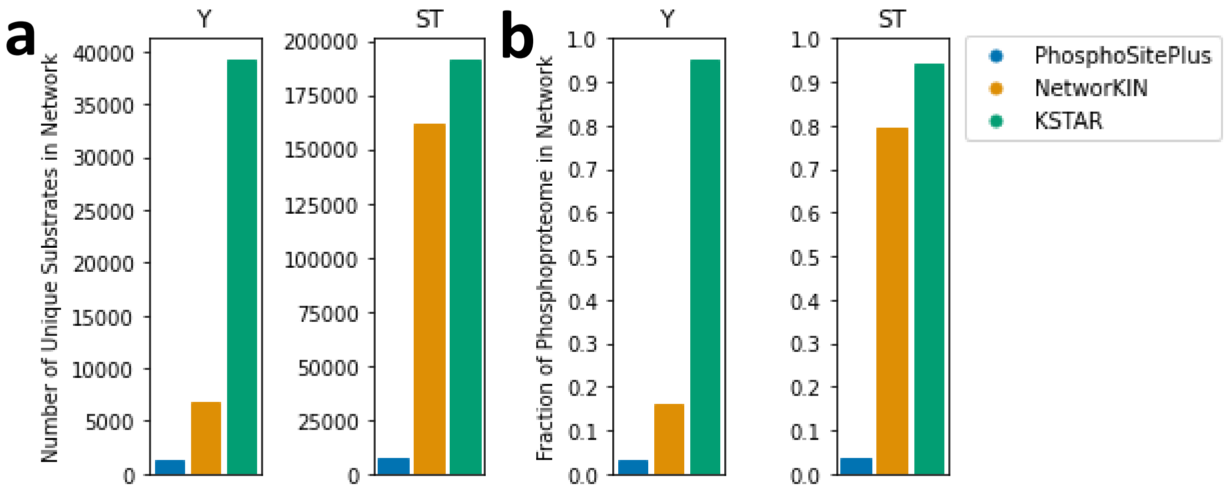

Supplementary Figure 42. KSTAR Expands the Number of Phosphorylation Sites Used in Prediction A) The total number of unique substrates with a edge/interaction with at least one kinase for known kinase-substrate annotations in PhosphoSitePlus [1], or predicted interactions from at hresholded NetworKIN [2], or predicted interactions from KSTAR networks. **B)** The same information as A, but provided as a fraction of the total phosphoproteome. Source data are provided as a separate Source Data file with this paper.

Problem 2: Thresholded kinase-substrate prediction networks have been shown to exhibit poor performance relative to using kinase-substrate annotations alone.

Solution: We hypothesized there is useful information in these kinase-substrate networks, but that the single kinase-substrate network generated by thresholding contains too many incorrect predictions and issues with study bias to be useful. Instead, we proposed that we could create an ensemble of many possible kinase-substrate networks using the network edge weights to guide edge selection, rather than relying on single representation of kinase-substrate relationships (heuristic prune, Algorithm 2). This approach is built on the idea that all networks are wrong, but they are wrong in different ways. While any one single network is unlikely to be the correct representation, aggregating information across these networks will allow us to converge on the kinases most likely to be active. We have demonstrated the utility of ensemble approaches in some of our prior work, such as for clustering with OpenEnsembles [39].

Problem 3: Thresholded kinase-substrate prediction networks result in the emergence of “Hub” substrates, which provide evidence for a disproportionate number of kinases.
Solution: During the heuristic prune, we limit the number of edges each substrate can have in each network (number of kinases it can provide evidence for).

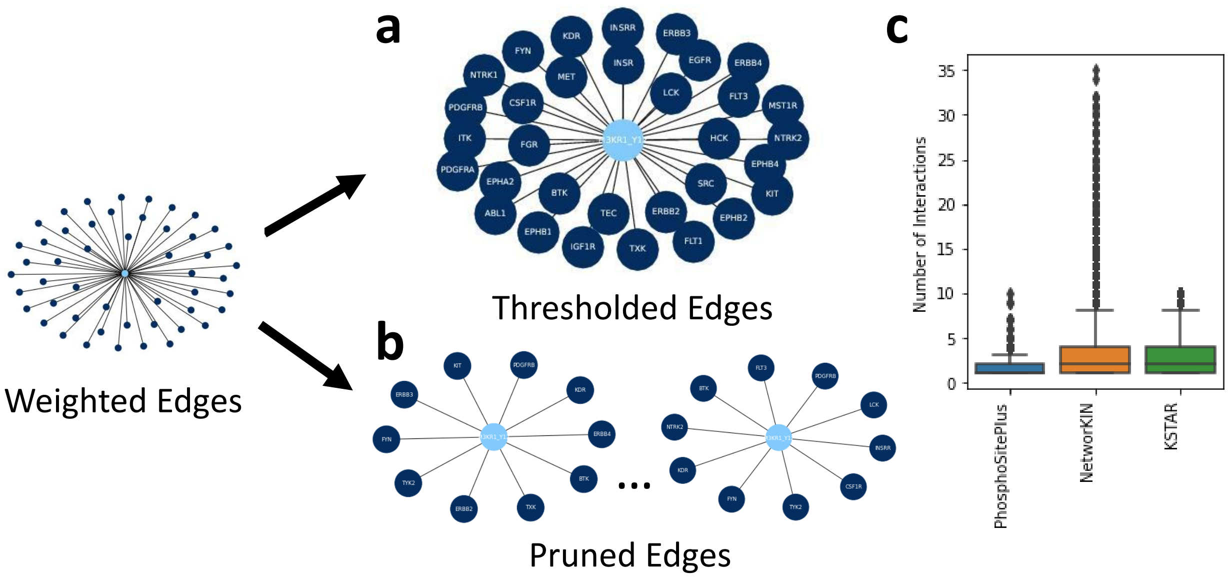

Supplementary Figure 43. Effect of Heuristic Prune on Hub Substrates in NetworKIN Here, we demonstrate the impact of the prune on substrate hubs, using PI3KR1 Y12 as an example (In network graphs, PI3KR1 Y12 is indicated by light blue node, dark blue nodes represent a unique kinase). **A)** Example hub substrate in NetworKIN thresholded with a value of 1 and **B)** the same substrate in two of the 50 pruned networks. **C)** Number of kinases each substrate is connected to in PhosphoSitePlus [1], NetworKIN thresholded with a value of 1 [2], and KSTAR pruned networks [PhosphoSitePlus: n = 1319 tyrosine substrates with at least one known interaction, NetworKIN: n = 6707 tyrosine substrates with at least one predicted interaction, KSTAR; n = 1752023 tyrosine substrates with at least one predicted interaction (only 39,268 unique substrates, each KSTAR network was counted separately to ensure that the properties/distribution of individual networks was illustrated)]. Box indicates median (center line), 25th and 75th percentiles (box boundaries), 1.5x the IQR of the box edge (whiskers), and any outliers beyond 1.5x IQR (points). If no outliers exist, whiskers indicate maxima or minima. Thresholded NetworKIN and pruned KSTAR networks exhibit similar distributions, where the median number of kinases each substrate is connected to is 2. However, KSTAR networks do not have any outlier substrates connected to more than 10 kinases (“Hub” substrates), which can have a large influence on the final predictions. Source data for **c** are provided as a separate Source Data file with this paper.

Problem 4: Thresholded kinase-substrate prediction networks result in the emergence of “Hub” kinases, which are connected to a disproportionate number of substrates. In addition, many kinases often lose the majority of their edges in the network, losing the ability to generate predictions for these kinases.
Solution: During the heuristic prune, force all kinases to have the same number of edges in each network, ensuring that no one kinase has too few/too many substrates providing evidence for them.

Problem 5: Kinase-substrate prediction networks exhibit high study bias, where certain kinases are more likely to be connected to well-studied sites, which leads to kinase-specific false positive rates.
Solution: Enforce a rule during the heuristic prune that ensures every kinase has edges with the same distribution of study bias, as defined by the number of phosphoproteome compendia they are identified in. This means that no one kinase is connected to more well studied sites (site found in most compendia) and no one kinase is connected to more poorly studied sites (site found in few compendia).

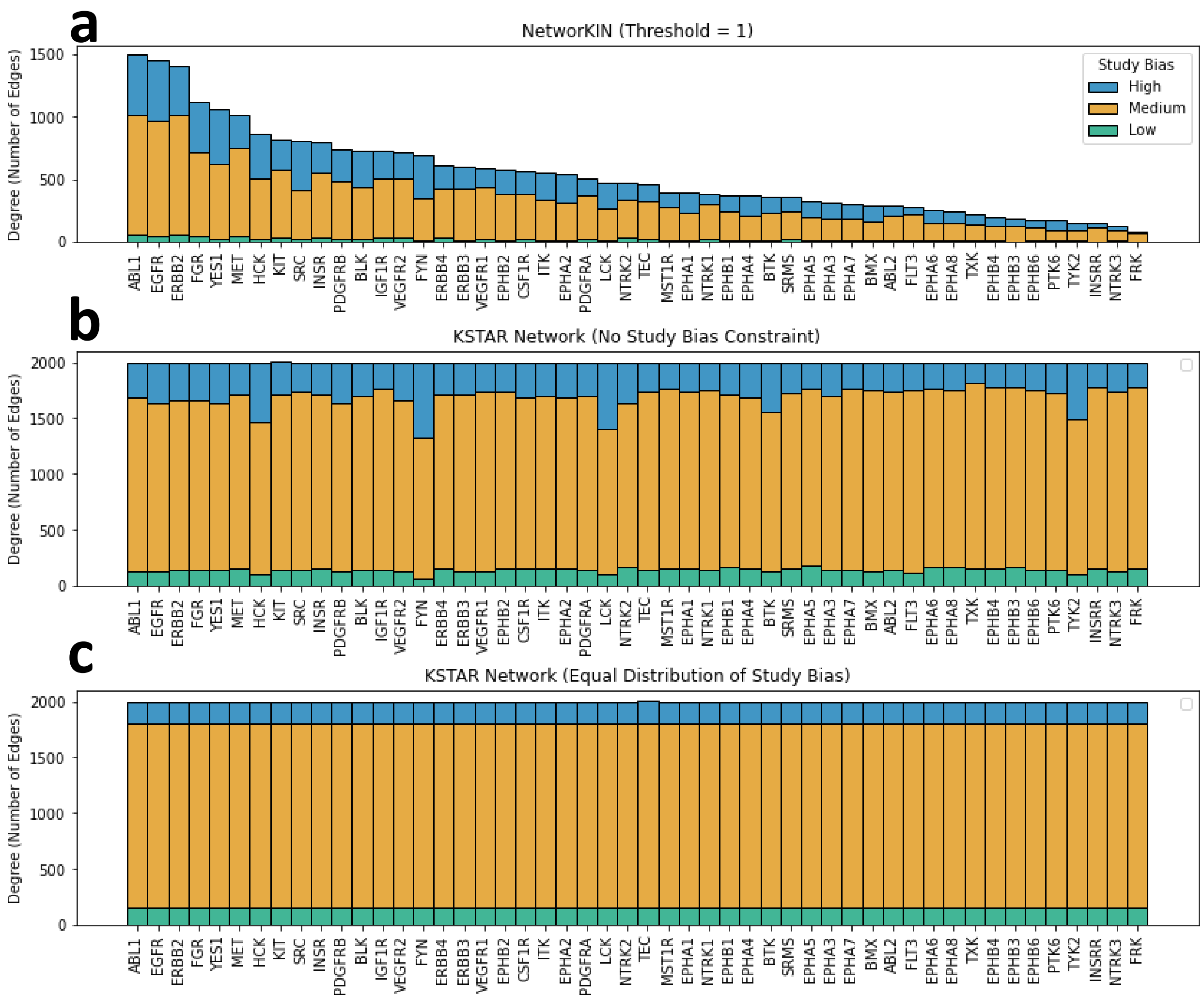

Supplementary Figure 44. KSTAR networks exhibit a more balanced distribution of kinase network edges In order to account for kinase-specific study bias issues in kinase-substrate network (certain kinases are connected to more substrates, or are connected to many well-studied sites), the heuristic prune enforces a rule that ensures all kinases have an equal number of edges in each pruned network, and that these edges have the same distribution of study bias for all kinases (as defined by the number of compendia each substrate is identified in). High study bias sites are found 3-5 compendia, medium study bias sites are found in 1-2 compendia, and low study bias sites are found in 0 compendia (only found in ProteomeScout [40]). **A)** Number of substrates each kinase is connected to in NetworKIN thresholded with a value of 1, and the distribution of study bias within these connections. In addition to certain kinases having significantly more predicted substrates in these networks, we discovered that certain kinases, such as FYN and SRC tended to be connected to more well studied sites. **B)** Number of substrates each kinase is connected to in a pruned network if no study bias constraint is applied (Box 1). While all kinases now have an equal number of edges, certain kinases tend to be more well connected to well studied sites (such as LCK, HCK, and FYN), leading to higher than expected, kinase-specific false positive rates. See Supplementary Note 2 for examples.**C)** Number of substrates each kinase is connected to in one of the pruned KSTAR networks when the study bias constraint is applied (Box 2). Unlike in B, all kinases have the same distribution of study bias among their substrate connections. This is an example of network used for the predictions in the main body of this work. Source data are provided as a separate Source Data file with this paper.

Problem 6: In thresholded kinase-substrate prediction networks, kinases from the same family commonly exhibit high substrate overlap, where many of the same phosphorylation sites provide evidence for the same kinases. This reduces the ability of prediction algorithms to discriminate between the activity of these kinases.

Solution: We have observed that the probability based selection of edges and the constraints placed on this selection (equal distribution of study bias, all kinases have the same number of edges, limits to the number of kinases a substrate can provide evidence for, etc.) during the heuristic prune procedure serve to significantly reduce the overlap between like kinases, allowing for greater discriminability in the activity of these kinases.

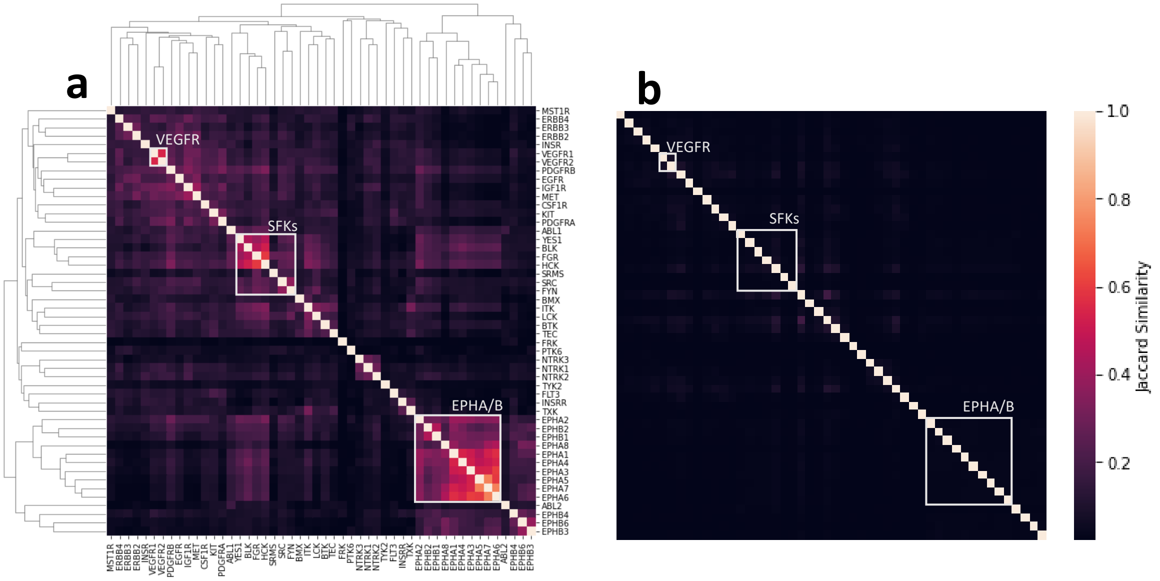

Supplementary Figure 45. Effect of Heuristic Prune on Network Similarity Between Kinases **A)** Heatmap depicting the overlapping substrates between kinases in NetworKIN when thresholded by a value of 1, as defined by the Jaccard similarity index of the sites providing evidence for each kinase. Kinases were sorted using hierarchical clustering. Key kinases/kinase families that exhibit high overlap are indicated by the boxes. **B)** Heatmap depicting the overlapping substrates between the same kinases in KSTAR networks, as defined by the average Jaccard Similarity across all KSTAR networks. Kinases were sorted using the same order in A. Source data are provided as a separate Source Data file with this paper.

Challenges with Kinase Activity Inference

Problem 7: Quantification is required for the majority of currently available kinase activity inference algorithms. This is problematic for several reasons:

1. Relative quantification is inherently noisy, and a fold change can mean different things for different proteins. For example, a fold change of 2 can indicate an increase in abundance from 0.1 to 0.2, but it can also indicate an increase in abundance from 10 to 20, two very different outcomes.
2. More well studied sites tend to exhibit higher magnitude fold changes (See Supplementary Note 2)
3. In the clinical setting, it is often difficult to obtain a matched healthy sample required for relative quantification.

Solution: Measured abundance values are converted to binary evidence based on some cutoff value relevant to the biological problem, and binary evidence is used as input into the KSTAR algorithm. We can also convert to binary evidence based only on whether a site is identified in a sample. This also allows for use of the KSTAR algorithm without relying on any quantification at all, using any sites identified in a sample as evidence, as done for Figure 5 in the main text.

Problem 8: Experiments are more likely to identify well-studied sites, which can lead to experiment-specific false positive rates (see Supplementary Note 2).

Solution: Compare enrichment p-values obtained from the real experiment to enrichment p-values that can be obtained from random phosphoproteomic experiments with the same properties as the real experiment (same number of sites used as evidence, same distribution of study bias). This is done using the Mann Whitney U-test, a non-parametric distribution test.

Implementing the KSTAR algorithm

In this section, we will expand upon our original description of the KSTAR algorithm and how it is implemented in practice. We have seperated the algorithm into two main sections for the purpose of clarity. First, we will describe the heuristic prune and how to generate the ensemble of possible kinase-substrate networks. For this section, the primary data that is required is a weighted kinase-substrate network containing predictions for the entire phosphoproteome, such as NetworKIN [2], GPS [41], or PhosphoPICK [42], and the reference human phosphoproteome obtained from KinPred [9]. The second section describes how phosphoproteomic data is converted into kinase activities using the network ensemble obtained in the first section.

The Heuristic Prune and the Generation of the Network Ensemble

The heuristic prune generates an ensemble of binary kinase-substrate networks representing possible representions of kinase-substrate interactions based on weighted graph predictions (we used NetworKIN, but theoretically can be any weighted kinase-substrate network). In this procedure, we ensure that all kinases have an equal number of edges/interactions in each binary network, set by kinase_network_size. We also ensured that no phosphorylation site provides evidence for more kinases than a predetermined cutoff, site_limit. Lastly, we generate seperate networks for serine/threonine sites and tyrosine sites, as these are nonoverlapping networks. Once these parameters have been determined, edges are randomly selected from the weighted network and added to the pruned network according to a probability equal to edge weights in the weighted network. Importantly, each kinase in the network will have edges added at the same rate (all kinases will have one edge in the pruned network before any have two, etc.), which ensures that every kinase is most likely to have their highest probability edges present in the final networks. If any substrate in the pruned network obtains a number of edges equal to site_limit, it is removed from the weighted network and cannot serve as evidence for any other kinases. Because edge selection is ultimately probabilistic in nature, every network generated using this procedure will be a unique representation of a possible kinase-substrate landscape. The implementation of this approach for a single network is described in Box 1.

Box 1: Heuristic prune of kinase-substrate network without accounting for study bias

Data: Weighted kinase-substrate network

Input: kinase_network_size = number of substrate interactions for each kinase in the pruned network
site_limit = maximum number of kinase interactions for each substrate in the pruned network
modified_sites = type of phosphorylation event to generate network for. Can either be Y (tyrosine) or ST (serine/threonine)

Output: Binary kinase-substrate network

```
Initialize empty binary network;
Reduce weighted network to include either only Y or only S/T sites;
for i = 1, 2, 3, ...,kinase_network_size do
    Shuffle kinase order;
    foreach kinase do
        substrate ← sample from weighted network with probability equal to kinase edge weights;
        Add substrate-kinase edge to binary network;
        Remove substrate-kinase edge from weighted network;
        if degree(substrate) ≥ textsite_limit then
            Remove substrate node from weighted network;
        end if
    end foreach
end for
```

We noticed that the above approach resulted in a high number of false positives for certain kinases and experiments (see Problem 5 and Supplementary Note 2), and determined that this was likely a result of the study bias found within these networks, where well studied substrates tend to have high influence on predictions. To account for study bias, we modified the approach described in Box 1 so that each kinase has edges/interactions with the same distribution of study bias (i.e. no kinases have only well-studied sites providing evidence for them, and no kinases have only poorly studied sites providing evidence for them). To do so, we defined study bias for each substrate in the human phosphoproteome as the number of different phosphoproteome compendia they are identified in. The compendia used in this study are PhosphoSitePlus [1], phosphoELM [5], HRPD [6], dbPTM [7], and UniProt [43].

The heuristic prune described in Box 2 is similar to the procedure in Box 1, except that each kinase in the final binary network contains an equal distribution of sites found in 0, 1, 2, 3, 4, or 5 compendia (Supplementary Figure 44). Box 2 illustrates is the prune implementation we utilize throughout the main body of this work.

Box 2: Heuristic prune of kinase-substrate network that ensures equal distribution of study bias for each kinase

```
Data: Weighted kinase-substrate network
      Human Reference Phosphoproteome from KinPred
Input: kinase_network_size = number of substrate interactions for each kinase in the pruned
       network
       site_limit = maximum number of kinase interactions for each substrate in the pruned
       network
Output: Binary kinase-substrate network

Initialize empty binary_network;
Reduce weighted network to include only Y or S/T sites;
for n = 1, 2, 3, 4, 5 do
    /* In order to know how many sites found in n compendia to sample, we need to
       determine the fraction of these sites found in the overall phosphoproteome.
       Out of the total kinase_network_size, we will then sample that same
       fraction so that each kinase has the same distribution of study bias as
       the background network.                                                   */
    compendia_size ← (number of sites in weighted network found in n compendia / total number of sites in weighted network) * kinase_network_size;
    compendia_network ← reduce weighted network to only include sites found in n compendia;
    /* For each compendia size, n, follow the same pruning procedure as described
       in Box 1.                                                                 */
    for i = 1, 2, 3, ..., compendia_size do
        Shuffle kinase order;
        foreach kinase do
            substrate ← sample from compendia_network with probability equal to kinase edge
                    weights;
            Add substrate-kinase edge to binary network;
            Remove substrate-kinase edge from weighted network;
            if degree(substrate) >= site_limit then
                Remove substrate node from weighted network;
            end if
        end foreach
    end for
end for
```

Calculating Kinase Activity from a Phosphoproteomic Experiment

Once networks have been generated, we are now ready to predict kinase activity based on the phosphorylation sites identified in a mass spectrometry experiment. Prior to enrichment calculations, the list of phosphorylation sites to use as evidence for a particular sample must be determined. In most cases, this is determined by defining a threshold and accepting any phosphorylation sites with abundance values greater than the threshold as evidence. In other cases, we either do not want to use the relative abundance values or do not have relative abundance values (no matched samples), and instead want to use all sites identified in an experiment as evidence (in practice, this equivalent to setting a very low threshold like -1e10).

The first step of activity prediction is to assess the statistical enrichment of each kinase's substrates identified in a sample using the hypergeometric distribution. In this test, for each kinase, we are asking what is the likelihood that there are k substrates of a kinase in the the n phosphorylation sites providing evidence for a sample, based on the total number of substrates (K) and phosphorylation sites (N) in the background phosphoproteome. As there are a total of 50 binary networks in the ensemble, the end result

of this process is to produce 50 p-values for each kinase. The implementation of this procedure is described in Box 3.

Box 3: Calculating statistical enrichment of kinase substrates in binary networks

Data: Phosphoproteomic experiment
Ensemble of pruned networks generated from tje procedure in Box 2
Input: threshold = cutoff value that determines which sites are used as evidence
Output: Arrays with 50 p-values (one for each kinase) containing hypergeometric enrichment results in each of the 50 pruned networks

Intialize p-value array;
binary_experiment $\leftarrow$ reduced experiment including phosphorylation sites with abundance $\geq$ threshold;
foreach kinase **do**
  foreach network **do**
    $k \leftarrow$ number of kinase substrates in binary_experiment;
    $K \leftarrow$ number of kinase substrates in network;
    $n \leftarrow$ number of sites in binary experiment;
    $N \leftarrow$ number of sites in network;
    $p \leftarrow 1 -$ hypergeometric_cdf$(k-1, K, n, N)$;
    Add p to array of p-values;
  end foreach
end foreach

Next, we wished to ask whether, for each kinase, the distribution of p-values obtained from the procedure in Box 3 could be obtained from a random experiment where substrates are randomly pulled from the human phosphoproteome. To do so, we first needed to generate a collection of random experiments. In order to accurately reflect the characteristics of the real dataset, each random experiment must have the same number of observed phosphorylation sites and equal distribution of study bias (defined by the number of compendia a site is identified in). The procedure of generating these random experiments is described in Box 4.

Box 4: Generate random phosphoproteomic experiments for use in Mann Whitney tests

Data: Phosphoproteomic experiment (Real)
Human Phosphoproteome Compendia (KinPred)
Input: num_experiments = number of random experiments to generate
Output: random_array = array of random experiments with identical characteristics as the real experiment (number of phosphorylation sites, distribution of study bias, etc.).

Initialize random_array;
for $i = 1, 2, 3, ...,$ num_experiments **do**
  Initialize random_experiment;
  foreach site identified in real experiment **do**
    numCompendia $\leftarrow$ number of compendia site is found in;
    random_site $\leftarrow$ randomly sampled site from human phosphoproteome compendia found in the same number of compendia as numCompendia;
    Add random_site to random_experiment
  end foreach
  Add random_experiment to random_array
end for

Once random experiments have been generated by the protocol in Box 4, we can generate the final activity scores for each kinase. Ultimately, this score is a reflection of the enrichment of kinase-substrates

in the actual experiment (calculated from protocol in Box 3), relative the enrichment that could be obtained from a random phosphoproteomic experiment. For this task, we use a Mann Whitney U-test, non-parametric distribution-based test which compares the ranked p-values of the real and random experiments. The outcome of this test is a p-value for each kinase, which we then convert to an activity score by taking the -log10 of each p-value. In this way, kinases with high activity (and as a result of high enrichment of substrates in the dataset) will have high activity scores. The application of this test is demonstrated in Box 5.

Box 5: Calculate the final KSTAR activity score using a Mann Whitney U test for an individual kinase

Input: real_p = array of 50 p-values obtained from applying the procedure in Box 3
random_array = 150 random experiments generated from the procedure in Box 4
Output: activity = -log10(MannWhitney p-value)

random_p ← algorithm in Box 3 applied to each random experiment in random_array;
foreach kinase **do**
  MannWhitneyP ← MannWhitneyUtest(real_p_{kinase}, random_p_{kinase});
  $activity_{kinase}$ ← -log10(MannWhitneyP);
end foreach
return activity;

To verify the significance of these scores, we then ask how often we could obtain the same activity scores or better from the random datasets. To do so, we treat one random experiment generated from the procedure in Box 4 as the real experiment and use the procedure in Box 5 to generate a random activity score. We then repeat this procedure to generate an array of random activity scores. The false positive rate is then the fraction of times a random experiment generated an activity score equal to or greater than the activity score calculated for the real dataset.

Box 6: Calculate the false positive rate of obtaining the activity score for a single kinase obtained from Box 5

Input: real_activity = activity score obtained from Box 5
random_array = 150 random experiments generated from Box 4
numTrials = number of random activity scores to obtain for FPR calculation
Output: fpr = fraction of random experiments which obtained the same or greater activity score as real_activity

Initialize numFalsePositives (numFalsePositives = 0);
for $i = 1, 2, 3, ...,$ numTrials **do**
  `/* randomly select one of the random experiments in random_array to be treated as a real experiment in the MannWhitneyU test */`
  pseudoreal ← randomly select experiment from random_array;
  trimrandom ← random_array with pseudoreal removed;
  `/* So, pseudoreal should consist of 1 experiment from random_array, and trimrandom should consist of all other experiments from random_array */`
  MannWhitneyP ← apply Box 5, treating pseudoreal as the real experiment and trimrandom as random experiments;
  random_$activity_i$ ← -log10(MannWhitneyP);
  if random_$activity_i$ ≥ real_activity **then**
    numFalsePositives ← numFalsePositives + 1
  end if
end for
return fpr;

Supplementary References

1. Hornbeck, P. V. *et al.* Phosphositeplus: a comprehensive resource for investigating the structure and function of experimentally determined post-translational modifications in man and mouse. *Nucleic acids research* **40**, D261–70 (2012).

2. Horn, H. *et al.* Kinomexplorer: an integrated platform for kinome biology studies. *Nature Methods 2014 11:6* **11**, 603–604 (2014).

3. Palma, S. D. *et al.* Finding the same needles in the haystack? a comparison of phosphotyrosine peptides enriched by immuno-affinity precipitation and metal-based affinity chromatography. *Journal of Proteomics* **91**, 331–337 (2013).

4. Wiechmann, S. *et al.* Chemical phosphoproteomics sheds new light on the targets and modes of action of akt inhibitors. *ACS Chemical Biology* **16**, 631–641 (2021).

5. Diella, F. *et al.* Phospho.elm: a database of experimentally verified phosphorylation sites in eukaryotic proteins. *BMC bioinformatics* **5** (2004).

6. Prasad, T. S. K. *et al.* Human protein reference database—2009 update. *Nucleic Acids Research* **37**, D767–D772 (2009).

7. Lu, C. T. *et al.* dbptm 3.0: an informative resource for investigating substrate site specificity and functional association of protein post-translational modifications. *Nucleic Acids Research* **41**, D295–D305 (2013).

8. Huang, K. L. *et al.* Proteogenomic integration reveals therapeutic targets in breast cancer xenografts. *Nature Communications* **8**, 1–16 (2017).

9. Xue, B., Jordan, B., Rizvi, S. & Naegle, K. M. Kinpred: A unified and sustainable approach for harnessing proteome-level human kinase-substrate predictions. *PLOS Computational Biology* **17**, e1008681 (2021).

10. Wolf-Yadlin, A., Hautaniemi, S., Lauffenburger, D. A. & White, F. M. Multiple reaction monitoring for robust quantitative proteomic analysis of cellular signaling networks. *Proceedings of the National Academy of Sciences of the United States of America* **104**, 5860–5865 (2007).

11. Wolf-Yadlin, A. *et al.* Effects of her2 overexpression on cell signaling networks governing proliferation and migration. *Molecular Systems Biology* **2**, 54 (2006).

12. Chylek, L. A. *et al.* Phosphorylation site dynamics of early t-cell receptor signaling. *PLoS ONE* **9**, e104240 (2014).

13. Asmussen, J. *et al.* Mek-dependent negative feedback underlies bcr-abl-mediated oncogene addiction. *Cancer discovery* 200–215 (2013).

14. Kubiniok, P., Lavoie, H., Therrien, M. & Thibault, P. Time-resolved phosphoproteome analysis of paradoxical raf activation reveals novel targets of erk. *Molecular and Cellular Proteomics* **16**, 663–679 (2017).

15. Šalovská, B. *et al.* Radiosensitization of human leukemic hl-60 cells by atr kinase inhibitor (ve-821): Phosphoproteomic analysis. *International Journal of Molecular Sciences 2014, Vol. 15, Pages 12007-12026* **15**, 12007–12026 (2014).

16. Kettenbach, A. N. *et al.* Quantitative phosphoproteomics identifies substrates and functional modules of aurora and polo-like kinase activities in mitotic cells. *Science signaling* **4** (2011).

17. Poss, Z. C. *et al.* Identification of mediator kinase substrates in human cells using cortistatin a and quantitative phosphoproteomics. *Cell Reports* **15**, 436–450 (2016).

18. Franchin, C. *et al.* Quantitative analysis of a phosphoproteome readily altered by the protein kinase ck2 inhibitor quinalizarin in hek-293t cells. *Biochimica et Biophysica Acta (BBA) - Proteins and Proteomics* **1854**, 609–623 (2015).

19. Wilkes, E. H., Terfve, C., Gribben, J. G., Saez-Rodriguez, J. & Cutillas, P. R. Empirical inference of circuitry and plasticity in a kinase signaling network. *Proceedings of the National Academy of Sciences of the United States of America* **112**, 7719–7724 (2015).

20. Petrone, A., Adamo, M. E., Cheng, C. & Kettenbach, A. N. Identification of candidate cyclin-dependent kinase 1 (cdk1) substrates in mitosis by quantitative phosphoproteomics. *Molecular and Cellular Proteomics* **15**, 2448–2461 (2016).

21. Pan, C., Olsen, J. V., Daub, H. & Mann, M. Global effects of kinase inhibitors on signaling networks revealed by quantitative phosphoproteomics. *Molecular and Cellular Proteomics* **8**, 2796–2808 (2009).

22. Stuart, S. A. *et al.* A phosphoproteomic comparison of b-rafv600e and mkk1/2 inhibitors in melanoma cells. *Molecular and Cellular Proteomics* **14**, 1599–1615 (2015).

23. Beli, P. *et al.* Proteomic investigations reveal a role for rna processing factor thrap3 in the dna damage response. *Molecular Cell* **46**, 212–225 (2012).

24. Giansanti, P. *et al.* Evaluating the promiscuous nature of tyrosine kinase inhibitors assessed in a431 epidermoid carcinoma cells by both chemical- and phosphoproteomics. *ACS Chemical Biology* **9**, 1490–1498 (2014).

25. Zhuang, G. *et al.* Phosphoproteomic analysis implicates the mtorc2-foxo1 axis in vegf signaling and feedback activation of receptor tyrosine kinases. *Science Signaling* **6** (2013).

26. Casado, P. *et al.* Kinase-substrate enrichment analysis provides insights into the heterogeneity of signaling pathway activation in leukemia cells. *Science Signaling* **6**, rs6–rs6 (2013).

27. Krug, K. *et al.* A curated resource for phosphosite-specific signature analysis. *Molecular and Cellular Proteomics* **18**, 576–593 (2019).

28. Wilkes, E. H., Casado, P., Rajeeve, V. & Cutillas, P. R. Kinase activity ranking using phosphoproteomics data (karp) quantifies the contribution of protein kinases to the regulation of cell viability. *Molecular and Cellular Proteomics* **16**, 1694–1704 (2017).

29. Kuleshov, M. V. *et al.* Kea3: Improved kinase enrichment analysis via data integration. *Nucleic Acids Research* **49**, W304–W316 (2021).

30. Wiredja, D. D., Koyutürk, M. & Chance, M. R. The ksea app: a web-based tool for kinase activity inference from quantitative phosphoproteomics. *Bioinformatics (Oxford, England)* **33**, 3489–3491 (2017).

31. Guo, A. *et al.* Signaling networks assembled by oncogenic egfr and c-met. *Proceedings of the National Academy of Sciences of the United States of America* **105**, 692–697 (2008).

32. Rikova, K. *et al.* Global survey of phosphotyrosine signaling identifies oncogenic kinases in lung cancer. *Cell* **131**, 1190–1203 (2007).

33. Moritz, A. *et al.* Akt - rsk - s6 kinase signaling networks activated by oncogenic receptor tyrosine kinases. *Science Signaling* **3**, 1–12 (2010).

34. Beekhof, R. *et al.* Inka, an integrative data analysis pipeline for phosphoproteomic inference of active kinases. *Molecular systems biology* **15**, e8250 (2019).

35. Zhang, X. *et al.* Quantitative tyrosine phosphoproteomics of epidermal growth factor receptor (egfr) tyrosine kinase inhibitor-treated lung adenocarcinoma cells reveals potential novel biomarkers of therapeutic response. *Molecular and Cellular Proteomics* **16**, 891–910 (2017).

36. Satpathy, S. *et al.* Microscaled proteogenomic methods for precision oncology. *Nature Communications* **11** (2020).

37. Mertins, P. *et al.* Proteogenomics connects somatic mutations to signalling in breast cancer. *Nature* **534**, 55–62 (2016).

38. Bairoch, A. The cellosaurus, a cell-line knowledge resource. *Journal of Biomolecular Techniques* **29**, 25–38 (2018).

39. Ronan, T. *et al.* Openensembles: A python resource for ensemble clustering. *Journal of Machine Learning Research* **18**, 1–6 (2018).

40. Matlock, M. K., Holehouse, A. S. & Naegle, K. M. Proteomescout: A repository and analysis resource for post-translational modifications and proteins. *Nucleic Acids Research* (2015).

41. Wang, C. *et al.* Gps 5.0: An update on the prediction of kinase-specific phosphorylation sites in proteins. *Genomics, Proteomics and Bioinformatics* (2020).

42. Patrick, R., Cao, K.-A. L., Kobe, B. & Bodén, M. Phosphopick: modelling cellular context to map kinase-substrate phosphorylation events. *Bioinformatics* **31**, 382–389 (2015).

43. Bateman, A. *et al.* Uniprot: the universal protein knowledgebase in 2021. *Nucleic Acids Research* **49**, D480–D489 (2021).
