# Supplementary material for: KSTAR: An algorithm to predict patient-specific kinase activities from phosphoproteomic data
Source: Nat Commun. 2022 Jul 25;13:4283. doi: 10.1038/s41467-022-32017-5 (PMC9314348; doi:10.1038/s41467-022-32017-5)
Supplement: Supplementary file 3 — Reporting Summary [file 41467_2022_32017_MOESM3_ESM.pdf]

nature portfolio

Corresponding author(s): Kristen Naegle

Last updated by author(s): 2022/06/27

Reporting Summary

Nature Portfolio wishes to improve the reproducibility of the work that we publish. This form provides structure for consistency and transparency in reporting. For further information on Nature Portfolio policies, see our Editorial Policies and the Editorial Policy Checklist.

Statistics

For all statistical analyses, confirm that the following items are present in the figure legend, table legend, main text, or Methods section.

n/a	Confirmed	
☐	☒	The exact sample size (*n*) for each experimental group/condition, given as a discrete number and unit of measurement
☐	☒	A statement on whether measurements were taken from distinct samples or whether the same sample was measured repeatedly
☐	☒	The statistical test(s) used AND whether they are one- or two-sided<br>*Only common tests should be described solely by name; describe more complex techniques in the Methods section.*
☒	☐	A description of all covariates tested
☐	☒	A description of any assumptions or corrections, such as tests of normality and adjustment for multiple comparisons
☐	☒	A full description of the statistical parameters including central tendency (e.g. means) or other basic estimates (e.g. regression coefficient) AND variation (e.g. standard deviation) or associated estimates of uncertainty (e.g. confidence intervals)
☐	☒	For null hypothesis testing, the test statistic (e.g. *F*, *t*, *r*) with confidence intervals, effect sizes, degrees of freedom and *P* value noted<br>*Give P values as exact values whenever suitable.*
☒	☐	For Bayesian analysis, information on the choice of priors and Markov chain Monte Carlo settings
☒	☐	For hierarchical and complex designs, identification of the appropriate level for tests and full reporting of outcomes
☒	☐	Estimates of effect sizes (e.g. Cohen's *d*, Pearson's *r*), indicating how they were calculated

Our web collection on statistics for biologists contains articles on many of the points above.

Software and code

Policy information about availability of computer code

Data collection	No software was used for data collection
Data analysis	All code for KSTAR are available at our Git repository: https://github.com/NaegleLab/KSTAR with a snapshot of the repository at the time of this work at https://doi.org/10.6084/m9.figshare.20146016. Code for applying KSTAR to data, including all examples within this paper, is available at our KSTAR Applications Git repository: https://github.com/NaegleLab/KSTAR_Applications with a snapshot of the repository at the time of this work at https://doi.org/10.6084/m9.figshare.20146013.

For manuscripts utilizing custom algorithms or software that are central to the research but not yet described in published literature, software must be made available to editors and reviewers. We strongly encourage code deposition in a community repository (e.g. GitHub). See the Nature Portfolio guidelines for submitting code & software for further information.

Data

Policy information about availability of data

All manuscripts must include a data availability statement. This statement should provide the following information, where applicable:

- Accession codes, unique identifiers, or web links for publicly available datasets
- A description of any restrictions on data availability
- For clinical datasets or third party data, please ensure that the statement adheres to our policy

The networks generated in this study have been deposited as data files in Figshare at https://doi.org/10.6084/m9.figshare.14944305. The Resource Files, along with network files, required to reproduce the experiments in this work have been deposited in Figshare at https://doi.org/10.6084/m9.figshare.14885121.v6. All source data have been deposited within the KSTAR Figshare project https://doi.org/10.6084/m9.figshare.14919726 at the permanent doi: https://doi.org/10.6084/m9.figshare.14919726. Source data are also provided as a Source Data file.

Field-specific reporting

Please select the one below that is the best fit for your research. If you are not sure, read the appropriate sections before making your selection.

- [x] Life sciences
- [ ] Behavioural & social sciences
- [ ] Ecological, evolutionary & environmental sciences

For a reference copy of the document with all sections, see nature.com/documents/nr-reporting-summary-flat.pdf

Life sciences study design

All studies must disclose on these points even when the disclosure is negative.

Sample size	The number of networks generated that then give a sample set for the Mann-Whitney test was determined by finding the number of networks required for p-values of all kinases on a sample dataset to stabilize.
Data exclusions	No data were excluded from the analysis.
Replication	We designed our experimental analyses to use multiple datasets from different sources for replication of key findings and all kinase predictions replicated across sources and across replicates (when available) in the same source.
Randomization	Randomization for purposes of measuring a false positive rate and p-value that is based on random expectation was done by selecting random phosphorylation sites that conform to the same distribution of study bias as the experiment under consideration.
Blinding	Blinding was not possible as published data used in this study were labeled.

Reporting for specific materials, systems and methods

We require information from authors about some types of materials, experimental systems and methods used in many studies. Here, indicate whether each material, system or method listed is relevant to your study. If you are not sure if a list item applies to your research, read the appropriate section before selecting a response.

Materials & experimental systems

n/a	Involved in the study	
x		Antibodies
x		Eukaryotic cell lines
x		Palaeontology and archaeology
x		Animals and other organisms
x		Human research participants
x		Clinical data
x		Dual use research of concern

Methods

n/a	Involved in the study	
x		ChIP-seq
x		Flow cytometry
x		MRI-based neuroimaging
